# Anatomical network analyses reveal evolutionary integration and modularity in the lizards skull

**DOI:** 10.1038/s41598-022-18222-8

**Published:** 2022-09-05

**Authors:** Yuya Asakura, Soichiro Kawabe

**Affiliations:** 1 Department of Bioscience and Biotechnology, Fukui Prefectural University, 4-1-1 Matsuoka Kenjojima, Eiheiji, Fukui, 910-1195 Japan; 2 Present Address: Graduate School of Science, The University of Tokyo, 7-3-1 Hongo, Bunkyo, Tokyo, 113-0033 Japan; 3 Institute of Dinosaur Research, Fukui Prefectural University, 4-1-1 Matsuoka Kenjojima, Eiheiji, Fukui, 910-1195 Japan; 4 Fukui Prefectural Dinosaur Museum, 51-11 Terao, Muroko, Katsuyama, Fukui 911-8601 Japan

**Keywords:** Evolution, Zoology

## Abstract

The morphology of lizard skulls is highly diverse, and it is crucial to understand the factors that constrain and promote their evolution to understand how lizards thrive. The results of interactions between cranial bones reflecting these factors can be detected as integration and modularity, and the analysis of integration and modularity allows us to explore the underlying factors. In this study, the integration and modularity of the skulls of lizards and the outgroup tuatara are analyzed using a new method, Anatomical Network Analysis (AnNA), and the factors causing lizards morphological diversity are investigated by comparing them. The comparison of modular structures shows that lizard skulls have high integration and anisomerism, some differences but basically common modular patterns. In contrast, the tuatara shows a different modular pattern from lizards. In addition, the presence of the postorbital bar by jugal and postorbital (postorbitofrontal) also reflect various functional factors by maintaining low integration. The maintenance of basic structures due to basic functional requirements and changes in integration within the modules play a significant role in increasing the morphological diversity of the lizard skull and in the prosperity of the lizards.

## Introduction

The body parts that constitute the whole morphology of an organism, such as vertebrate skulls, are intricately related to each other. The biological processes that produce interactions between the tissues include development, genetics, function, and evolution (e.g., the sharing of developmental origins, pleiotropic gene effects, the movement in the same direction)^1^. For example, vertebrate skull elements are derived from two sources: neural crest cells and mesodermal cells^2^, so there is a developmental interaction between elements of the same cellular origin. Fibroblast growth factors (FGFs), Sonic hedgehog (SHH), WNT signaling pathway, and bone morphogenetic proteins (BMPs) are required for the morphogenesis of the facial cranium derived from neural crest cells^3^. The expression of *Fgf8* in the facial epithelium in mammals and birds, for example, is involved in the morphogenesis of multiple bones in the maxilla^4,5^, so there is a genetic relationship between these tissue morphologies. Functional interactions also include the coordination between the upper and lower jaw during biting and other activities. The result of the interactions by these complex biological processes can be detected as morphological integration and modularity in a given structure. Therefore, the studies of morphological integration and modularity can provide the relationships between tissues not superficially but potentially and intrinsically, and help to understand the factors that constrained or promoted morphological evolution and the evolvability of the organismal form^1^. Although the fundamental idea of modularity in biology has existed since 1958^6^, morphological modularity is a field that has received much attention in recent years^1^.

Among the body tissues of vertebrates, the skull morphology is particularly complex, and its complexity is related to the various functions in protecting the brain and sensory organs and playing roles in feeding and respiration. For this reason, the skull has received particular attention in studies of morphological modularity^7^. The most commonly used method herein has been geometric morphometrics^7^. On the other hand, network analysis is employed to investigate the modularity of head structures in more recent years^8–14^. Conventionally used morphometric methods focus on the covariation of size and shape of skeletal parts of interest. In contrast, network analysis is a method that focuses on the interaction between individual bones, which is of interest because it can provide information about the potential function and complement the traditional morphometric approach^7,15,16^. For example, the geometric morphometrics can only be analyzed from a single side in conventional 2D geometric morphometrics. Additionally, the analysis cannot include non-homologous bones in all specimens as the bones of interest must share a common landmark. Network analysis can overcome the limitations of geometric morphometrics.

Lizards (Lacertilia) belong to Squamata, alongside with snakes, and are the largest group of living reptiles, containing over 7000 species^17^. The lizard skull morphology is extremely diverse. For instance, the group ranges in cranial architecture from amphikinetic skulls in varanids and geckos to the heavily ossified skulls of fossorial taxa^18,19^. Therefore, it is essential to understand the factors that constrain and promote lizard skull diversity as well as its evolution. However, previous studies on the morphological modularity and integration of lizard skulls are limited^20–24^, and network analysis has never been used.

One hypothesis of skull modularity in lizards is a division pattern in the cellular origin of the skull (neural crest composing the face and mesoderm comprising the neurocranium). This is one of the hypotheses supported in the *Anolis* lizards using geometric morphometrics^21^. It is also expected that in highly kinetic cranial species, there is a high degree of functional integration at kinetic joints or that the bony units that work during cranial kinesis are highly modularity.

Here, we utilize Anatomical Network Analysis^15,16^ to compare the integration and modularity of skulls across lizards to understand the evolution of their skull morphology and the factors that control it. Using network analysis, which is almost unprecedented to date, this is an important study on the macroevolution of modularity in lizards skull.

## Results

### Modularity

**Figure 1:**
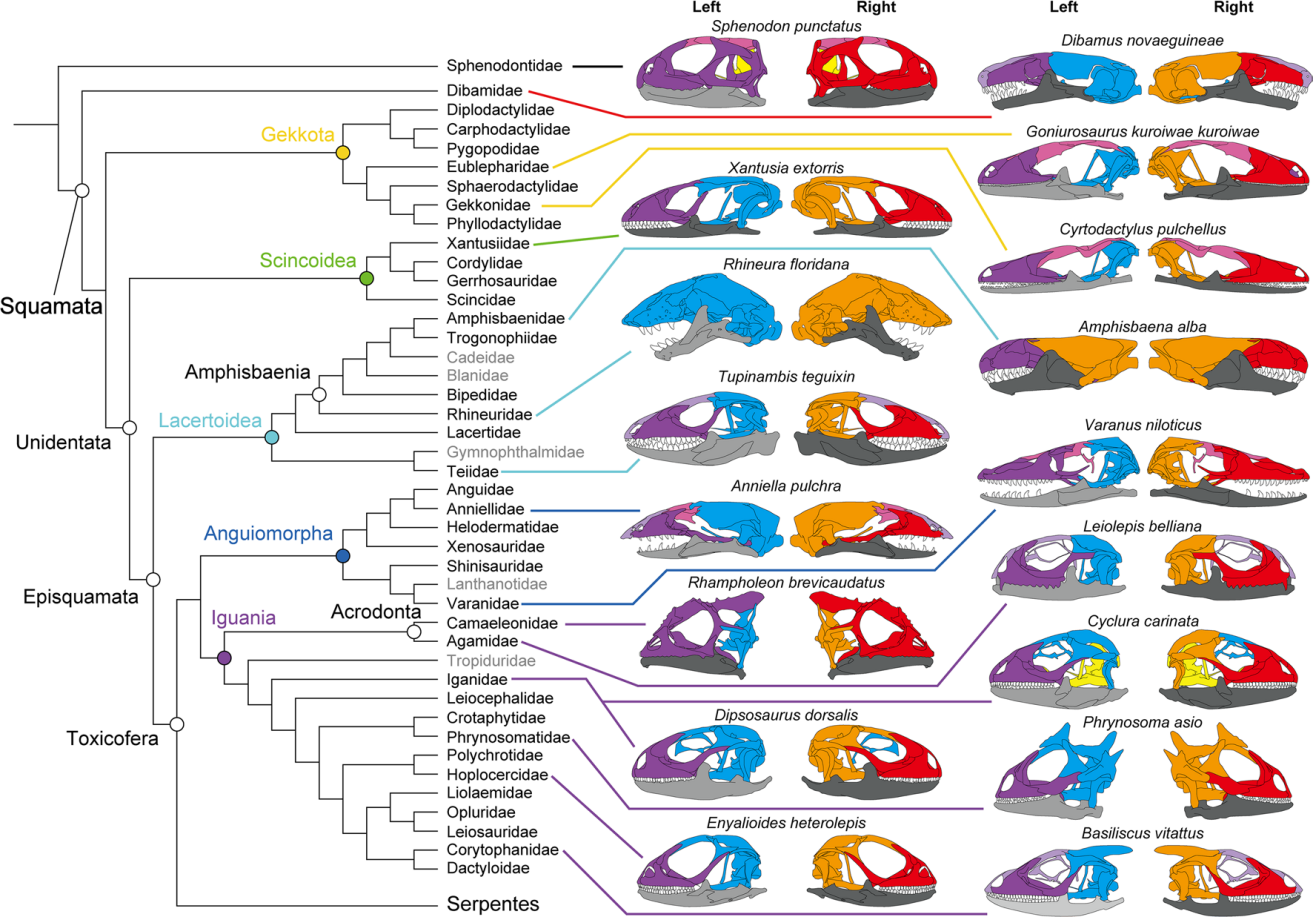
Distribution of skull modules in tuatara and selected lizards from left and right side with the phylogenetic tree. The phylogenetic tree on the left is based on the molecular phylogenetic information in Pyron et al.^49^. Families not covered in this study are grayed out.

The network modules of all species analyzed are shown in Supplementary data 3 file. Despite the morphological diversity of lizard skulls, lizards generally possess separate left and right preorbital (purple and red), postorbital (blue and orange), and mandibular modules (light and dark gray) (Fig. 1). Nevertheless, in some taxa, the snout (light purple), including the premaxilla, nasal and frontal (e.g., *Basiliscus vitattus*, *Draco volans*, *Tupinambis teguixin*), or the braincase elements (yellow) (e.g., *Anolis cristatellus*, *Cyclura carinata*, *Elgaria panamintina*) form a single module (Supplementary data 3 file; Supplementary Figs. 6, 7, 13, 19, 20, and 52). Furthermore, in Gekkotans, the frontals, parietals, and postorbitals form a skull roof module (pink), while in other taxa, the nasals are included in (e.g., *Varanus niloticus*) or the parietals are excluded (e.g., *Anniella pulchra*) from the skull roof module. The parietals are integrated into the preorbital module in Chamaeleonids, whereas iguanians with ornamentation similar to chamaeleonids (*Phrynosom asio*, *Basiliscus vitattus*) have their parietals integrated into the postorbital module. *Rhineura floridana* exhibits a unique pattern in which all the cranium bones are integrated into a single module on each side. In two species of geckos, *Coleonyx variegatus* and *Oedura tryoni*, the pterygoid forms a separate module with the epipterygoid (see Supplementary data 3 file; Supplementary Figs. 10 and 32).

The boundaries between modules in the dorsal and ventral regions are not constant, and the modules, including the parietal and pterygoid, differ from species to species. Except for *Heloderma horridum*, *Brookesia brygooi*, *Rhampholeon brevicaudatus*, and *Oplurus cyclurus*, the lateral boundaries are almost constant in the jugal-postorbital (postorbitofrontal).

The skull of the tuatara differs significantly from lizards and shows the preorbital module containing the temporal bones, a braincase module, and left and right mandibular modules. Interestingly, the jugal of the tuatara is highly integrated with the postorbital (in the dendrogram, the jugal and postorbital are adjacent to each other (see Supplementary data 3 file; Supplementary Fig. 1)). Compared to tuatara, the jugal and postorbital bones of lizards are in separate modules (Fig. 1; see Supplementary data 3 file; Supplementary Figs. 2–58).

### Multivariate analyses of network parameters.

The PC 1 and PC 2 of the network parameters together account for more than 65% of the total variation (see Supplementary data 4 file; worksheet 3). The PC 1 explains most of the parameters except for H. Negative PC 1 values relate to greater N, K, L, Q-modules, S-modules, and Q_max_, and positive values relate to greater D and C. Amphisbaenia, a fossorial taxon with greater D and less N, K, and L, exhibits larger PC 1 scores. However, within Amphisbaenia, *Rhineura floridana* (C = 0.5418546) and *Bipes biporus* (C = 0.4955357) are characterized by greater integration, which differ from *Amphisbaena alba* (C = 0.3447368) and *Trogonophis wiegmanni* (C = 0.3429654), and results in larger PC 1 score. Lacertidae and Teiidae (the basal Lacertoidea) are intermediate and separated from Amphisbaenia (the derived Lacertoidea) along with PC 1. The PC 1 score for the tuatara was 0.5331 and intermediate.

Negative PC 2 values relate to the greater C and H and positive values relate to greater K. Most gekkotans with specialized skulls without postorbital bars and upper temporal bars are plotted on the negative side of PC 2 due to the small value of K. The Mann–Whitney U test strongly supports that Gekkota (n = 14) and other lizards (n = 44) differ from each other in the present multivariate analyses (Table 1). In other words, only K is lower in the skull of Gekkota than in that of other lizards, and the reduction in connectivity due to the absence of postorbital bars and upper temporal bars in Gekkota does not seem to affect other parameters. Notably, the PC 2 score of the tuatara is the greatest (2.6805), which is due to relatively low C (0.3544974) and the lowest H (0.2763419). This result indicates that lizards evolved skulls that were highly integrated and had greater anisomerism than the tuatara.

**Table 1:** Comparison of network parameters and principal components scores using the Mann–Whitney U test. Values with significant differences are shown in bold.

	Fossorial (n = 6) vs. non-fossorial (n = 52)		Gekkota (n = 14) vs. non-Gekkota (n = 44)	
	z-value	p-value	z-value	p-value
N	**3.30537**	**0.00095**	1.90387	0.05693
K	**2.80996**	**0.00496**	**3.18151**	**0.00147**
D	**3.37071**	**0.00075**	0.89049	0.37320
C	0.17873	0.85820	1.05515	0.29140
L	**2.80854**	**0.00498**	1.23753	0.21590
H	0.66385	0.50680	0.98124	0.32650
S-modules	1.70766	0.08770	0.42779	0.66880
Q-modules	**2.84809**	**0.00440**	1.91221	0.05585
Q_max_	**3.24258**	**0.00119**	0.62052	0.53490
PC 1	**3.24258**	**0.00119**	0.96428	0.33490
PC 2	0.20426	0.83820	**2.84763**	**0.00441**
pPC 1	**3.34471**	**0.00082**	1.01878	0.3083
pPC 2	0.30639	0.75930	**3.27301**	**0.00106**

**Figure 2:**
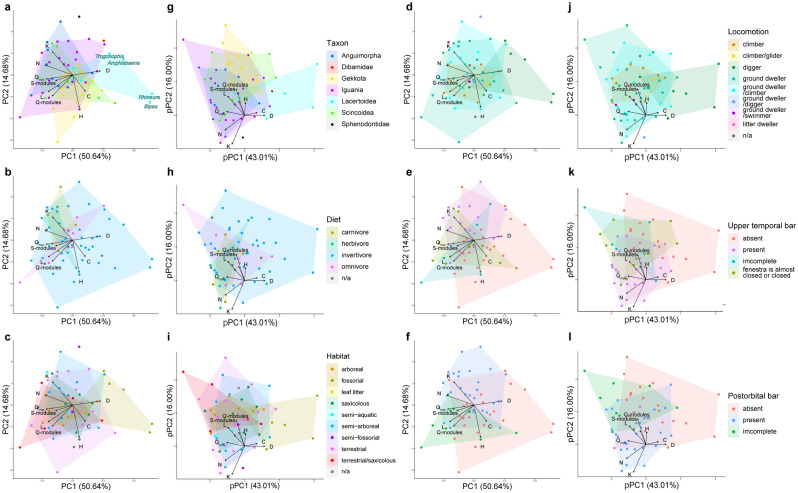
Results of principal component analysis (PCA) and phylogenetic principal component analysis (pPCA). PCA plots colored by (**a**) taxon, (**b**) diet, (**c**) habitat, (**d**) locomotion, (**e**) presence of upper temporal bar and (**f**) presence of postorbital bar. pPCA plots colored by (**g**) taxon, (**h**) diet, (**i**) habitat, (**j**) locomotion, (**k**) presence of upper temporal bar and (**l**) presence of postorbital bar.

The pPC 1 and pPC 2 of the network parameters together account for about 60% of the total variation (see Supplementary data 4 file). The distributions of pPC 1 and pPC 2 are essentially unchanged compared to the PCA results, which indicates that there is not much phylogenetic signal in network parameters (Fig. 2). However, the position of the most phylogenetically basal and fossorial species, *Dibamus novaeguineae*, shifts its placement compared to the PCA results, becoming more similar to phylogenetically distant yet alike fossorial Amphisbaenia (Fig. 2a,g).

In each ecological category, the network parameters did not differ by diet, but they did differ by habitats and locomotion. Analyses on habitats and locomotion (Fig. 2c,d) result in greater PC 1 scorers for fossorial and digging lizards due to their lower N, K, L, Q-Modules, Q_max_, and higher D than those of other species. Thus, the skulls of fossorial (digger) species are morphologically more complex and have evolved higher functional efficiency and morphological complexity than those of other species. The Mann–Whitney U test supports that fossorial and digger lizards (n = 6) differ from other species (n = 52) (Table 1).

**Figure 3:**
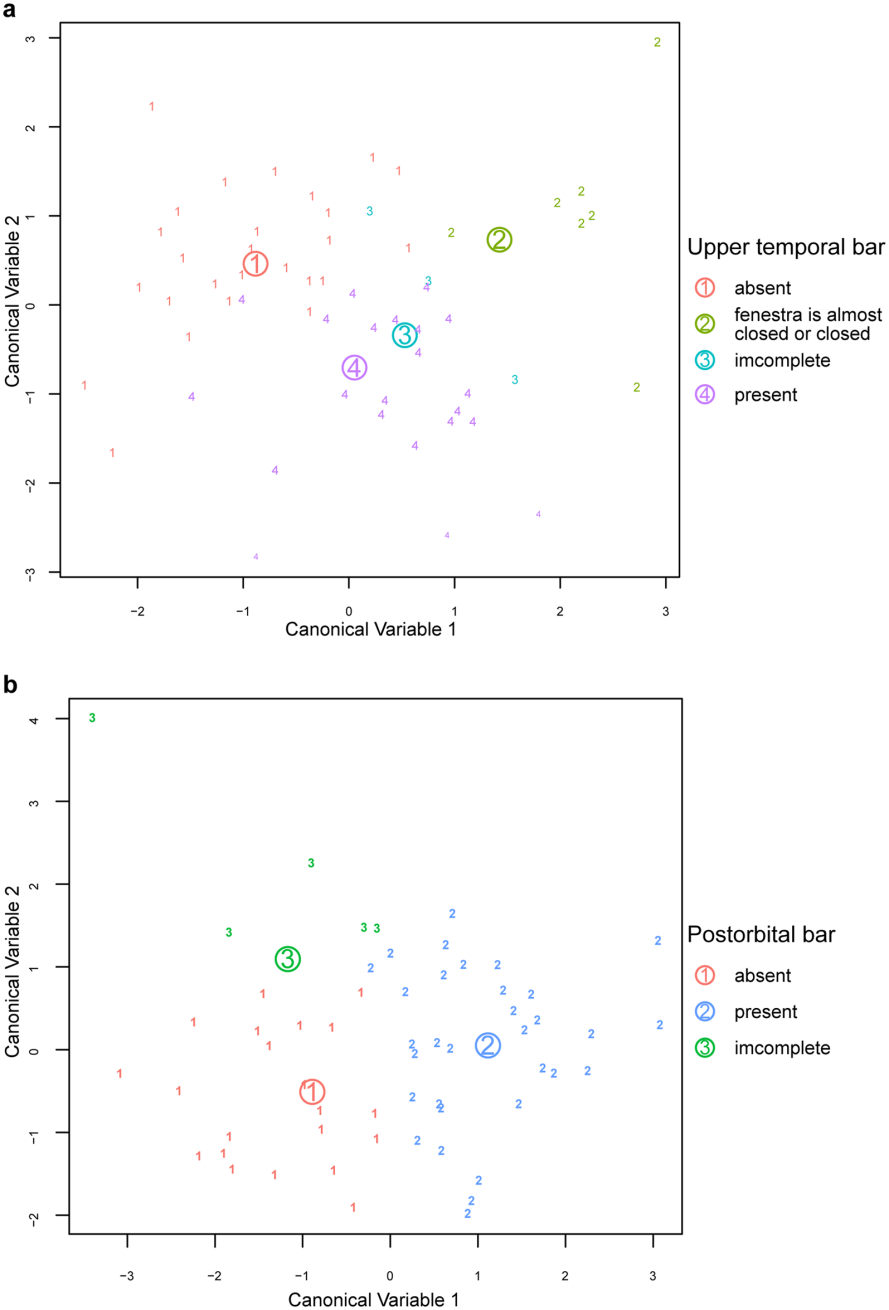
Results of flexible discriminant analysis (FDA). (**a**) On the presence of the upper temporal bar. (**b**) On the presence of the postorbital bar.

In the morphological categories, the presence or absence of the upper temporal bars does not appear to be explained in parameters. In the PCA plots, the groups with the upper temporal bars cluster, while those without the upper temporal bars are scattered (Fig. 2e,k). The flexible discriminant analysis (FDA) on upper temporal bars shows a misclassification error rate of 34.48%, which indicates that the presence of upper temporal bars has no effect on the parameters (Fig. 3a). On the other hand, the presence or absence of postorbital bars does not have a strong association with the differences in the parameters, where the distribution of each group overlaps in the PCA plots (Fig. 2f,l). Nonetheless, the group without postorbital bars tends to score greater PC 1 values. In contrast, the group with postorbital bars scores lower PC 1 and greater PC 2 values, while the group with an incomplete postorbital bar tends to scores lower PC 1 and PC 2 values. The FDA on postorbital bars indicates a misclassification error rate of 20.69%, suggesting that the presence of postorbital bars has a weak effect on the parameters (Fig. 3b).

## Discussion

### Symmetry and asymmetry of modular pattern

In AnNA, asymmetric modularity can be detected in the left and right sides of the skulls even in anatomically symmetric structures. Such examples have been detected in the skulls, muscles, and limbs of various taxa^11–14^. Since the network models and cluster analyses do not distinguish left and right, even if bone connections are coded identically on the left and right, it is expected to result in asymmetric modular structures. A previous study mentions that asymmetrical results of network analysis might be an artifact^14^. In this study, nearly symmetric modules are obtained by virtually dividing an unpaired bone in the median sagittally into the left and right elements and coding it as a pair of bones.

### Evolutionary directions of lizard skulls in the context of modularity by AnNA

The essential significance of the common modular pattern of lizard skulls found in this study is that the regions of concentration of bony connections are evolutionarily conserved in Lacertilia. The variation in network parameters indicates that the connection patterns are phylogenetically independent and diversified. Although it should be noted that this study differs in that it recognizes unpaired bones as a pair of bones, we compare the parameters identified in previous studies using AnNA^13,43^ with the results of lizards. The range of parameters in lizards is comparable to that of amniotes, although not as comprehensive as that of vertebrates (e.g., C: vertebrates = 0.29–0.63; amniotes = 0.30–0.47; Lacertilia = 0.32–0.54). In terms of patterns of connections, the skulls of lizards are highly diversified. These points suggest that the regions of concentrated connections (modularity) are evolutionarily stable, and the connection patterns within the regions are evolutionarily highly variable.

### Factors causing modularity and integration in lizard skulls

The general preorbital and postorbital modular patterns of lizards appear to be largely phylogenetically influenced. However, a comparison of the PCA and pPCA results for the network parameters reveals that the distribution of the plots hardly differs, indicating that factors other than phylogeny play a major role in the preorbital and postorbital modular patterns.

**Figure 4:**
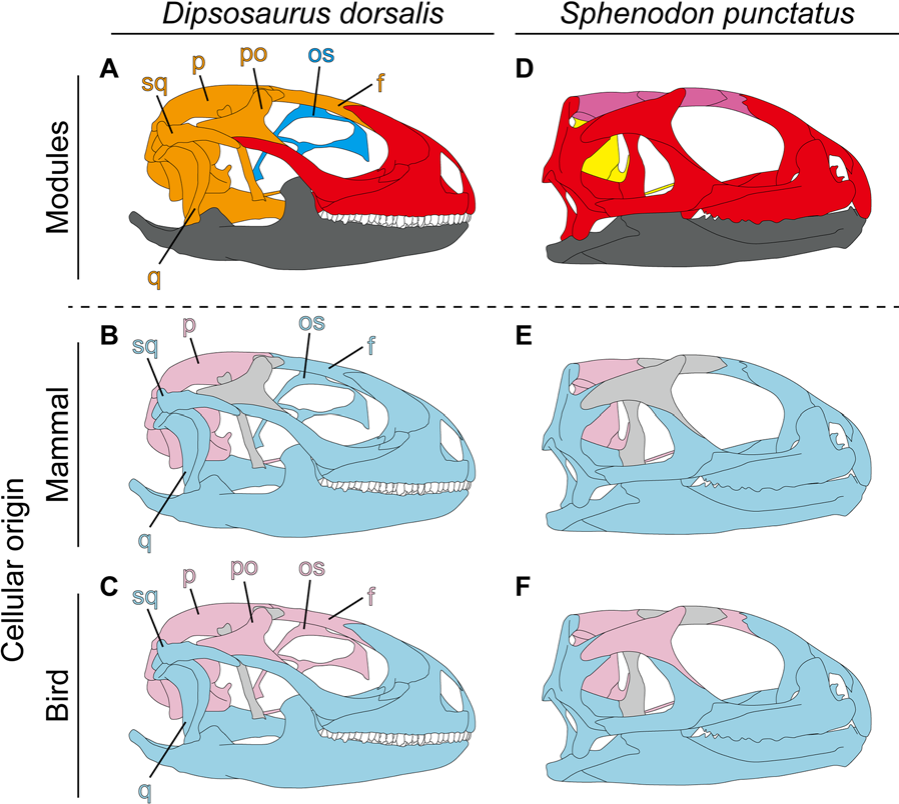
Comparison of the skull modules of lizards and tuatara with the cellular origin patterns. Based on Noden and Trainor (2005)^2^, (**A**–**C**) skulls of lizards *(Dipsosaurus dorsalis*) and (**D**–**F**) tuatara are color-coded for mammalian and bird cellular origin patterns (blue, the neural-crest-derived elements; pink, mesoderm-derived elements; gray, non-homologous elements). Abbreviations: f, frontal; os, orbitosphenoid; p, parietal; po, postorbital; q, quadrate; sq, squamosal.

The developmental processes shape the morphological structures of an adult. In previous studies on the modularity of the lizard skulls^21,23^, one of the developmental factors, cellular origin (neural crest and mesoderm), is adopted from patterns found in mammals. However, skeletal homology between lizards and mammals is not fully appreciated, and it is unknown if the cellular origin patterns of mammals are applicable to lizards^25^. In this regard, we compare the amniote cellular origin patterns of both birds and mammals with the modular patterns of lizards and tuatara, based on Noden and Trainor (2005)^2^. Cellular origins of the temporal elements (quadrates, squamosals, pterygoids) are especially inconsistent with the modularity in lizards (Fig. 4A–C). In other words, modularity in the lizard skull is probably caused by factors other than morphogenesis. On the other hand, the modular pattern of the tuatara is almost identical to the amniote cellular origin patterns: mesoderm of neurocranial and skull roof bones and neural crest of facial and mandibular bones (Fig. 4D–F). Therefore, it is likely that interactions between skull elements during development strongly influence skull morphology in the tuatara.

**Figure 5:**
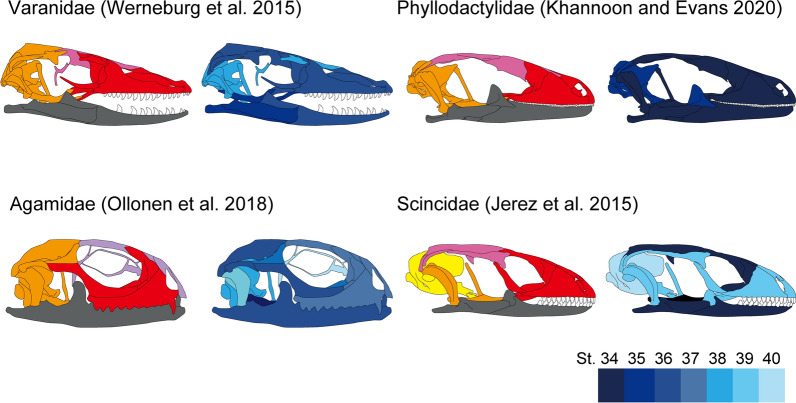
Comparison of ossification sequence and modularity of lizard skulls. These figures are based on comparing the ossification sequence by Khannoon & Evans^26^ in the developmental stages (blue values) according to Dufaure and Hubert^51^. The ossification sequence patterns with the modules of the same family are compared, respectively^26,52–54^.

Another possible factor shaping the modularity of lizard skulls is the difference in ossification sequence patterns. It appears that the ossification sequences of lizard skulls by Khannoon & Evans^26^ and the modular pattern of lizards in this study lacks any correlations (Fig. 5). Therefore, it is reasonable to assume that the modularity of lizards reflects factors other than ontogenetic development.

The general modularity in lizards may reflect functional factors. The modularity would correspond to the functional requirements of the preorbital region of the snout and upper jaw associated with feeding and olfaction, as well as the postorbital region of the braincase and temporal elements associated with brain protection, jaw muscle attachments, and adductor chamber. Independent covariation patterns of anterior and posterior regions in the dorsal skull shape shown in Dactyloids^21^ and Lacertids^23^ are consistent with present results of a general pre-postorbital modular division. It is concluded that both of their covariation patterns reflect functional demands, which supports present hypothesis described above. Because the fundamental functional requirements do not vary significantly among all taxon, the modularity is likely phylogenetically common to some extent.

In contrast to the tuatara and most non-squamate diapsids, lizards lack the lower temporal bar in their skulls. Rieppel & Gronowski^27^ proposes that the absence of the quadratojugal and the loss of the lower temporal bar results from the expansion of the external adductor muscle. This observation is consistent with the modular patterns of the lizards reflecting functional factors of feeding, olfaction, muscle attachments, and brain protection more strongly than that of the tuatara. However, it should be noted that the features once considered plesiomorphic, including the lower temporal bar, can be derived or secondarily acquired in *Sphenodon*^28–32^. Whether the modular pattern of the lizards is ancestral or derived remains to be tested as a matter for consideration.

We found not only general modularity but also variation in modular patterns between lizard taxa. These include the dorsoventral module boundaries, separation of the rostral, skull roof, and braincase modules, and unique modular patterns particularly in chameleons and *Rhineura floridana*. Again, the covariation patterns of the lizard skulls investigated in previous studies^21–23^ are consistent with the general modularity in this study. This also means that the modularity results for lizard skulls in this study may indicate a yet unknown intraspecific covariance pattern in most lizard taxon.

### Loss of postorbital bar

The postorbital bar is composed of the jugal-postorbitofrontal (or postorbital and postfrontal) contact in the tuatara and many lizards. In contrast, Gekkota, Varanidae, Dibamidae, Amphisbaenia, Anguidae, and Anniellidae lack the postorbital bar. The bony jugal-postorbital connection is also lost in Scindoidea and Anguiomorpha and probably articulated by soft tissue. This corresponds to the widely-common jugal-postorbital (postorbitofrontal) lateral module boundaries in other lizards with a postorbital bar, while dorsal and ventral module boundaries vary. In tuatara however, the jugal-postorbital contact is highly integrated as demonstrated by these bones being placed on adjacent branches in the network dendrogram (see Supplementary data 3 file; Supplementary Fig. 1). It is possible that the low integration of jugal-postorbital was maintained from the common ancestor of Squamata and that selection pressure caused the loss of the contact in each lineage.

The module patterns of the lizard skulls are unaffected by the presence or absence of an upper temporal bar. In contrast, the FDA results indicate that network parameters vary depending on the presence or absence of postorbital bars, which are absent in geckos and fossorial species. Geckos may have reduced connections around the postorbital bone due to structural constraints caused by the enlargement of the eye in the common ancestor^33^, and this is manifested in reduced K. Fully fossorial species tend to degenerate their limbs and use their heads to burrow^34–37^; therefore, their heads are subject to large external forces. A solid skull for resistance to such forces could be provided by bone fusion (lower N) and increased connection (greater D and lower L) by the enlargement of the contact surfaces between the bones. In other words, each of these different factors, not the presence or absence of a postorbital bar, is responsible for the differences in the parameters.

### Cranial kinesis and modularity

There seems to be little relevance of cranial kinesis to modularity and network parameters. In taxa with well-developed kinesis (e.g., geckos and varanids), little correspondence exists between the boundaries of modules in which their integration is low in the metakinetic (parietal-supraoccipital), mesokinetic (frontal-parietal), hypokinetic (palatine-pterygoid) axes. Additionally, while Werneburg et al.^13^ investigates the cranial kinesis in *Tyrannosaurus rex* and extant amniotes and argues that species with potential kinesis in their skulls have a larger N and lower D, we do not observe the trend in present dataset. For instance, geckos, known to have well-developed kinesis, did not differ significantly from other taxa except in parameters for K (Table 1). The lizard taxa with known degrees of cranial kinesis are indeed limited^19,32,38,39^. However, the degree of cranial kinesis may not be inferred simply by network parameters and modular patterns.

## Methods

### Sampling

Samples included 57 skulls belonging to 57 species of 38 families in the extant Lacertilia (lizards). Because extant Lacertilia consists of 43 families, the samples in this study nearly cover the entire clade. We also included the species on most cranial kinetic conditions in our sample (see Supplementary data 1 file). In addition, we examined tuatara *Sphenodon puctatus* (Lepidosauria) as an outgroup. All skulls come from adult specimens. Computed tomography (CT) data available at Morphosource (https://www.morphosource.org/) was used for analyses of 45 lacertilian species and *Sphenodon* (see Supplementary data 1 file). For other 12 specimens, CT images were acquired for the skulls in collections of the National Museum of Nature and Science, Tokyo, or Institute of Dinosaur Research, Fukui Prefectural University (see Supplementary data 1 file). Those CT data were collected by Latheta LCT-200 (Hitachi, Ltd.) or FF35 CT Metrology (Yxlon).

### Anatomical Network Analysis (AnNA)

Following the previous studies^13,14^, AnNA was conducted to verify the modularity of the lizard skulls. Unweighted and undirected network matrixes for AnNA of the lizards and tuatara skulls were prepared according to the following method. If two or more skull elements were fused without visible sutures, they were treated as one unit. For a single unpaired bone in the median, we coded it as a pair of bones on the left and right sides by virtually dividing it into two left and right elements sagittally. The presence of contacts between the bones or units was determined by observing the CT images and 3D models on *VGStudio MAX 3.3*. Each contact between two bones or units was coded as "1", and the absence of contact was coded as "0" (see Supplementary data 2 file).

As in bone-to-bone/unit-to-unit contacts, articulations were generally coded as "1". However, lizards are equipped with well-developed kinetic joints such as syndesmosis and synovial joints^39^, and it is necessary to recognize the condition of kinetic joints. If they are joined by soft tissues such as ligaments or cartilage, the bones or units may appear to be separated from each other on CT images and 3D models. Although the morphological information of soft tissues should be used to code the joint condition in the skull, studies on the soft tissues of kinetic joints in lizards are very limited^40^. Therefore, in this study, we uniformly coded "1" for joints if the hard bones were in direct contact with each other.

Following the script of Plateau & Foth (2020)^14^, the data matrix of each sample was analyzed using the software *R-3.6.3*^41^ and the package *igraph*^42^. The analyses determined a number of network parameters for each network^43^, which, in turn, describe the skull anatomy. The number of nodes (N) and connections (K) represent the number of bones and their contacts of each sample, respectively. The density of connections (D) measures the number of existing connections with respect to the maximum possible, thus D is interpreted as a proxy of morphological complexity. The mean clustering coefficient (C) measures the average of the sum of connections between all neighbors of each node with respect to the maximum possible, thus C is interpreted as a proxy of anatomical integration. The mean shortest path length (L) measures the average minimum distance between all nodes, thus L is interpreted as a proxy of functional efficiency. The heterogeneity of connectivity (H) is the standard deviation divided by the mean number of connections of all nodes in the network. Thus H is interpreted as a proxy of anisomerism. As the number of bones reduces, each bone element becomes more complex to compensate for it^43,44^. This compensation is called anisomerism, and the heterogeneity of bone connections indicates that each bone element becomes more specialized. Modules of the anatomical networks were identified by the hierarchical clustering of the generalized topological overlap similarity matrix among nodes (GTOM). Here, as in Plateau and Foth (2020)^14^, Ward.D2, which minimizes the increase in the sum of squared distances between OTUs in clusters, was used to minimize variance^45,46^. The optimization function modularity Q^47^ determined Q-modules and Q_max_. The function evaluates the partitions by cutting the dendrogram so that the proportion of connections in each partition is the highest (by Q_max_) compared to the expected value if the connections were randomly placed. That is, Q-modules means the number of modules determined by this, and Q_max_ means the identified partition quality. The S-modules were estimated by performing a two-sample Wilcoxon rank-sum test on internal and external connections of every module for more biologically meaningful interpretation.

### Multivariate analyses of network parameters

We performed multivariate analyses of the calculated network parameters to evaluate the factors driving morphological evolution in Lacertilia. Principal component analyses (PCA) were performed on the network parameters to compare the distribution of phylogeny, morphological character, and ecology (diet, habitats, and locomotion) in the multivariate data. Ecological and morphological traits of all sampled species are shown in Supplementary data 1 file. The definitions of the ecological traits were adapted from Watanabe et al. (2019)^24^. We also performed phylogenetic principal component analyses (pPCA)^48^ to account for phylogenetic effects on network parameters. The phylogenetic hypothesis of Lacertilia for pPCA were based on Pyron et al.^49^ and *Mesquite*^50^ were employed to select species and create NEXUS data for pPCA (Supplementary data 6). In addition, we conducted the Mann–Whitney U test to see if there are statistically significant differences in parameters, taxonomically or ecologically. Furthermore, we conducted a flexible discriminant analysis (FDA) see if there are statistically significant differences in parameters in morphological traits.

## Supplementary Information

Supplementary Information 1.

Supplementary Information 2.

Supplementary Information 3.

Supplementary Information 4.

Supplementary Information 5.

Supplementary Information 6.

## Data Availability

All data analyzed during this study are included in this published article and its supplementary information files.
