# Supplementary material for: Anatomical network analyses reveal evolutionary integration and modularity in the lizards skull
Source: Sci Rep. 2022 Sep 5;12:14429. doi: 10.1038/s41598-022-18222-8 (PMC9445097; doi:10.1038/s41598-022-18222-8)
Supplement: Supplementary file 3 — Supplementary Information 3. [file 41598_2022_18222_MOESM3_ESM.pdf]

Supplementary data 3. Skull dendrograms of tuatara and lizards.

The branches above the red dashed line are the Q-modules. Cluster with a circle are all S-modules; filled circles in clusters mark the statistical significance of this potential module (white, p-value < 0.05; grey, p-value < 0.01; black, p-value < 0.001).

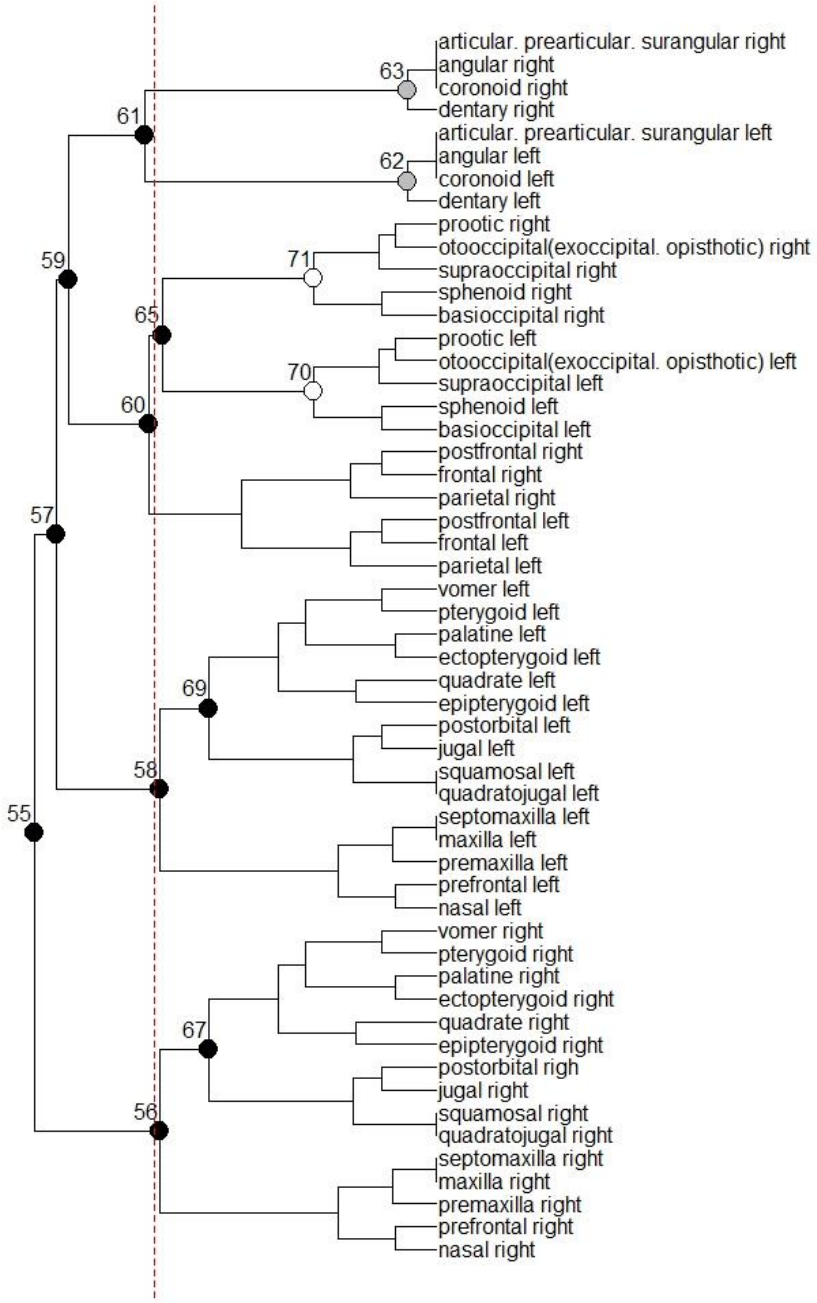

Supplementary Figure 1. The skull network dendrogram of *Sphenodon punctatus*.

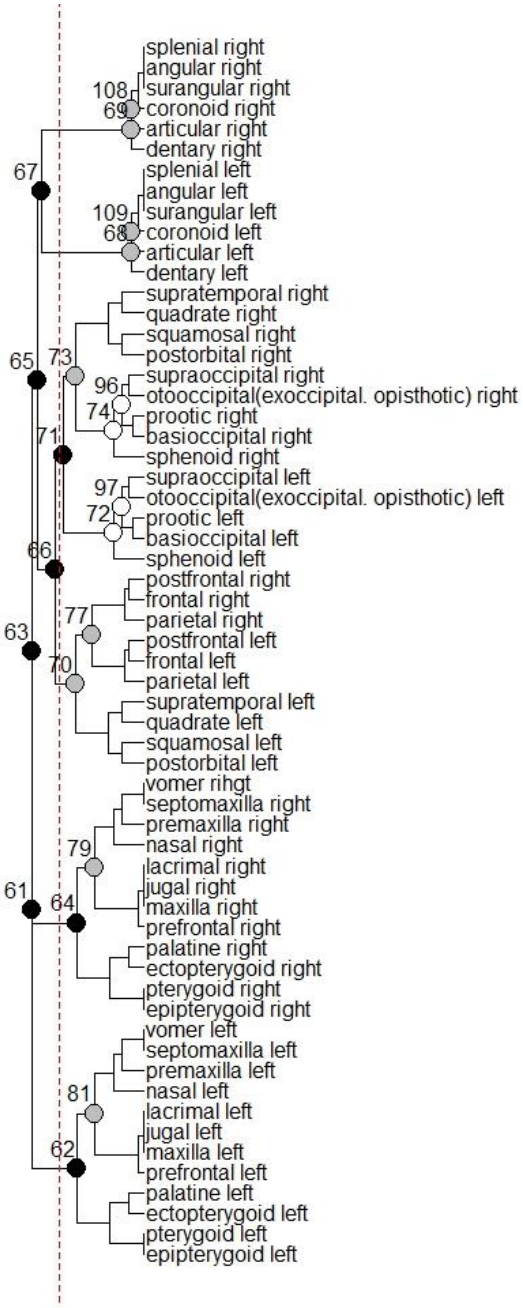

Supplementary Figure 2. The skull network dendrogram of *Abronia ornelasi*.

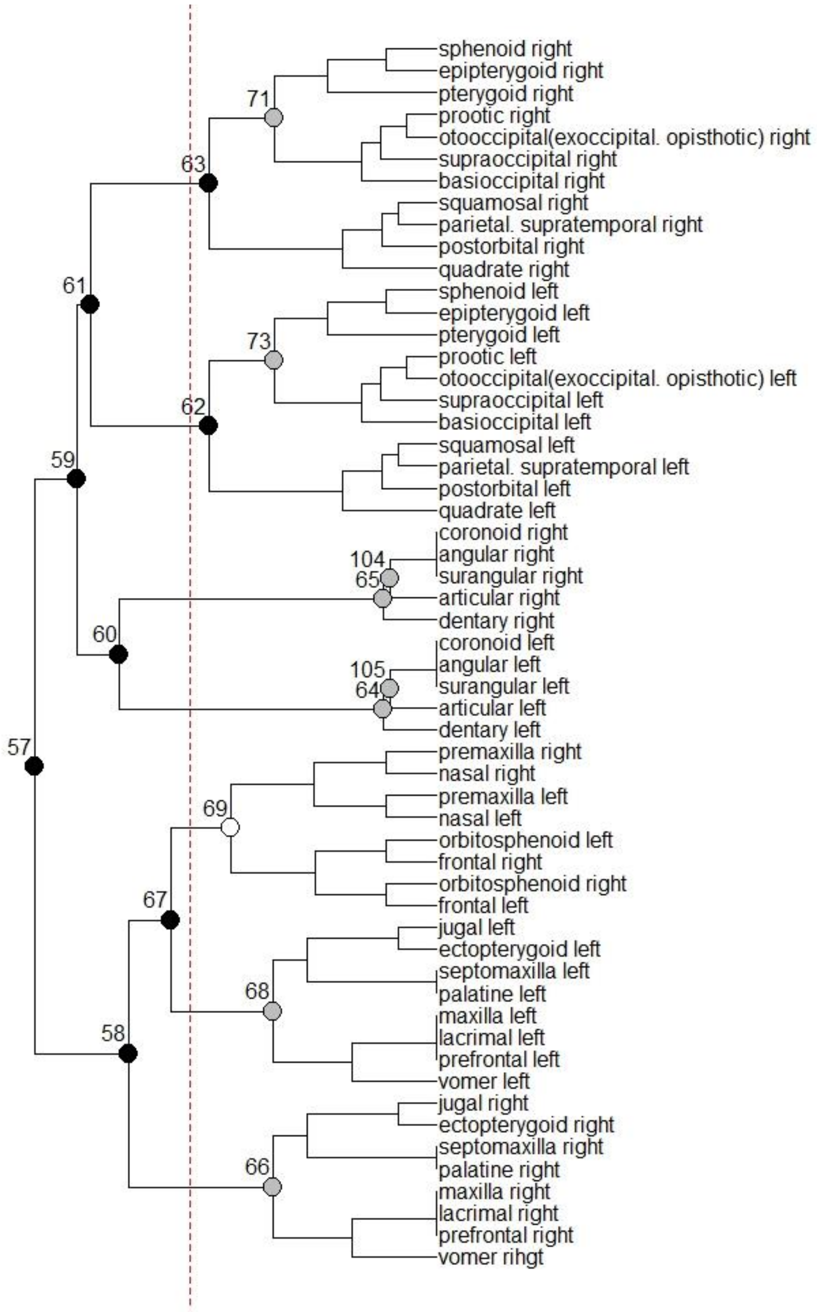

Supplementary Figure 3. The skull network dendrogram of *Agama* sp.

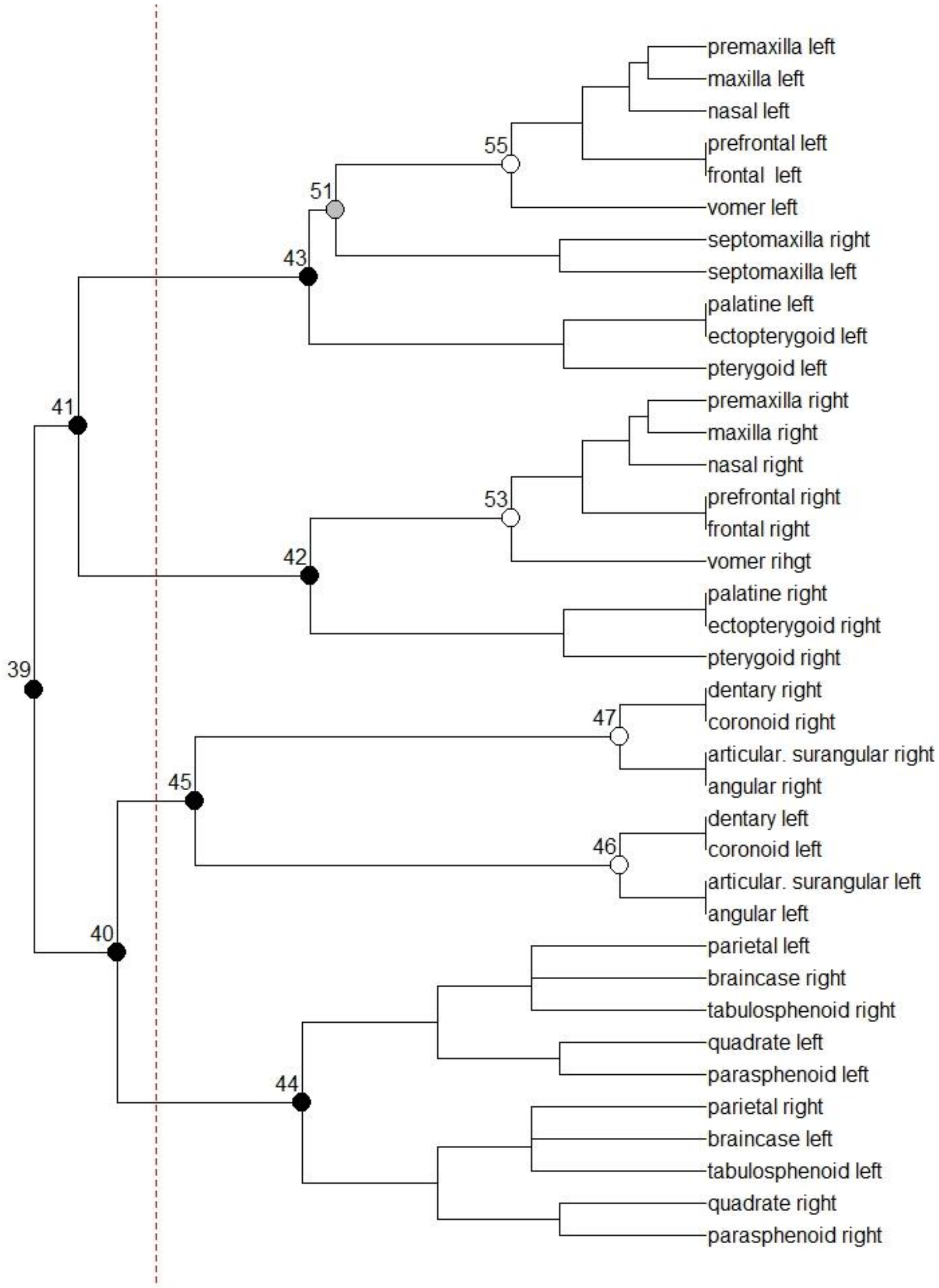

Supplementary Figure 4. The skull network dendrogram of *Amphisbaena alba*.

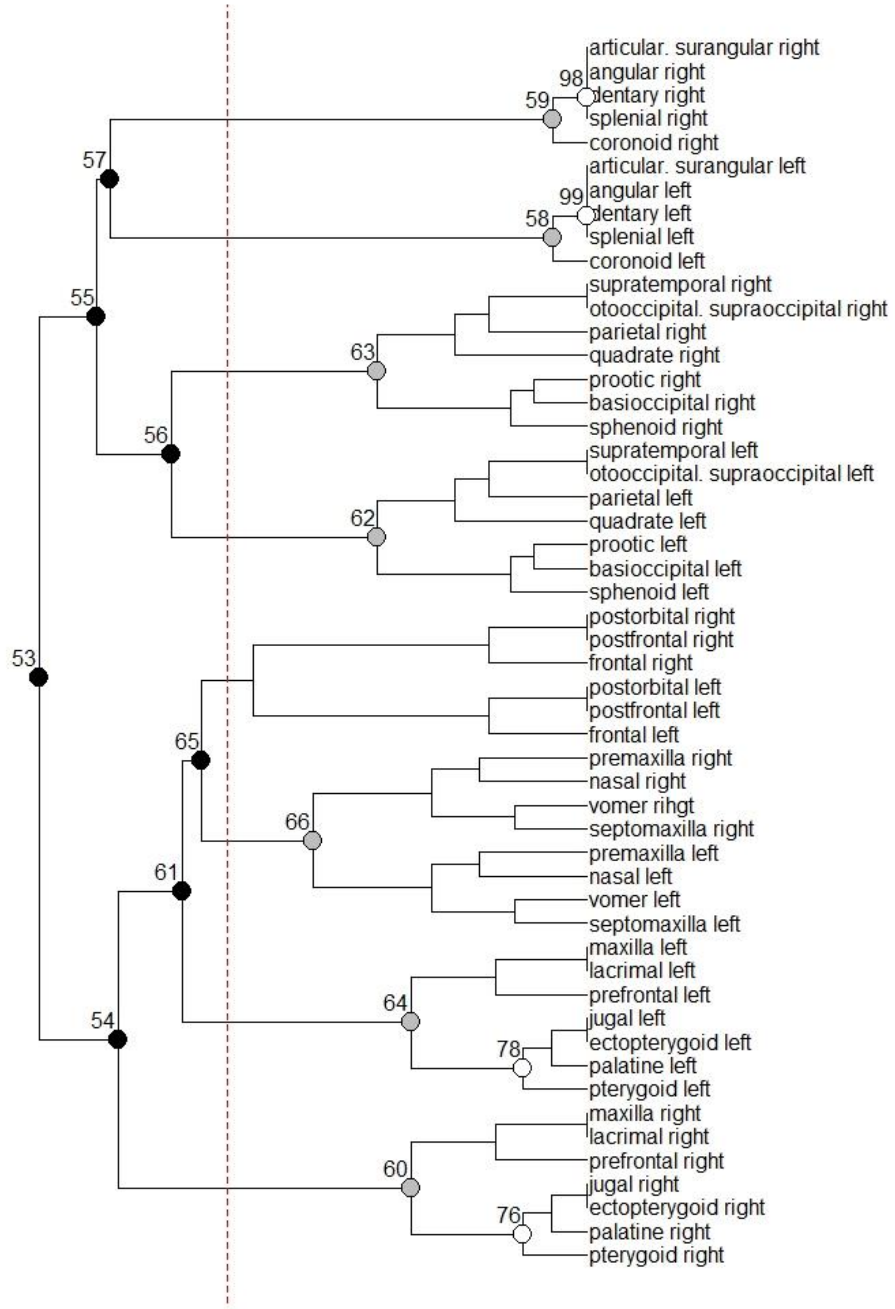

Supplementary Figure 5. The skull network dendrogram of *Anniella pulchra*.

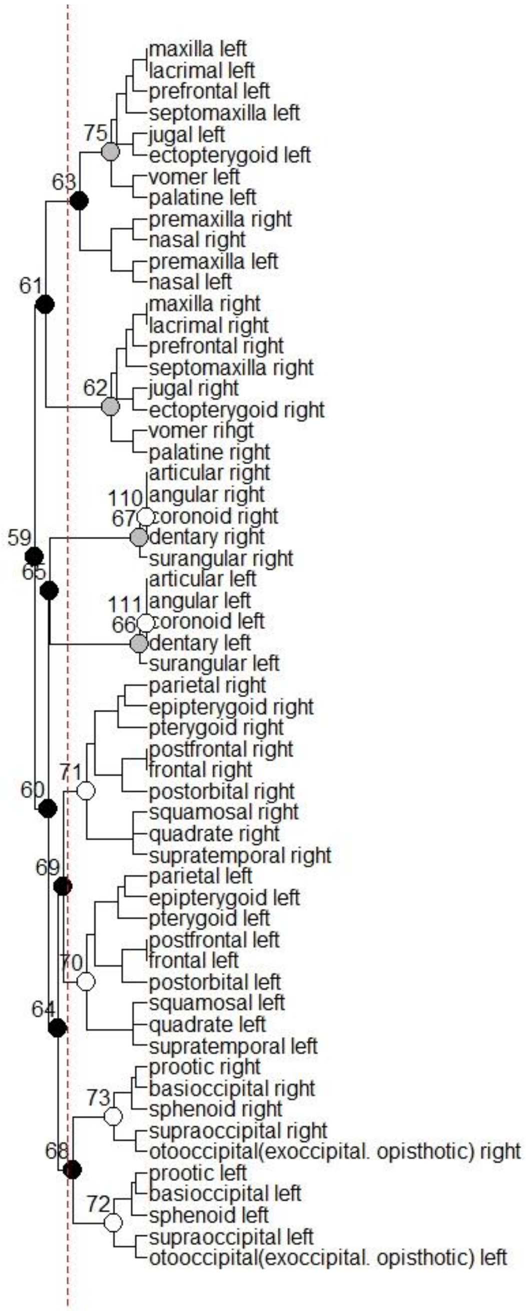

Supplementary Figure 6. The skull network dendrogram of *Anolis cristatellus*.

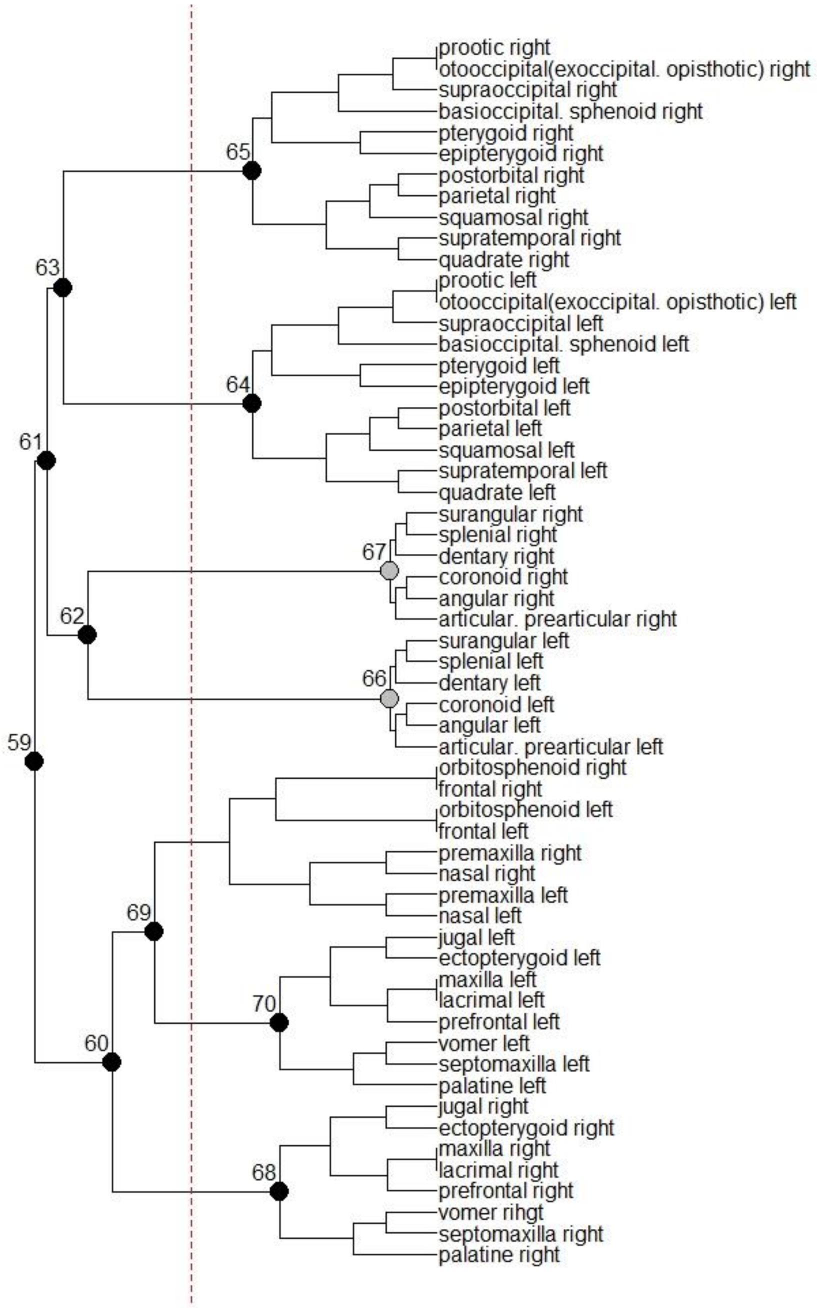

Supplementary Figure 7. The skull network dendrogram of *Basiliscus vitattus*.

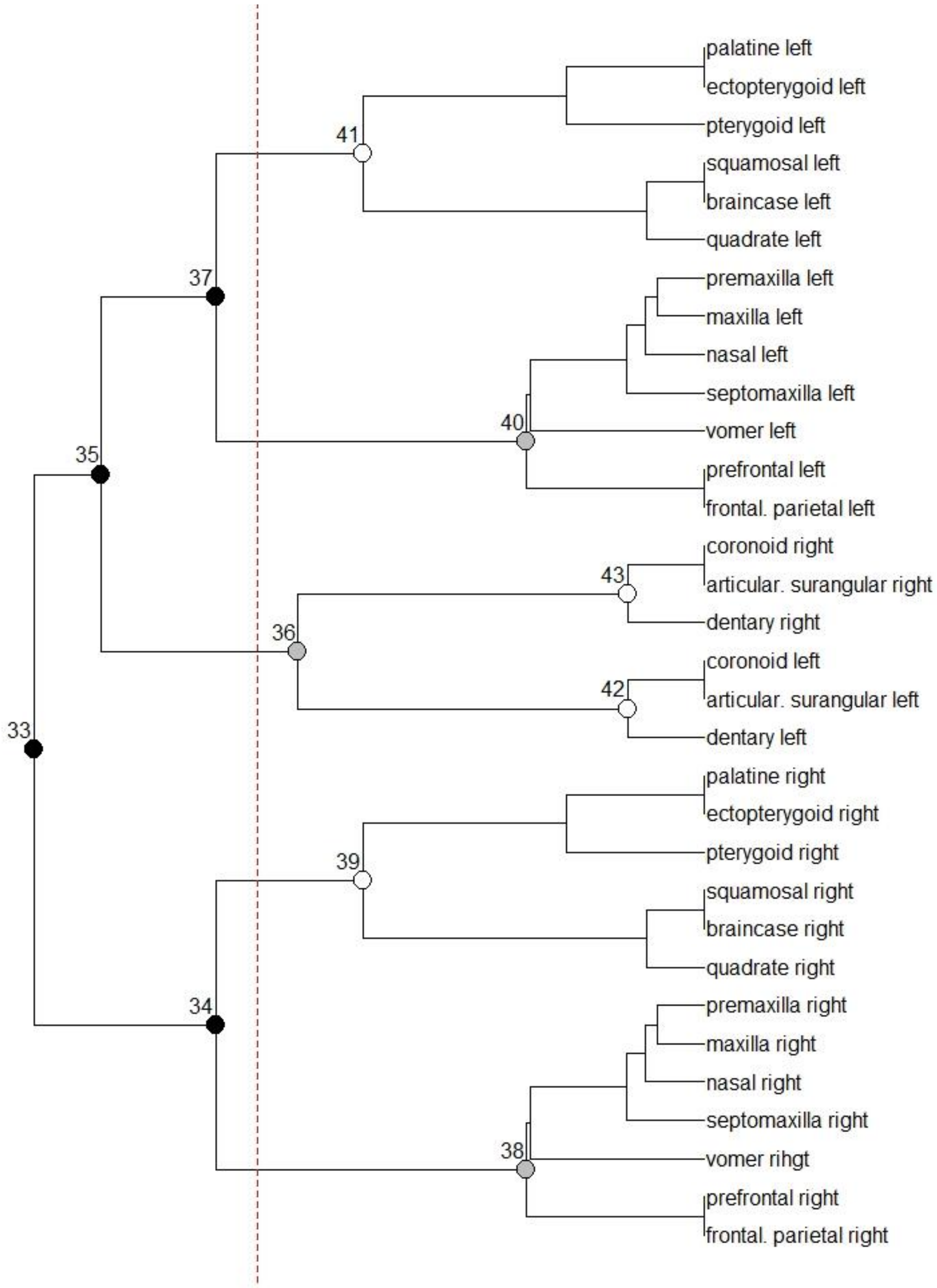

Supplementary Figure 8. The skull network dendrogram of *Bipes biporus*.

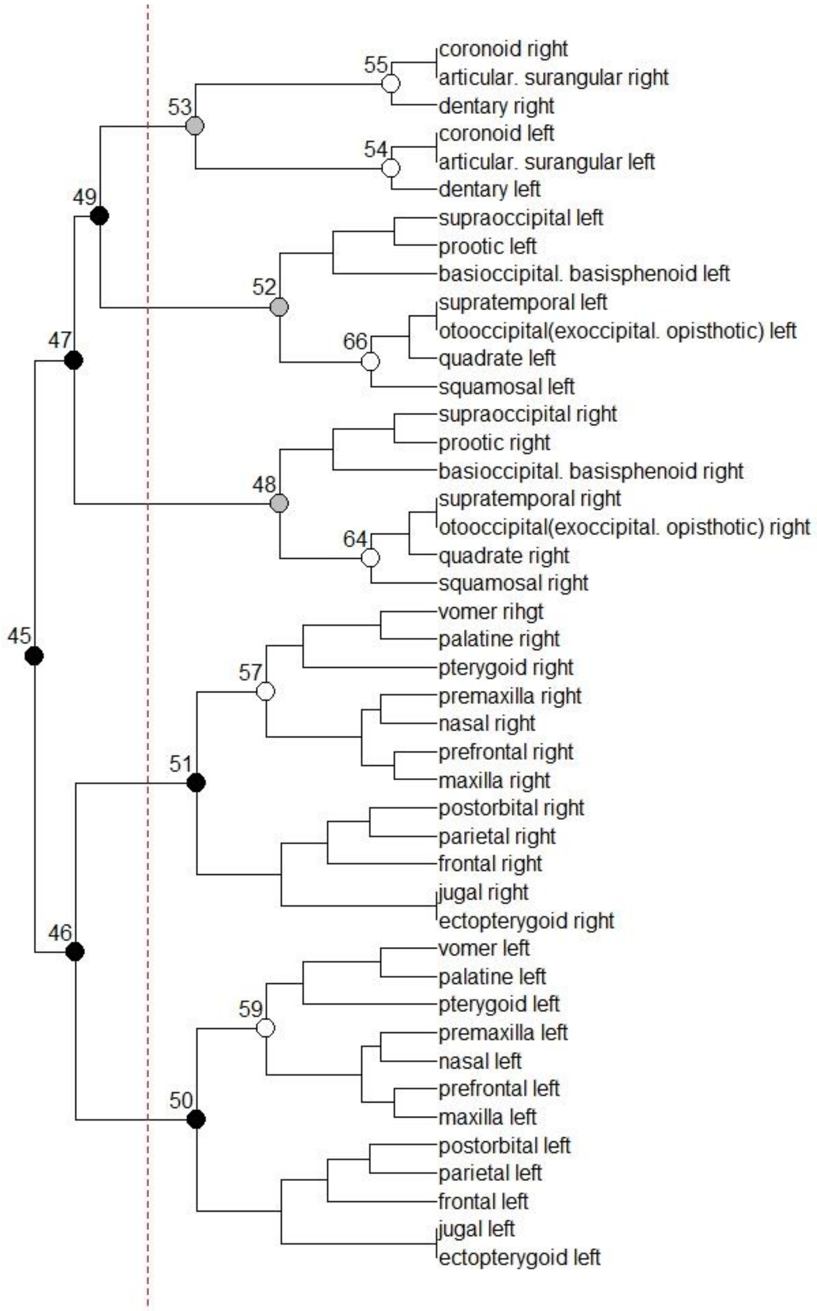

Supplementary Figure 9. The skull network dendrogram of *Brookesia brygooi*.

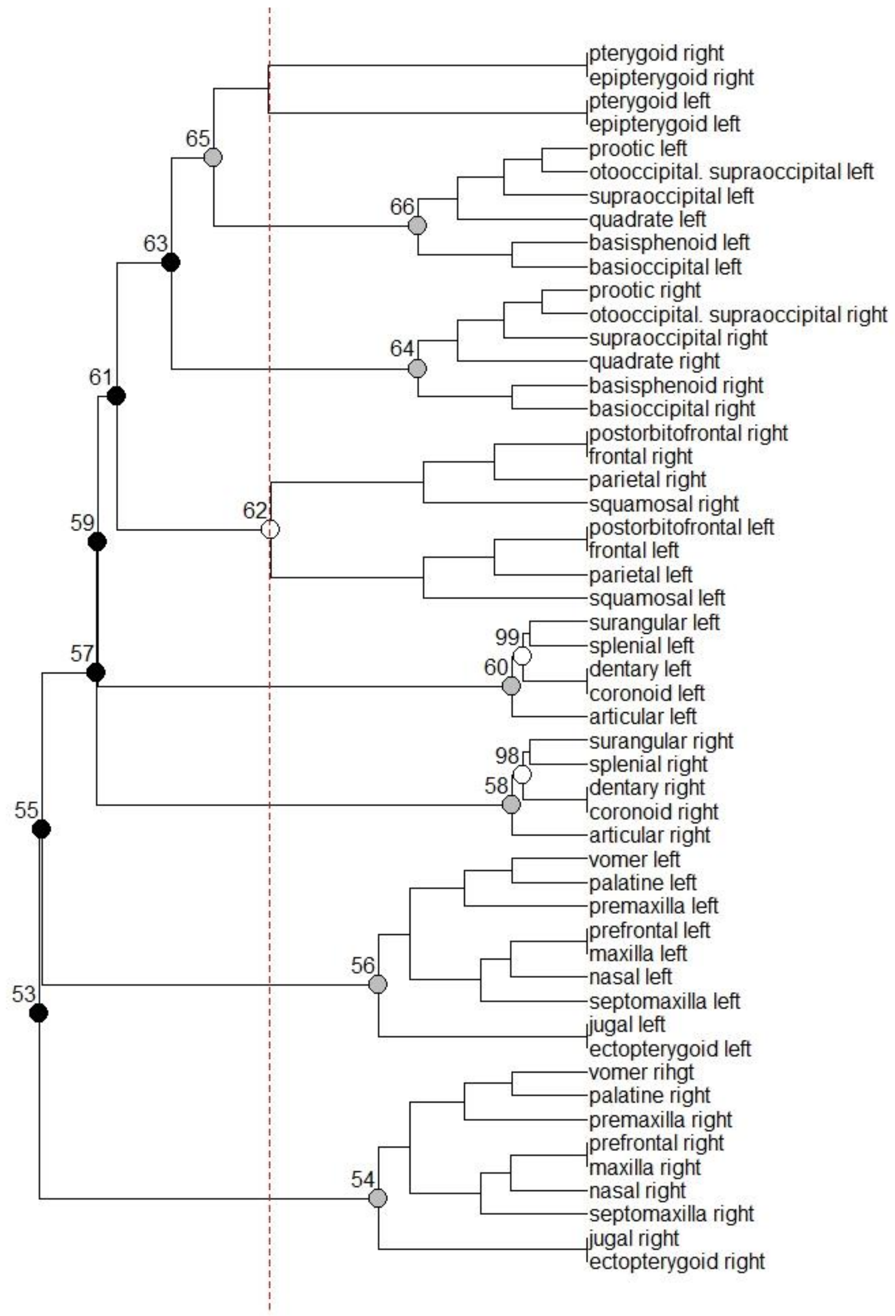

Supplementary Figure 10. The skull network dendrogram of *Coleonyx variegatus*.

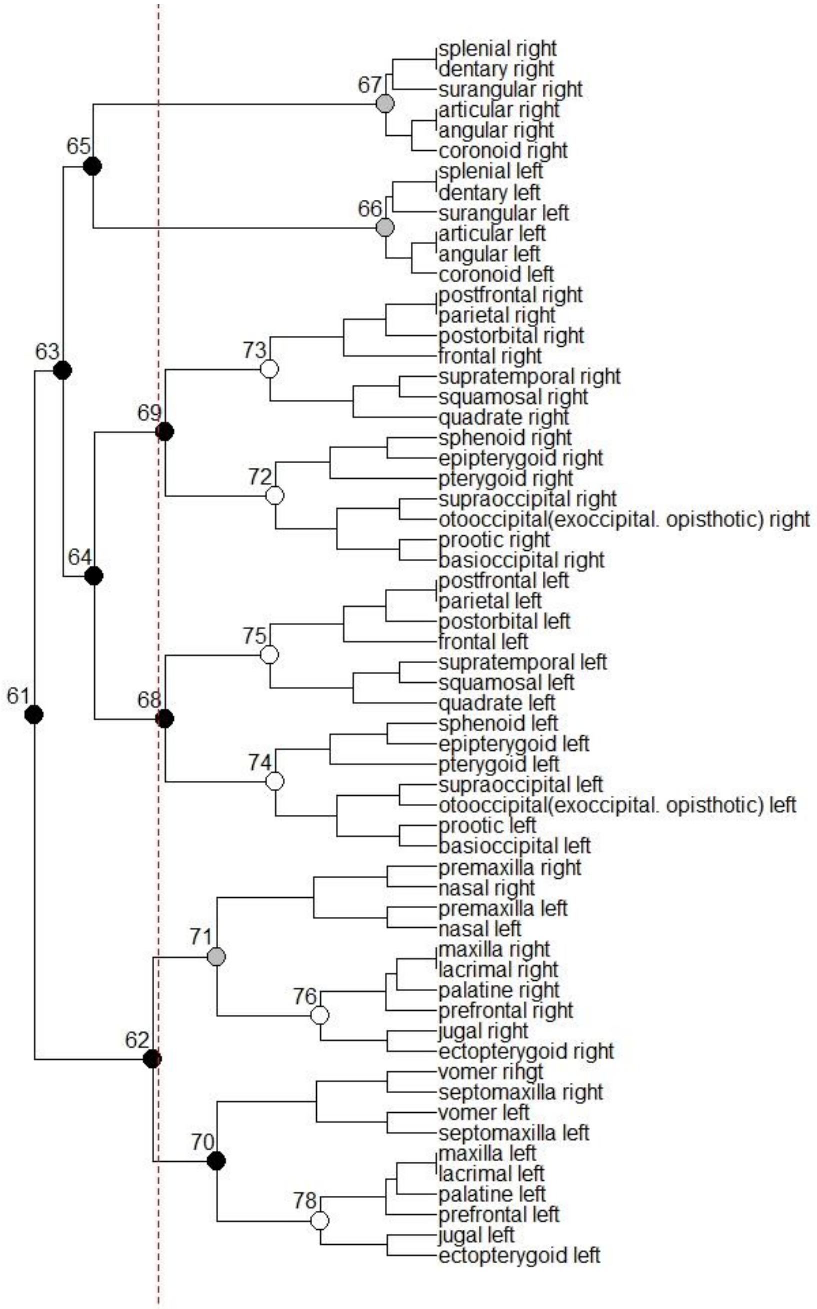

Supplementary Figure 11. The skull network dendrogram of *Cordylus cordylus*.

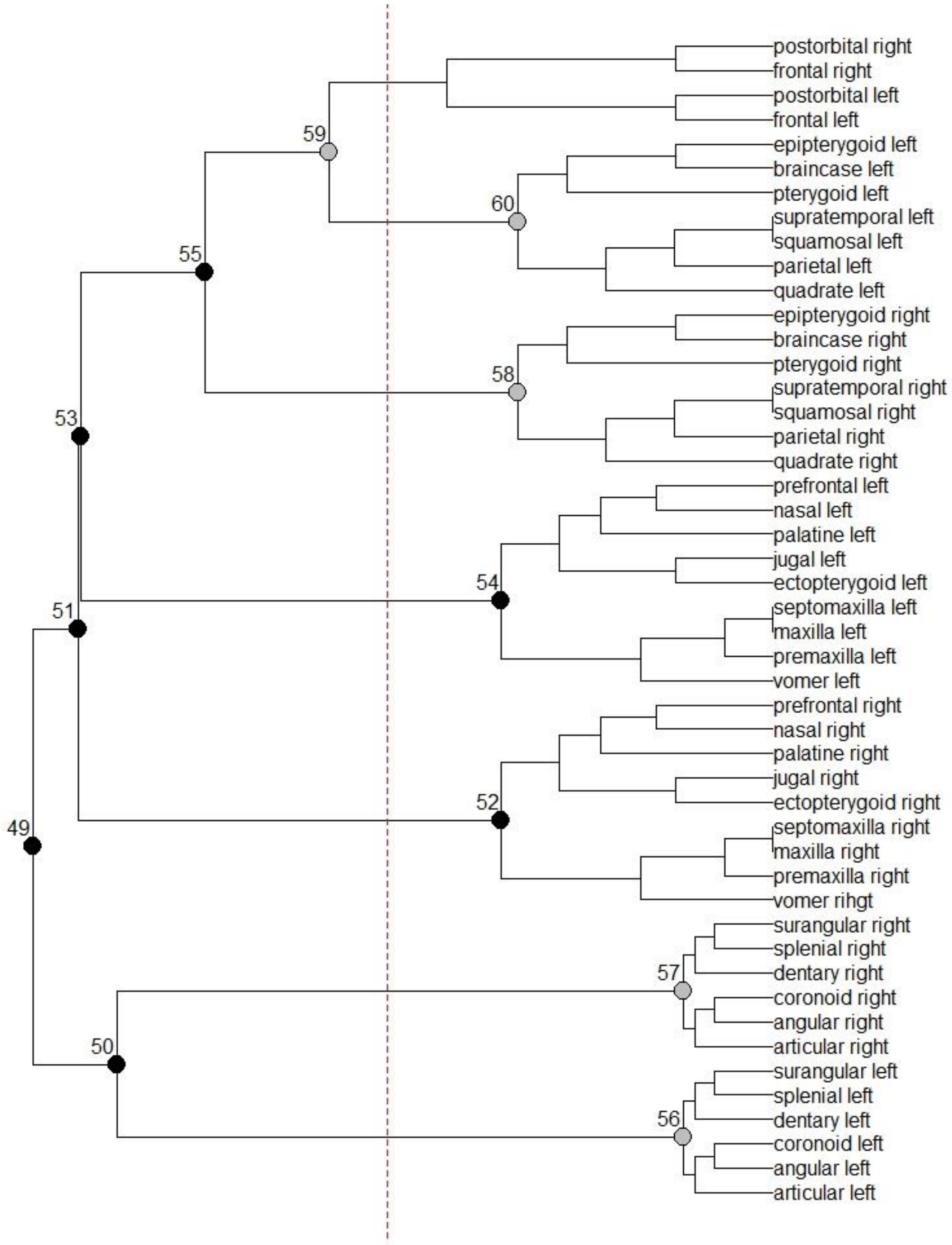

Supplementary Figure 12. The skull network dendrogram of *Crotaphytus collaris*.

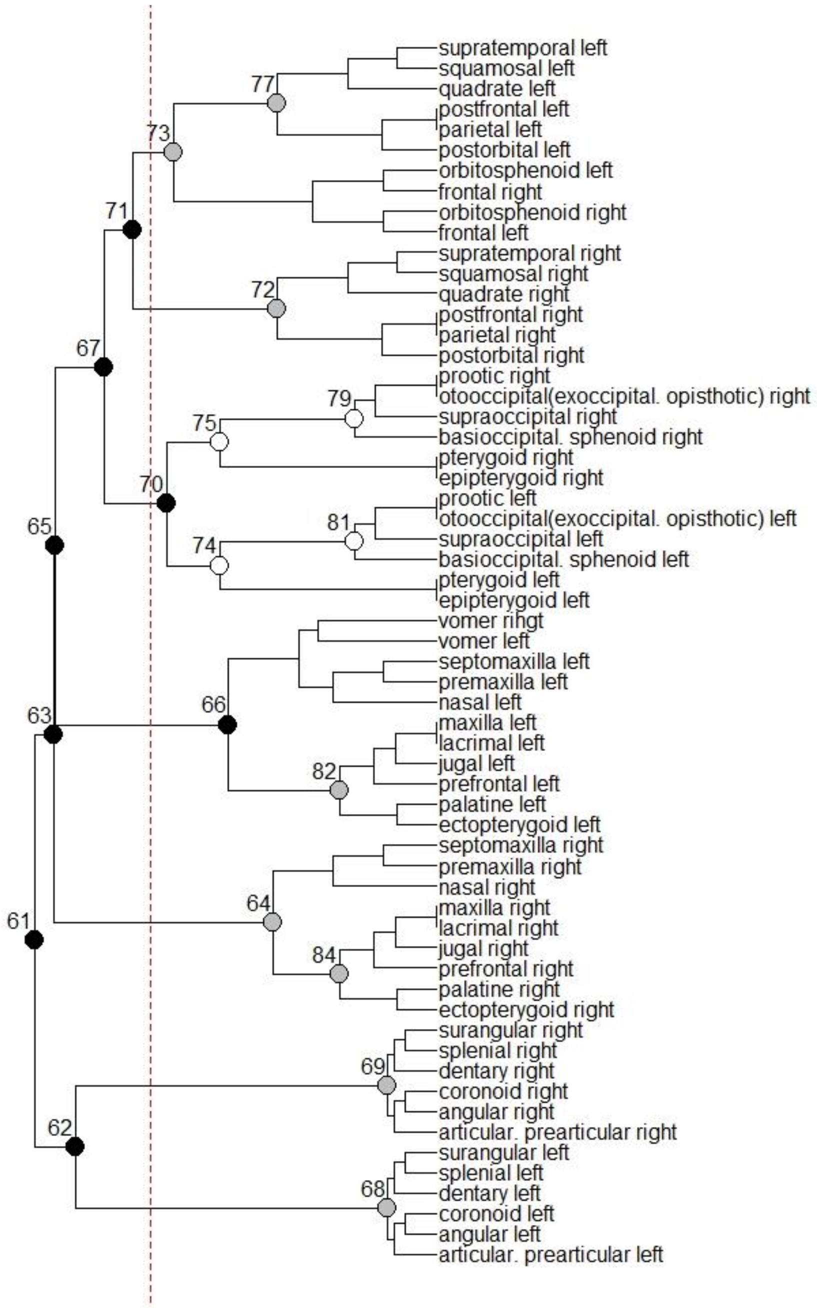

Supplementary Figure 13. The skull network dendrogram of *Cyclura carinata*.

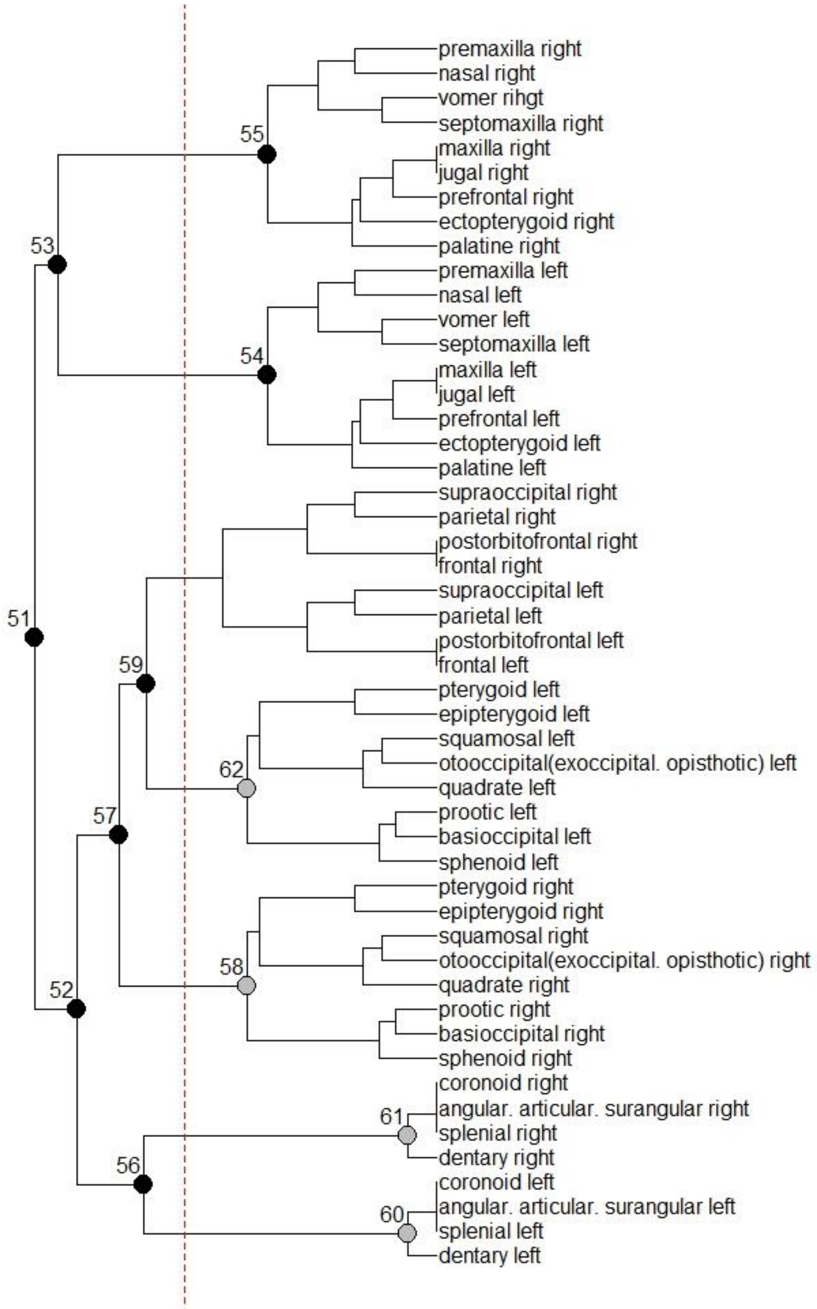

Supplementary Figure 14. The skull network dendrogram of *Cyrtodactylus pulchellus*.

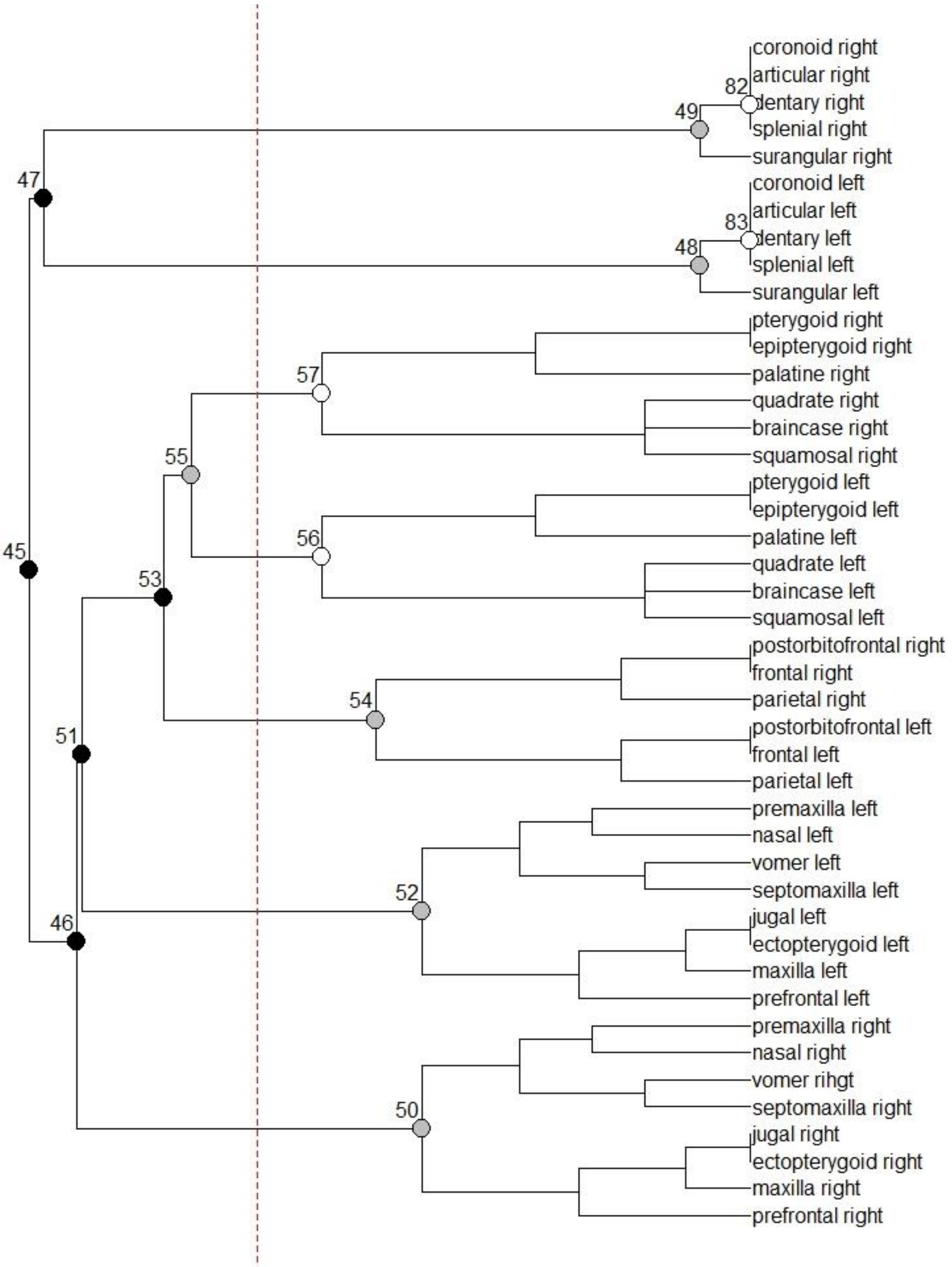

Supplementary Figure 15. The skull network dendrogram of *Delma borea*.

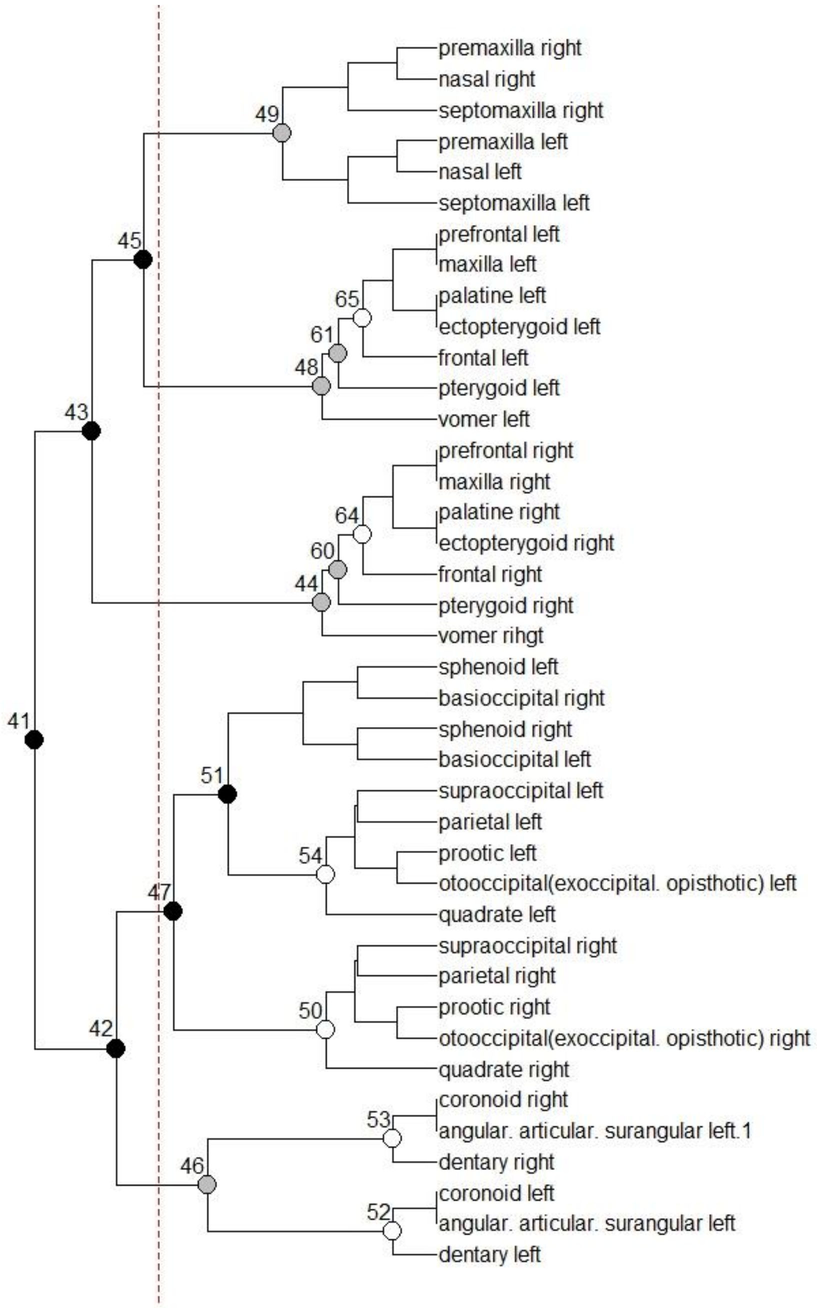

Supplementary Figure 16. The skull network dendrogram of *Dibamus novaeguineae*.

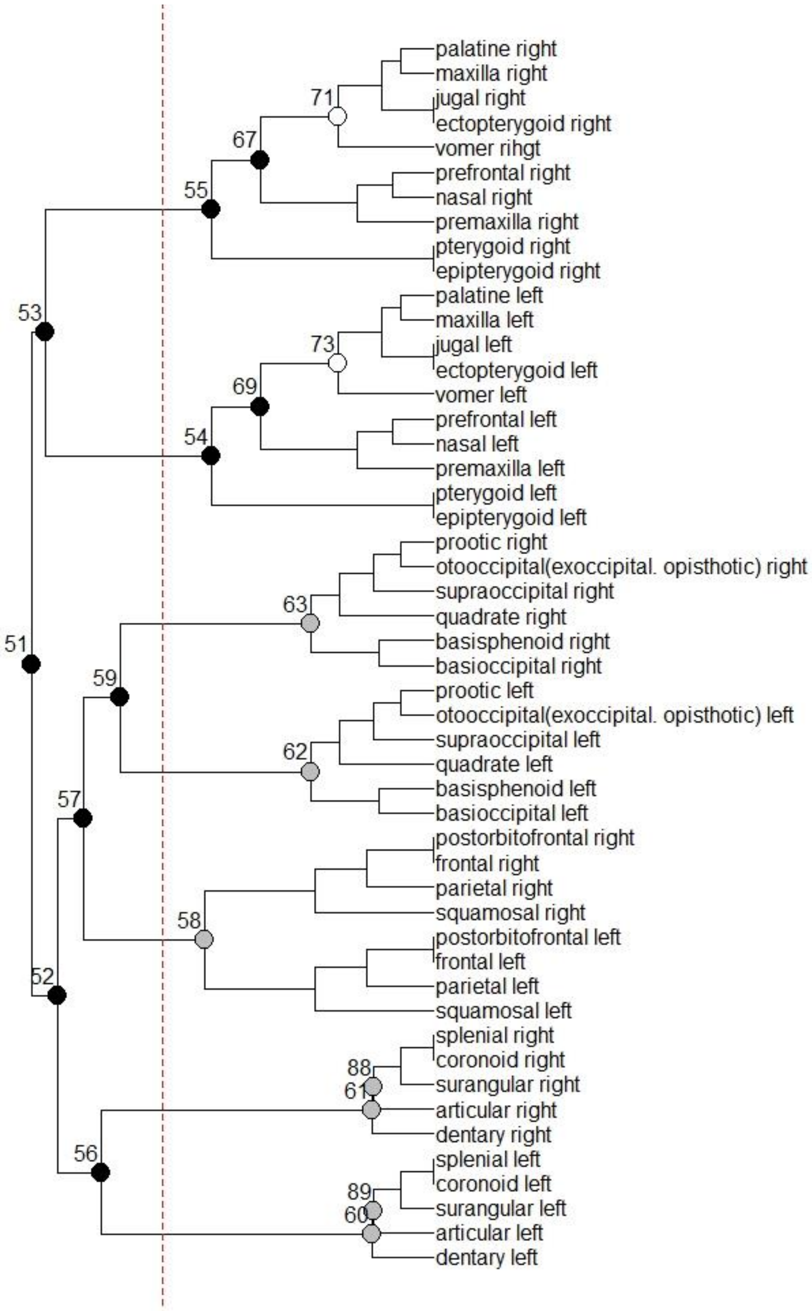

Supplementary Figure 17. The skull network dendrogram of *Diplodactylus pulcher*.

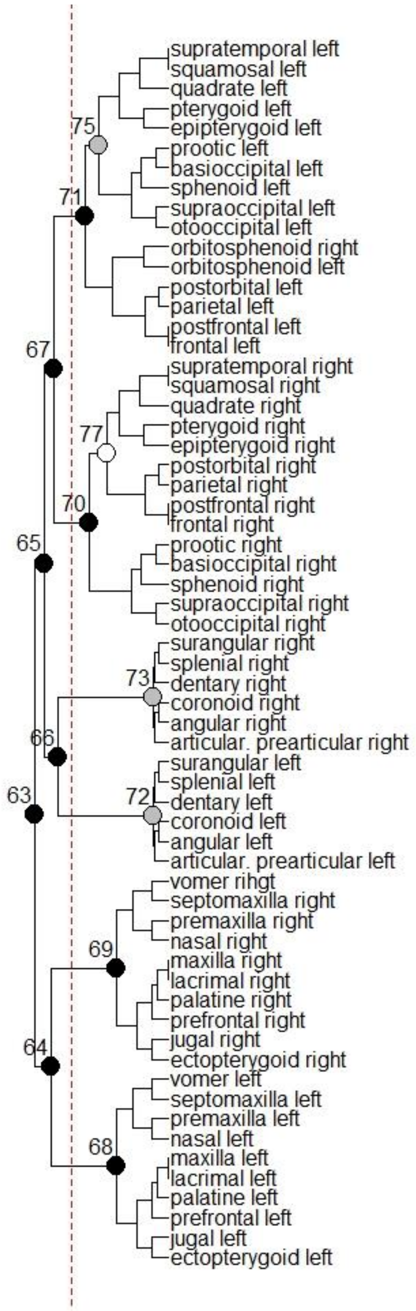

Supplementary Figure 18. The skull network dendrogram of *Dipsosaurus dorsalis*.

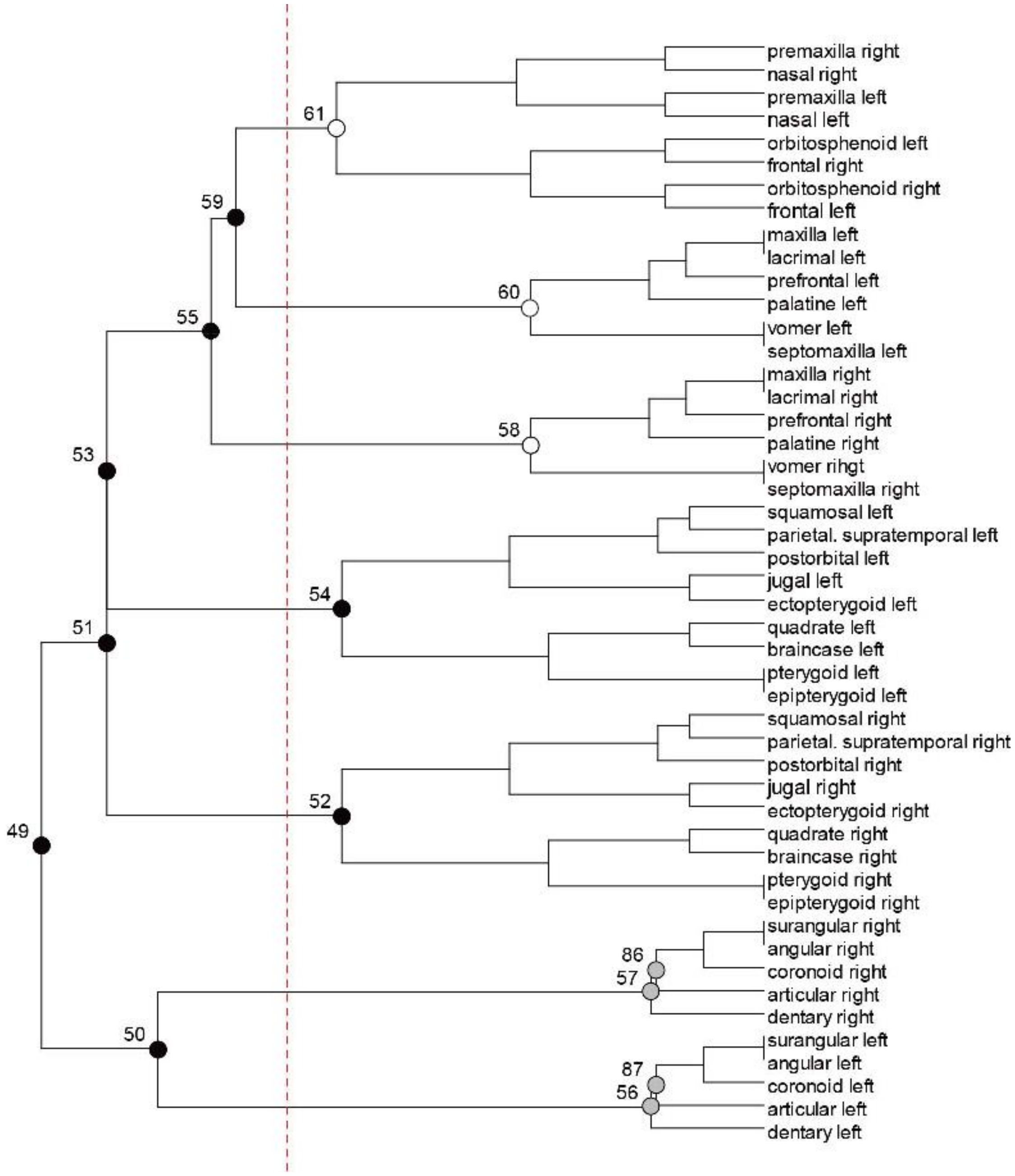

Supplementary Figure 19. The skull network dendrogram of *Draco volans*.

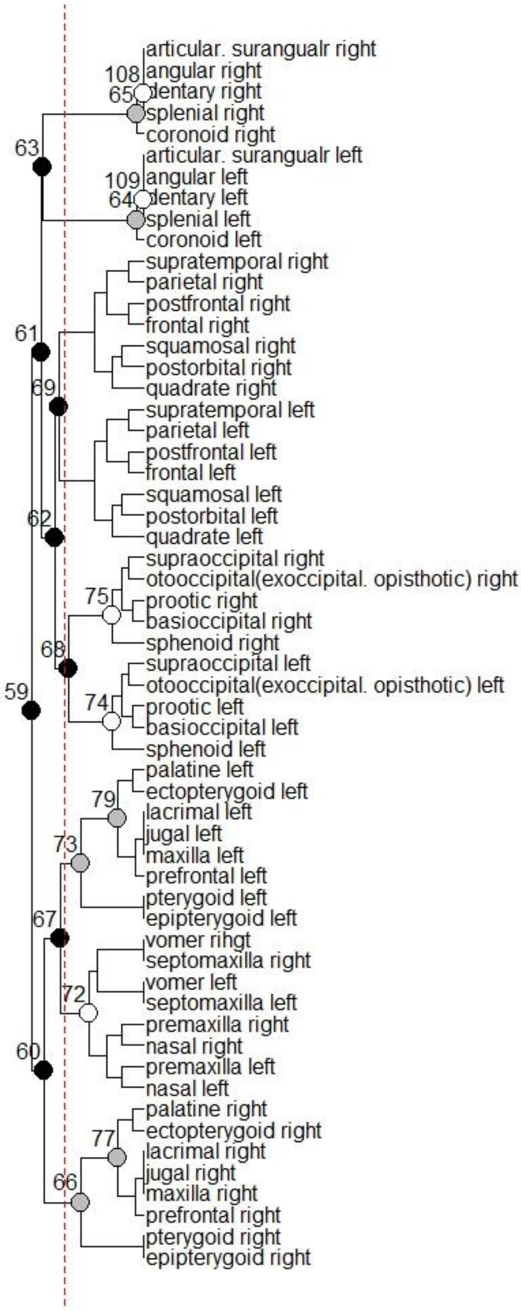

Supplementary Figure 20. The skull network dendrogram of *Elgaria panamintina*.

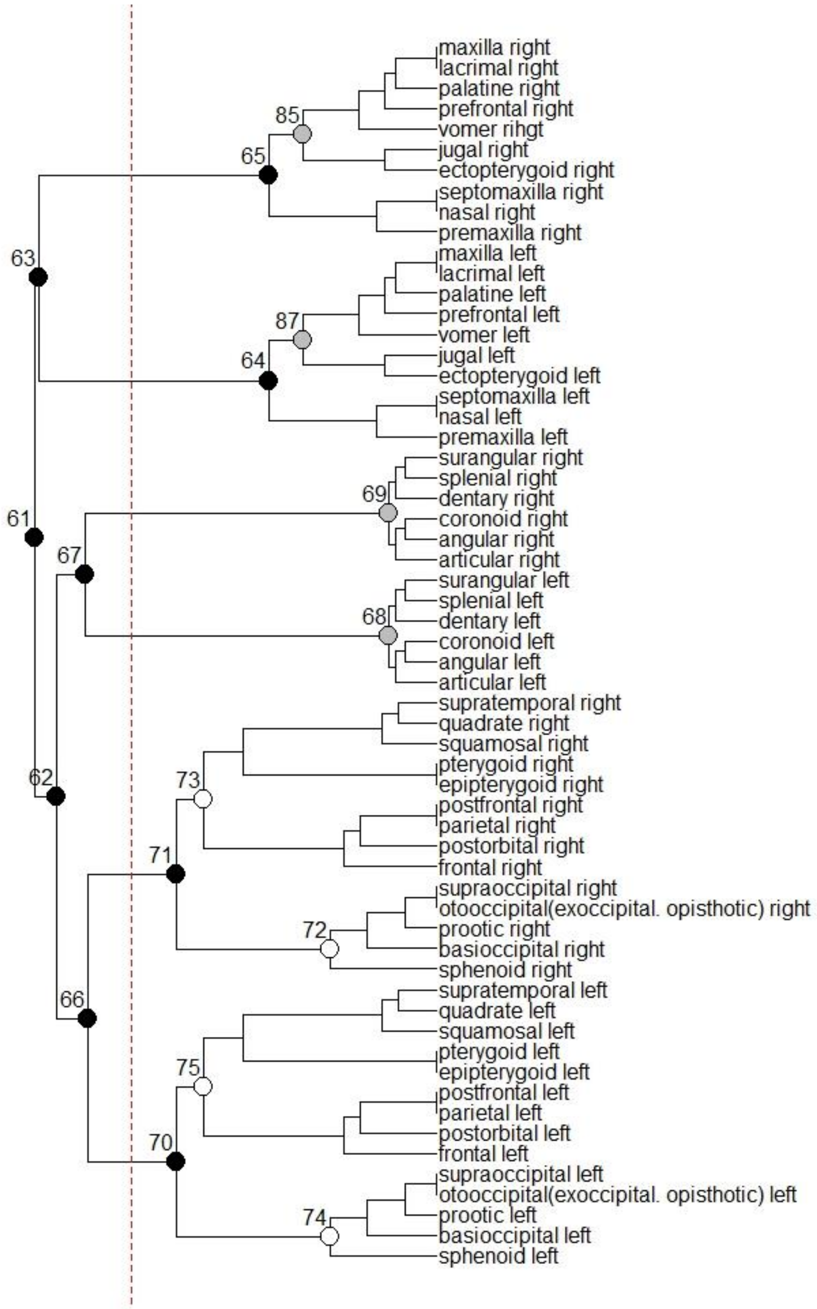

Supplementary Figure 21. The skull network dendrogram of *Enyalioides heterolepis*.

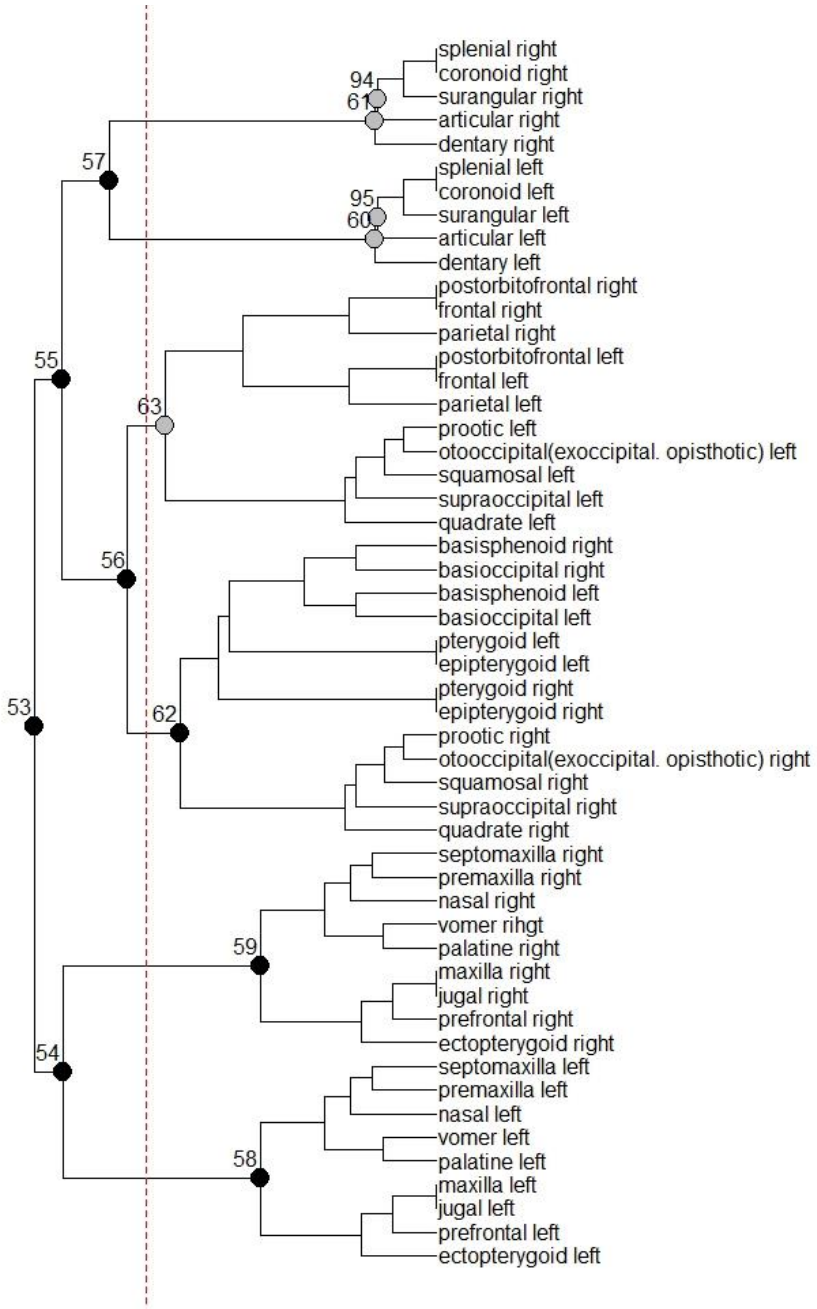

Supplementary Figure 22. The skull network dendrogram of *Gekko japonicus*.

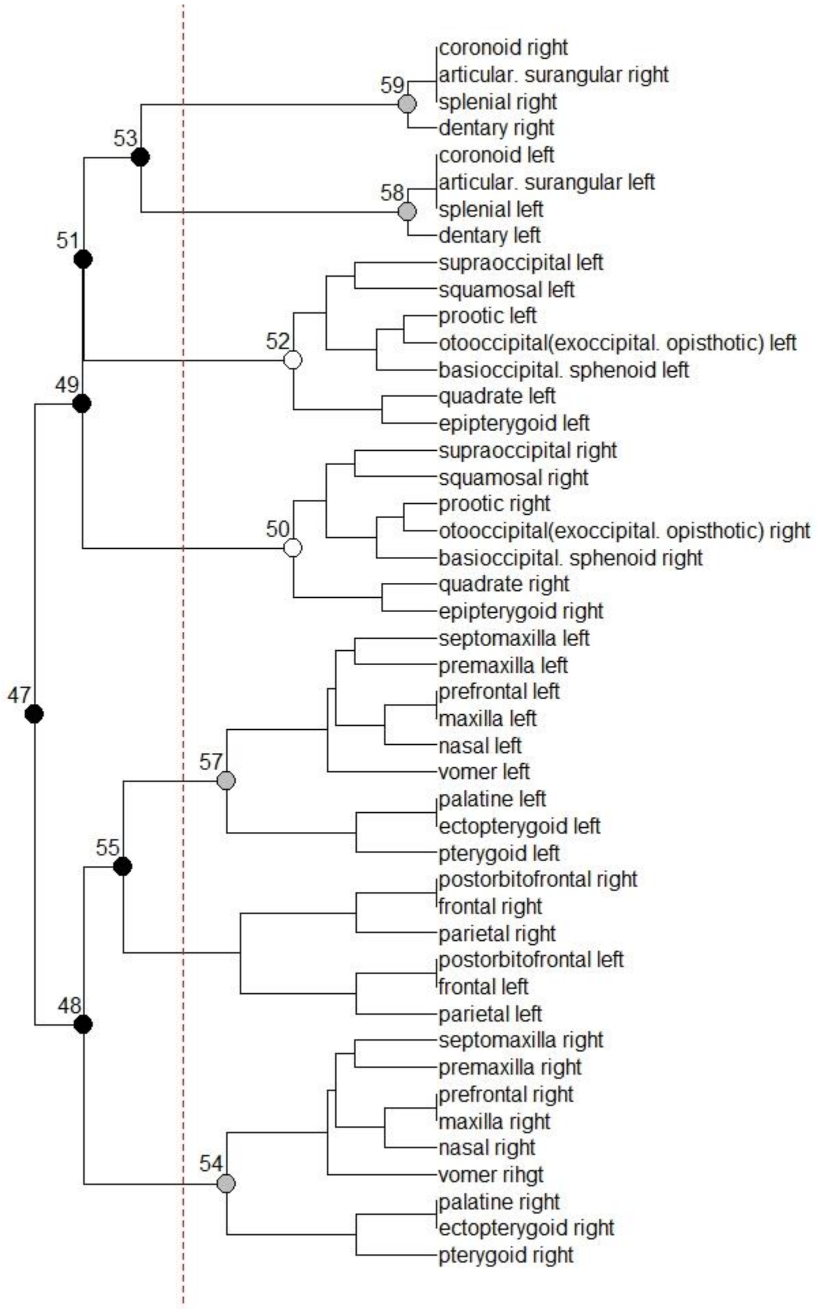

Supplementary Figure 23 The skull network dendrogram of *Gonatodes daudini*.

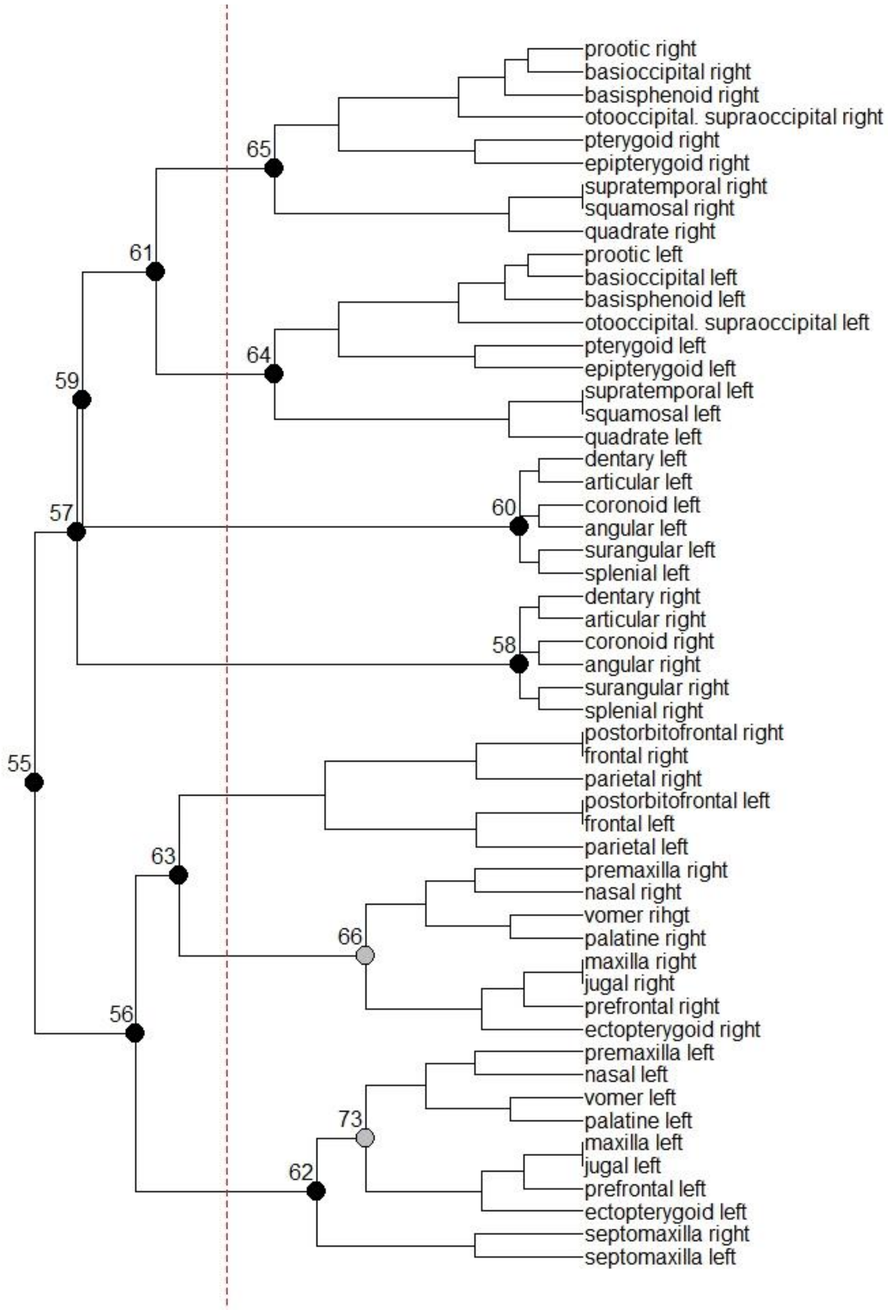

Supplementary Figure 24. The skull network dendrogram of *Goniurosaurus kuroiwae kuroiwae*.

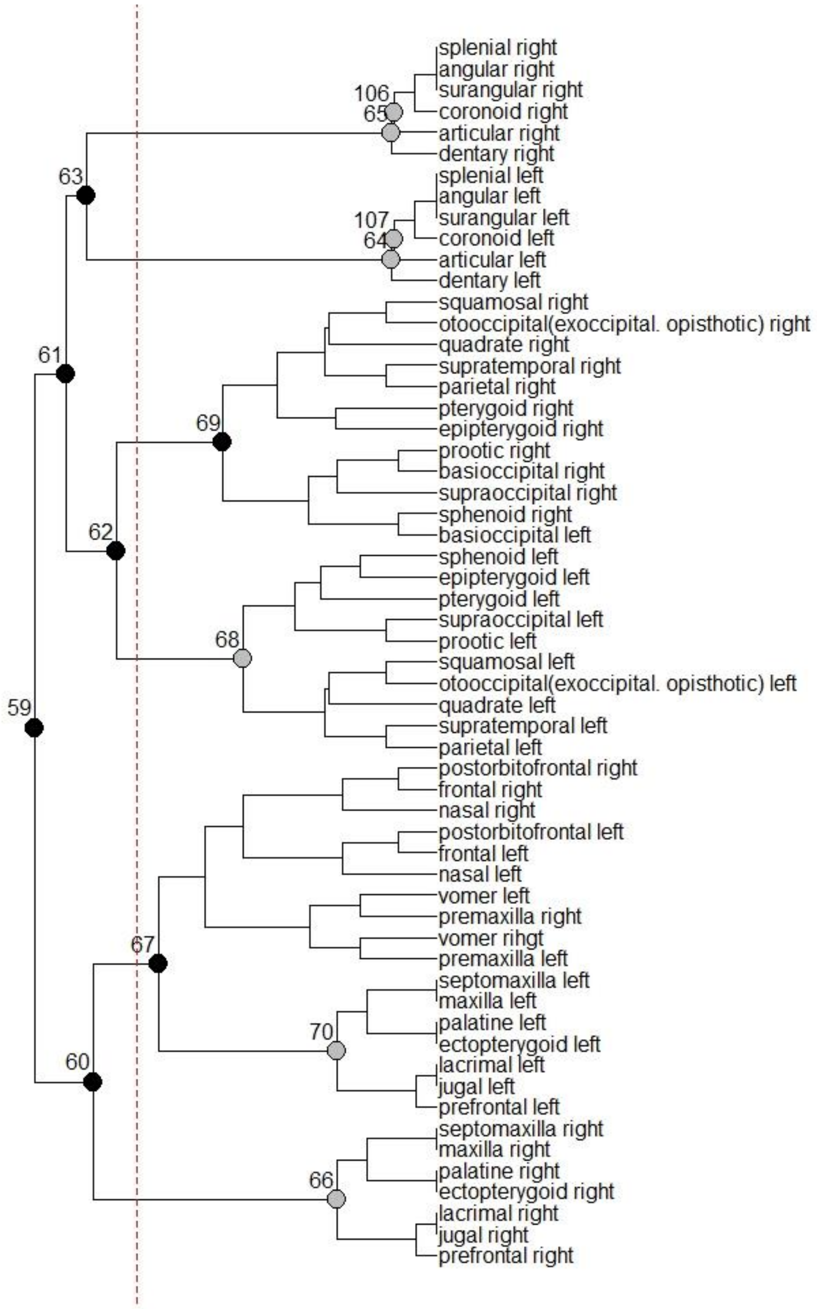

Supplementary Figure 25. The skull network dendrogram of *Heloderma horridum*.

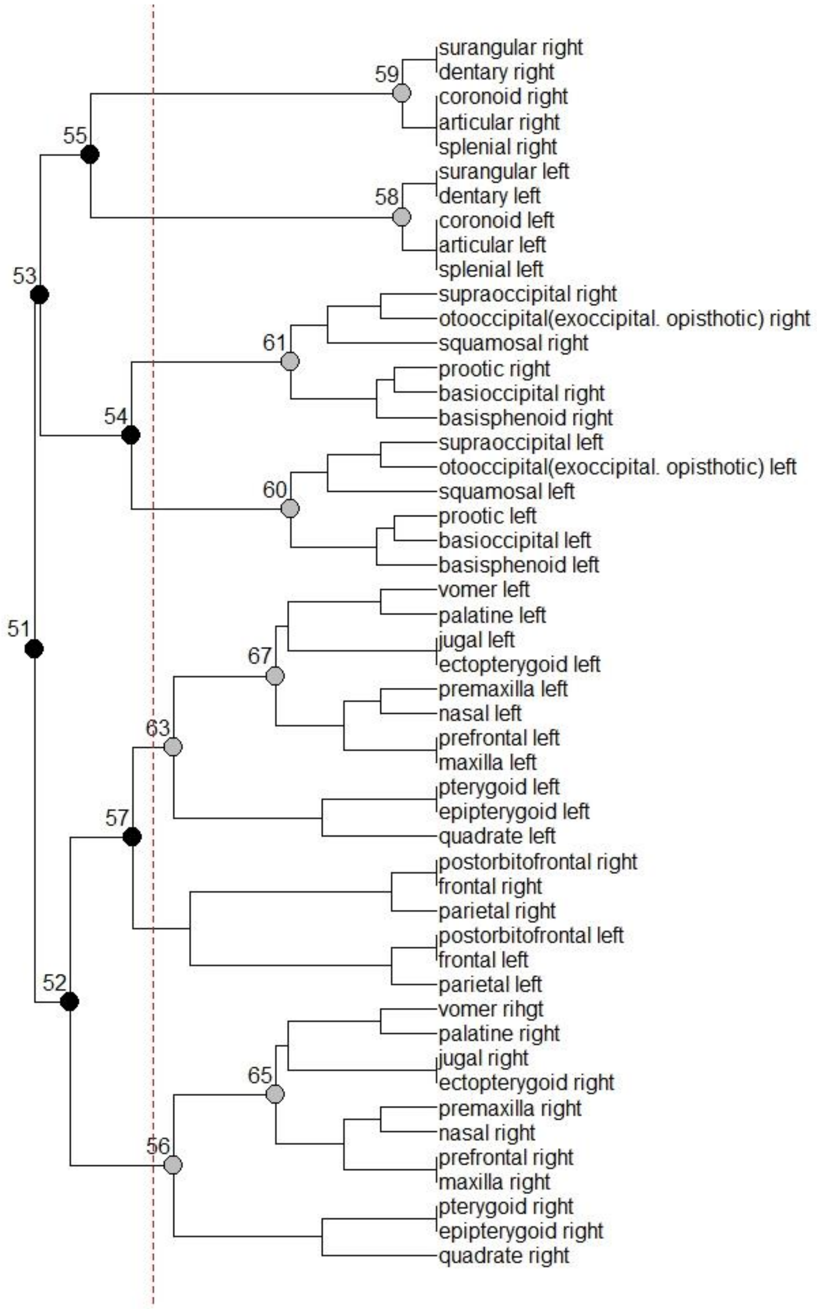

Supplementary Figure 26. The skull network dendrogram of *Homonota horrida*.

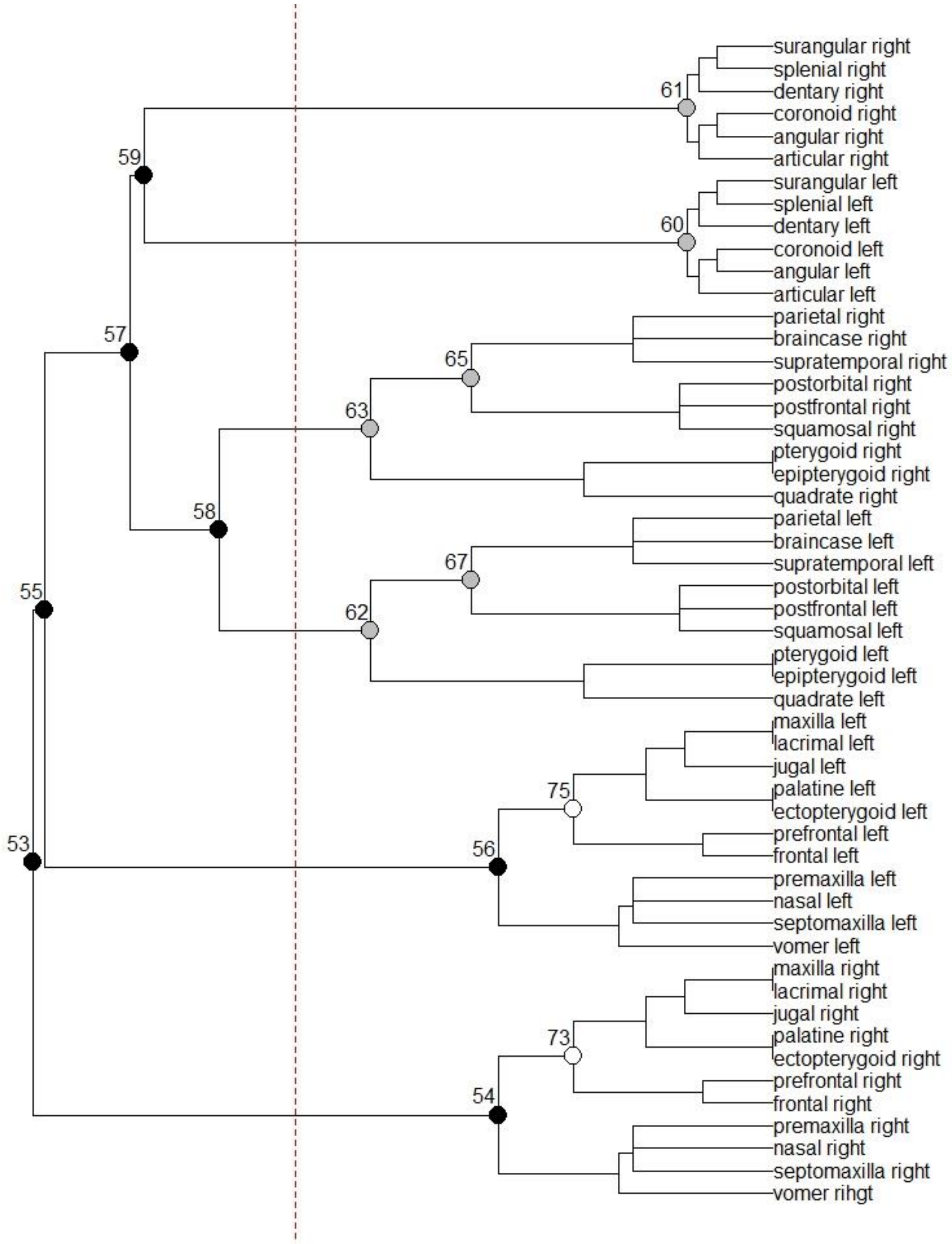

Supplementary Figure 27. The skull network dendrogram of *Lacerta viridis*.

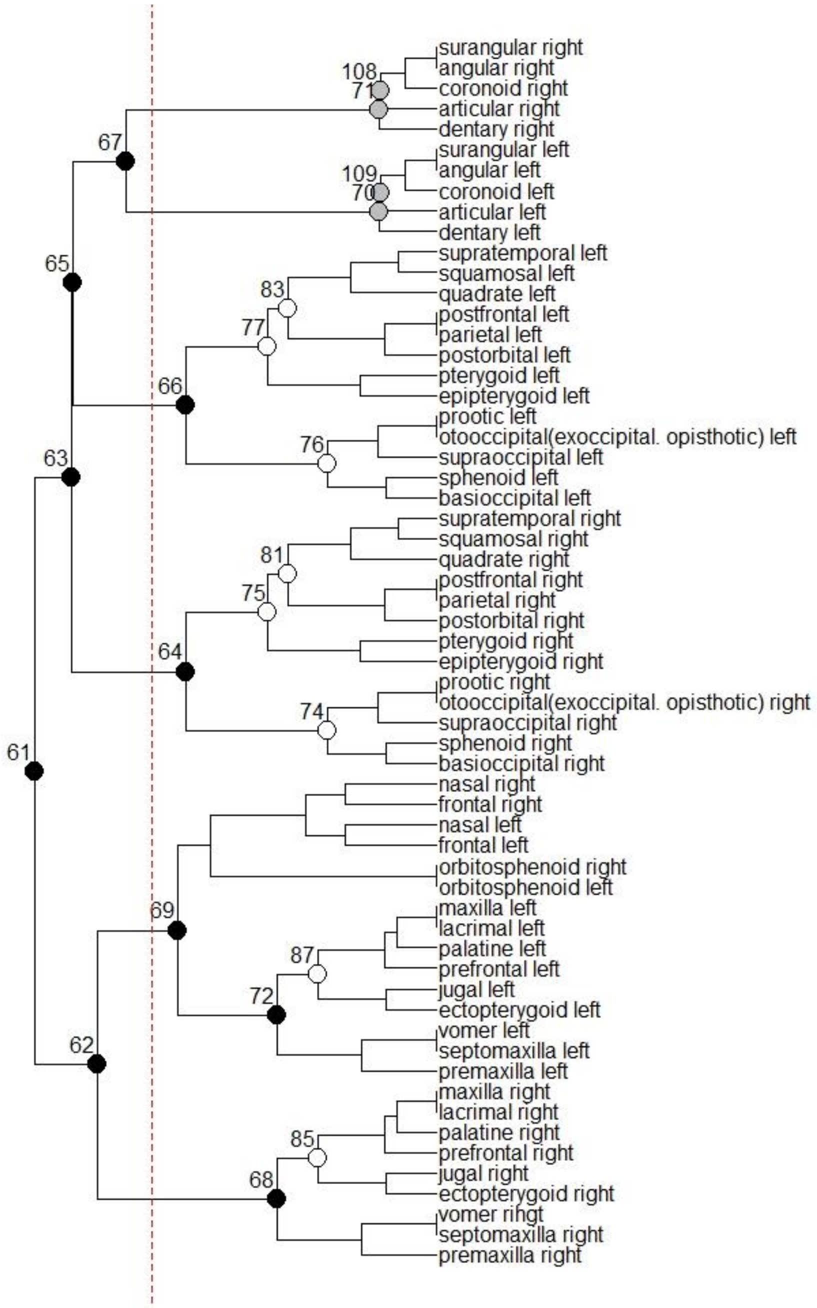

Supplementary Figure 28. The skull network dendrogram of *Leiocephalus carinatus*.

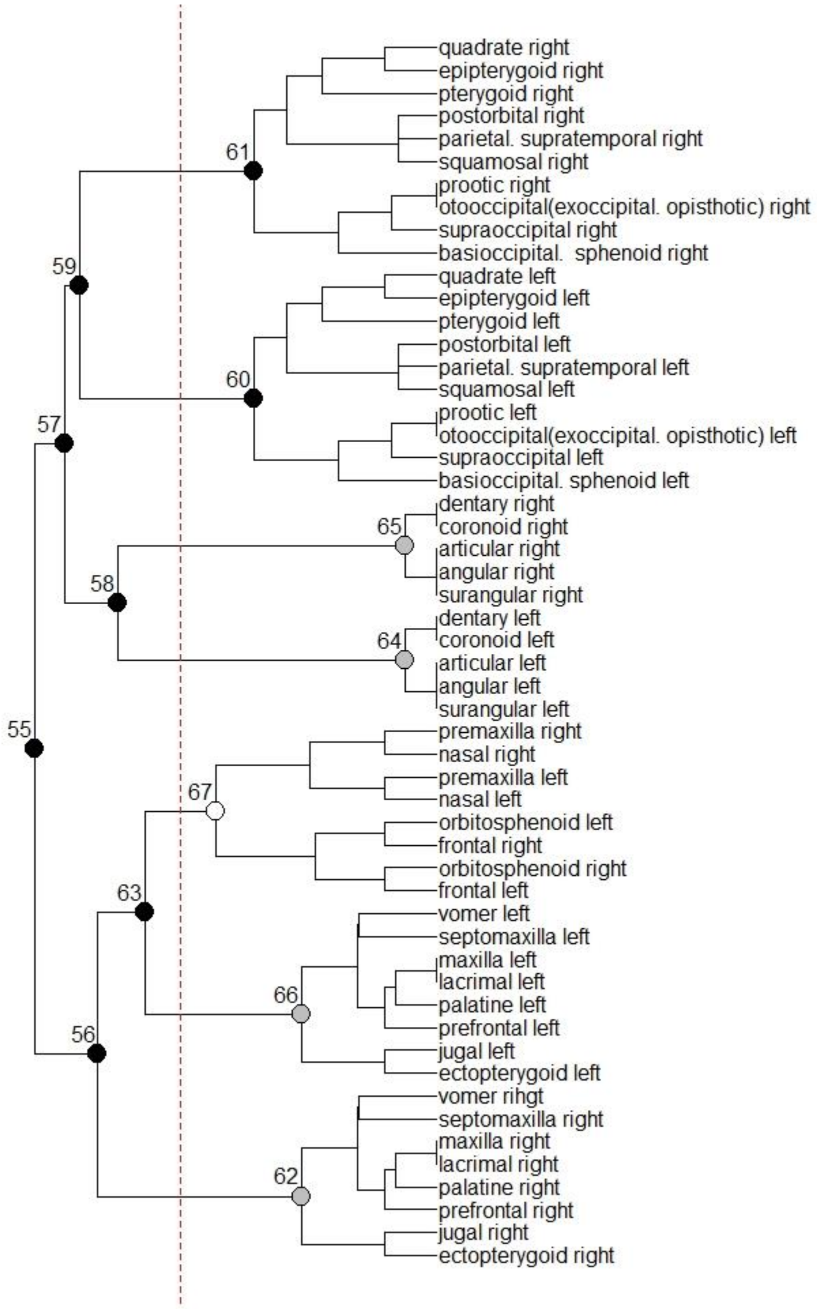

Supplementary Figure 29. The skull network dendrogram of *Leiolepis belliana*.

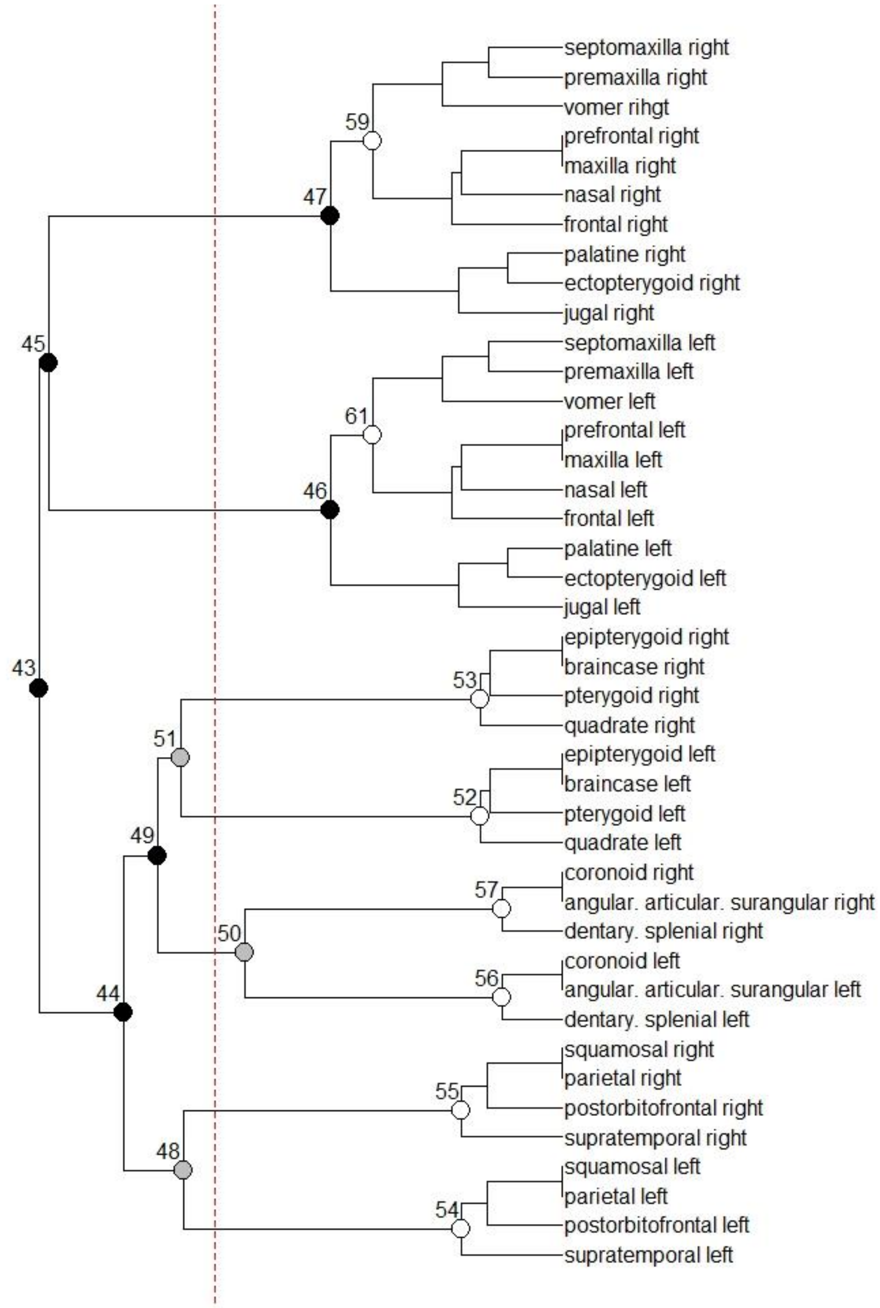

Supplementary Figure 30. The skull network dendrogram of *Lepidophyma flavimaculatum*.

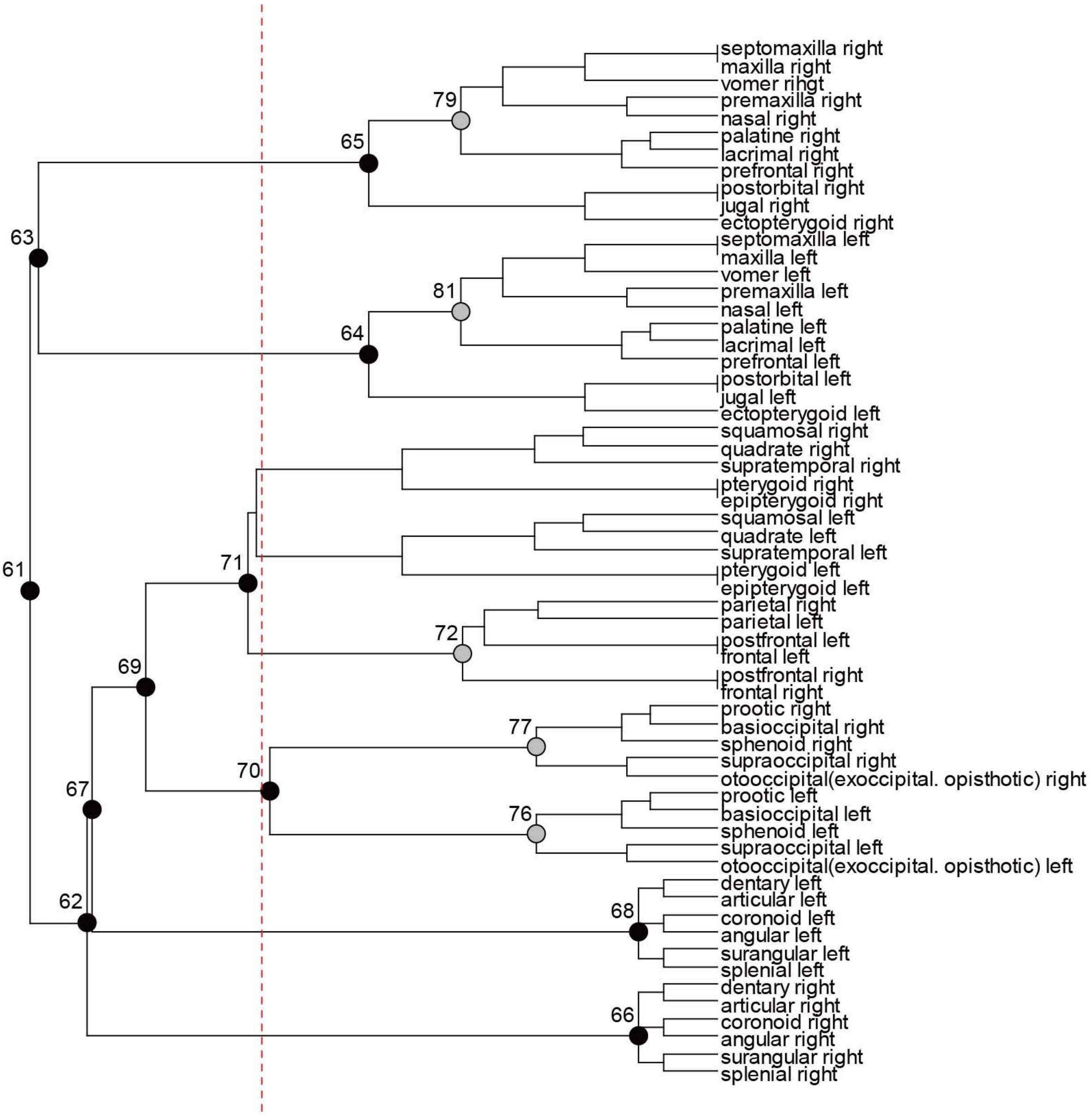

Supplementary Figure 31. The skull network dendrogram of *Liolaemus bellii*.

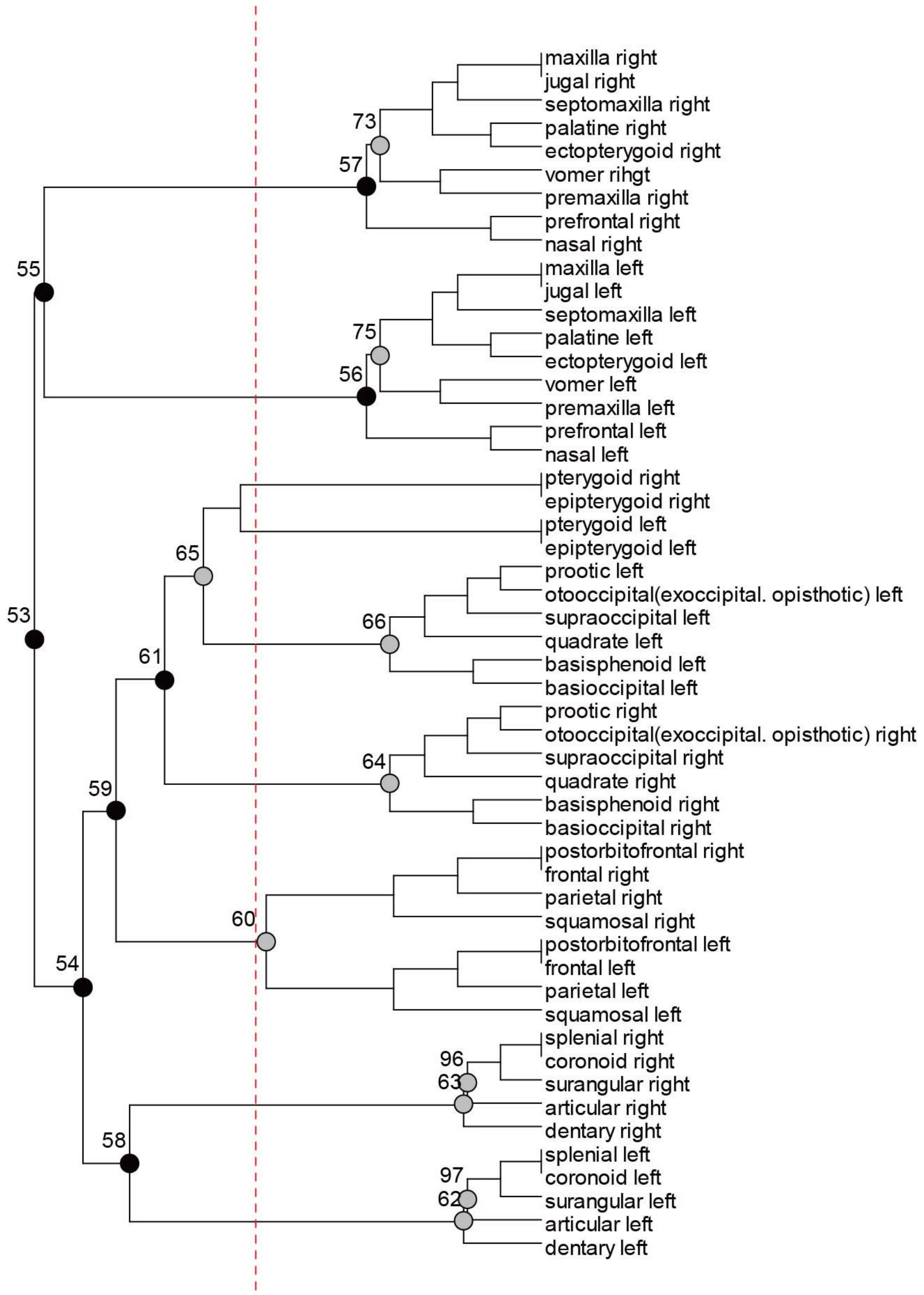

Supplementary Figure 32. The skull network dendrogram of *Oedura tryoni*.

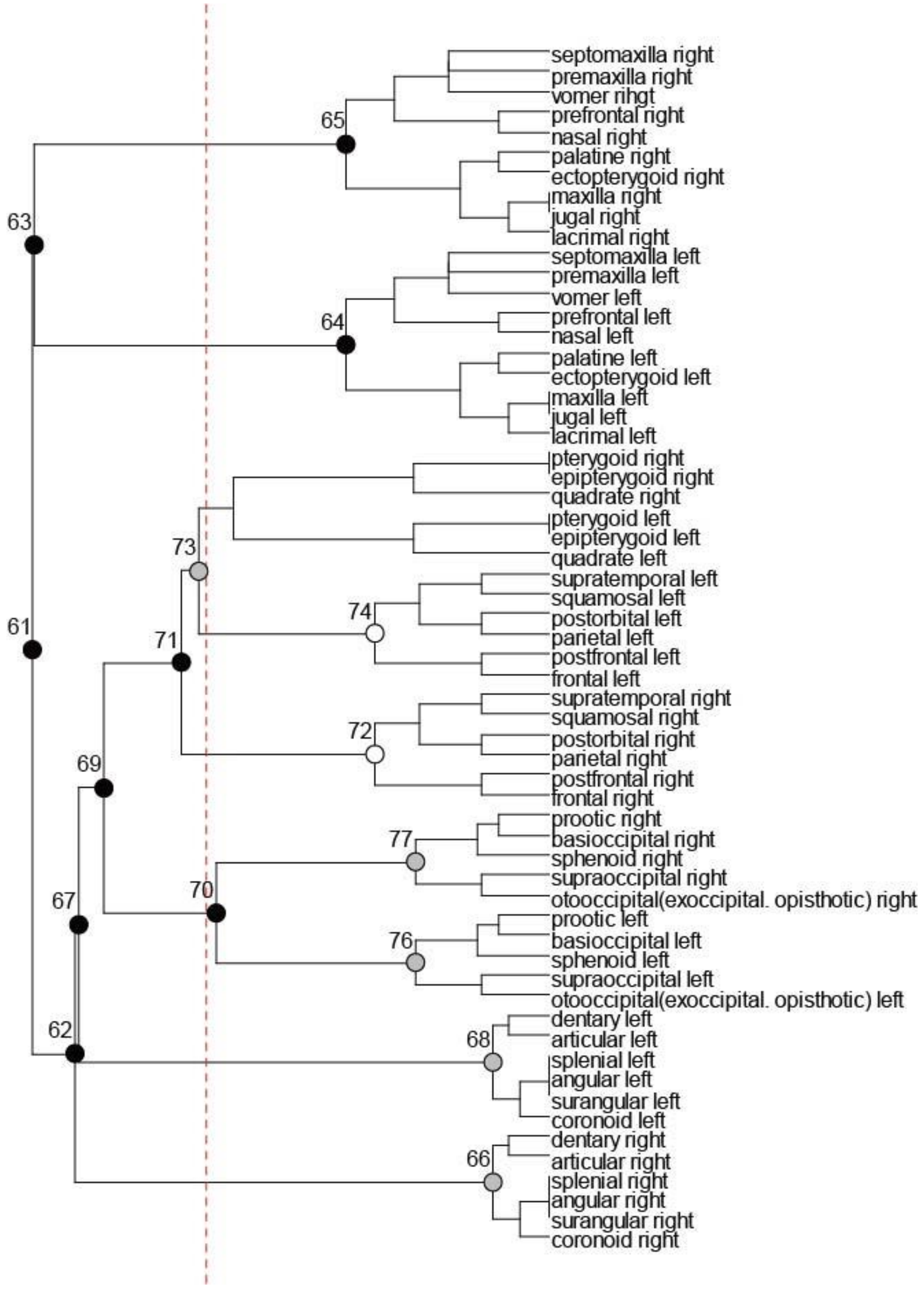

Supplementary Figure 33. The skull network dendrogram of *Ophisaurus mimicus*.

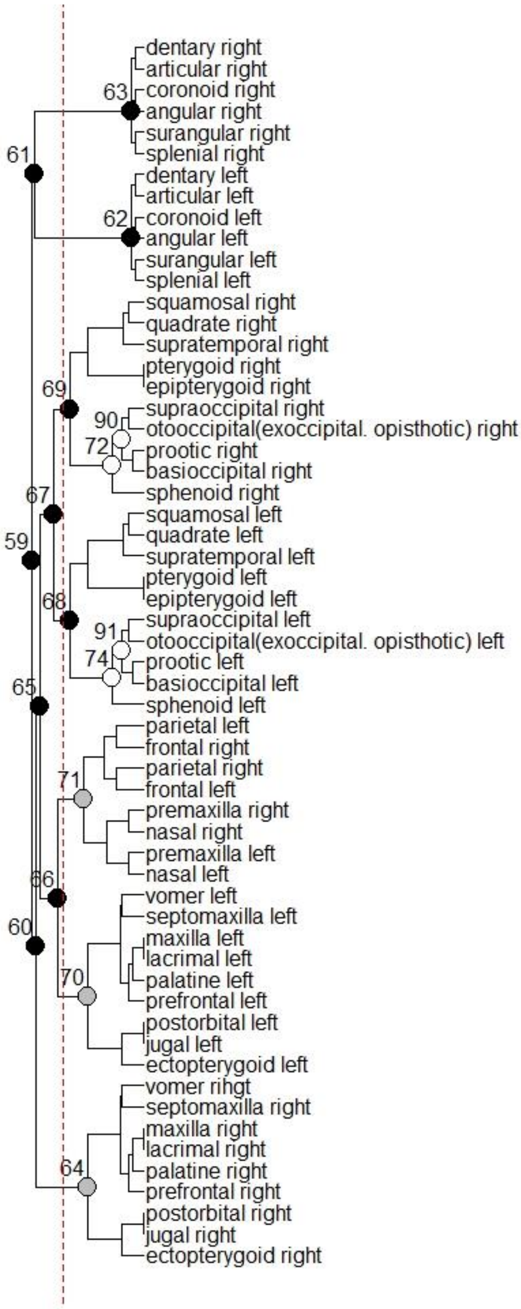

Supplementary Figure 34. The skull network dendrogram of *Oplurus cyclurus*.

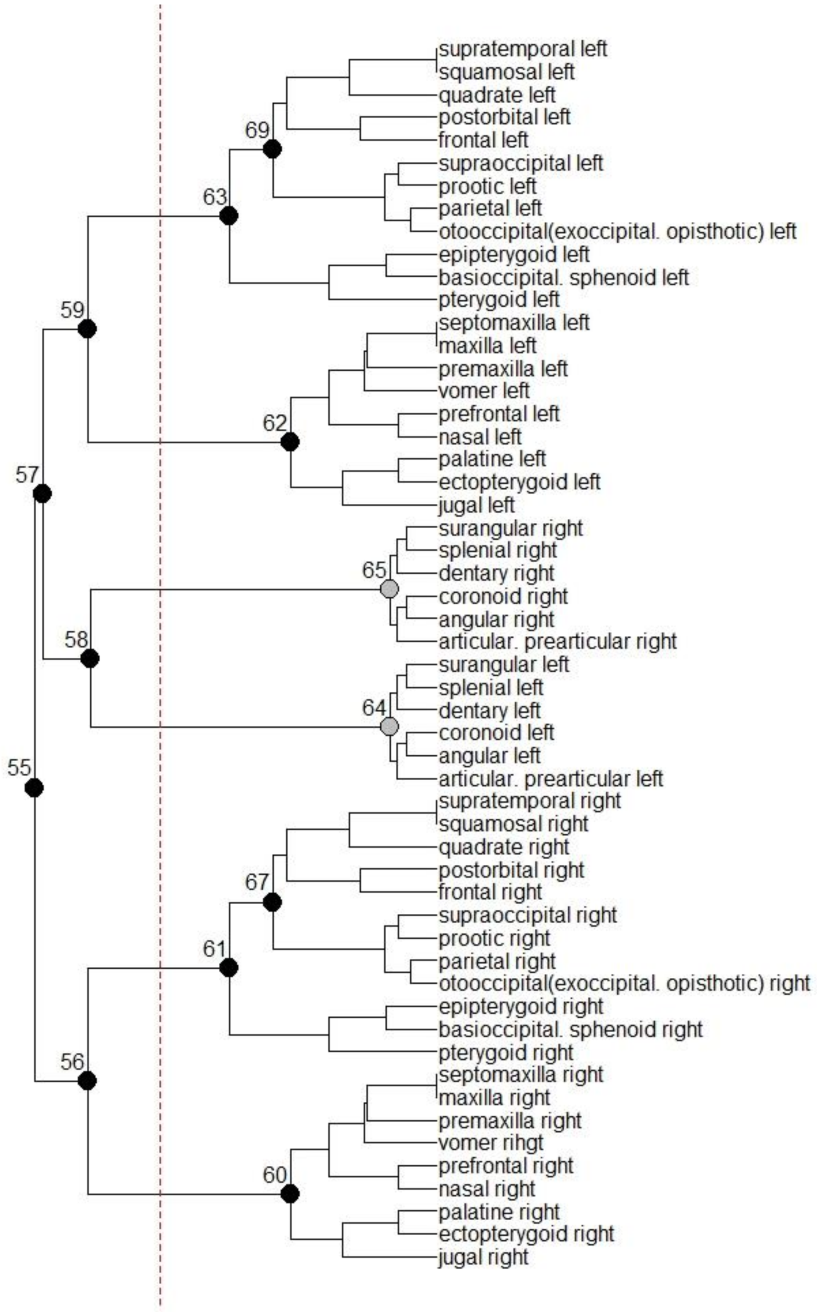

Supplementary Figure 35. The skull network dendrogram of *Phrynosoma asio*.

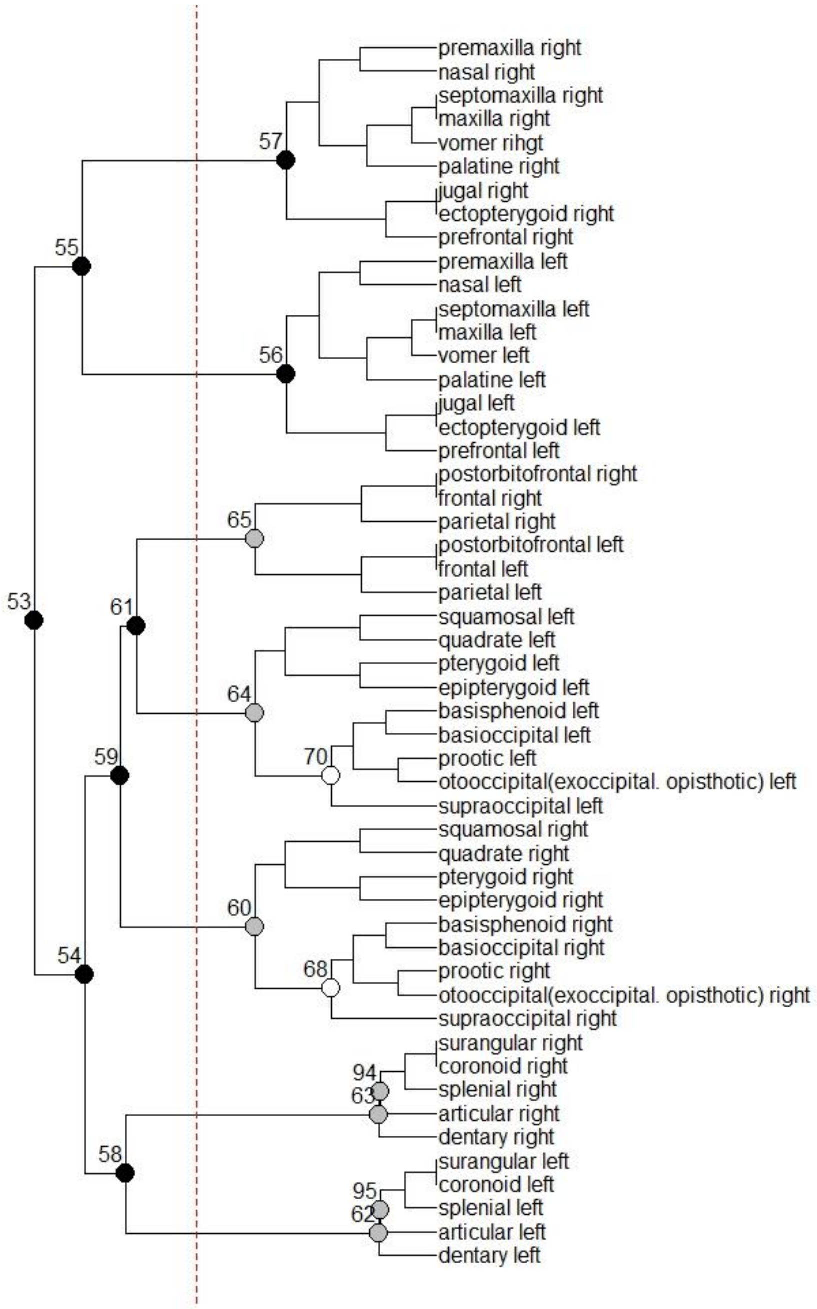

Supplementary Figure 36. The skull network dendrogram of *Phyllurus platurus*.

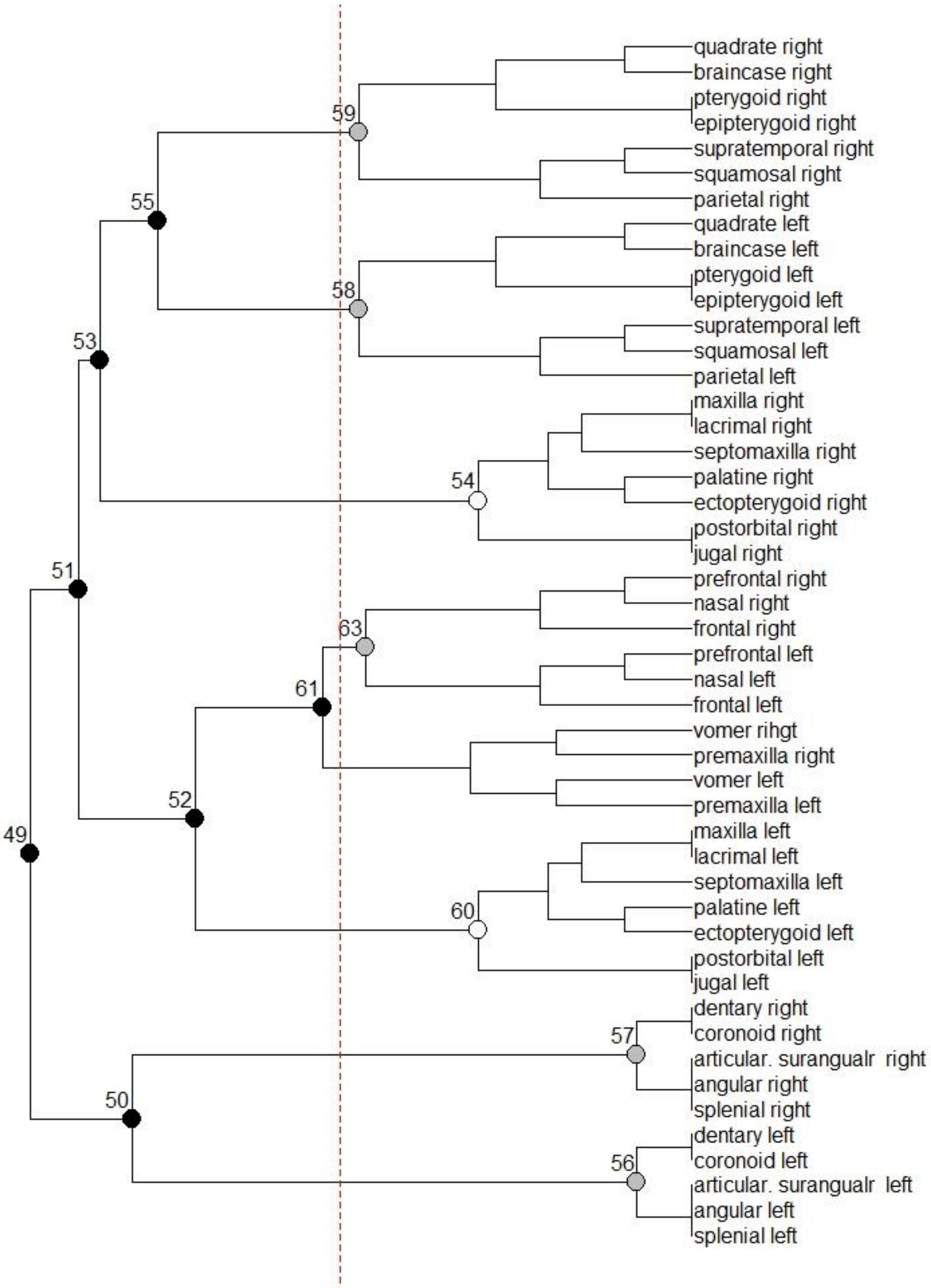

Supplementary Figure 37. The skull network dendrogram of *Phymaturus palluma*.

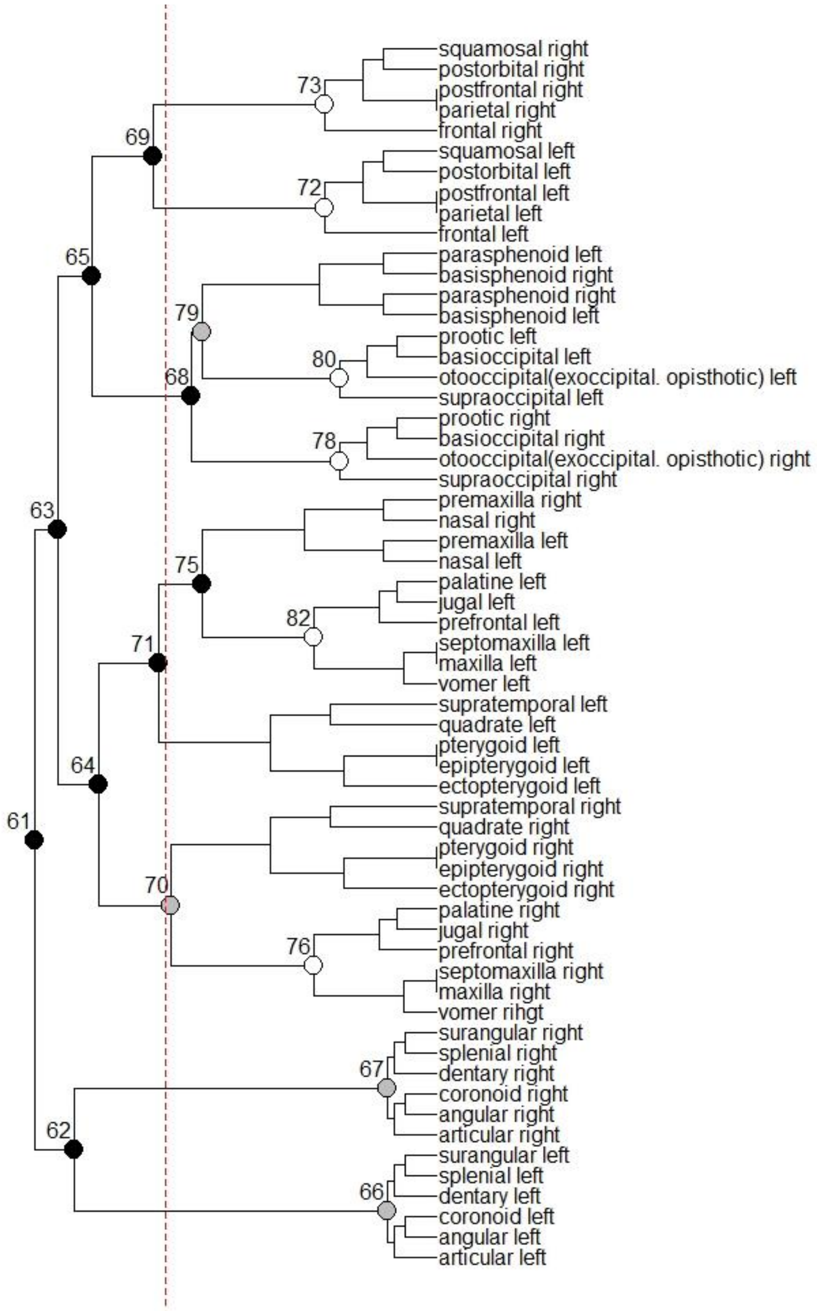

Supplementary Figure 38. The skull network dendrogram of *Platysaurus intermedius wilhelmi*.

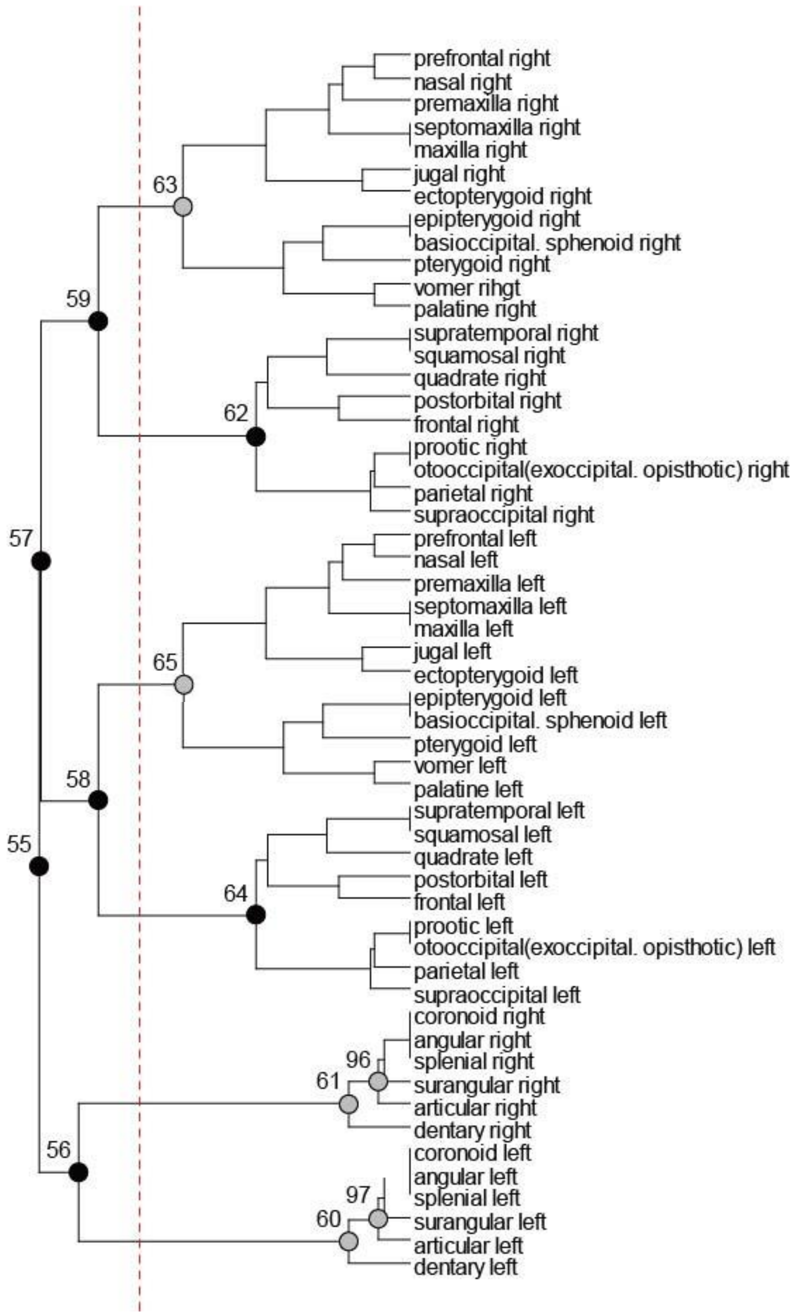

Supplementary Figure 39. The skull network dendrogram of *Pogona vitticeps*.

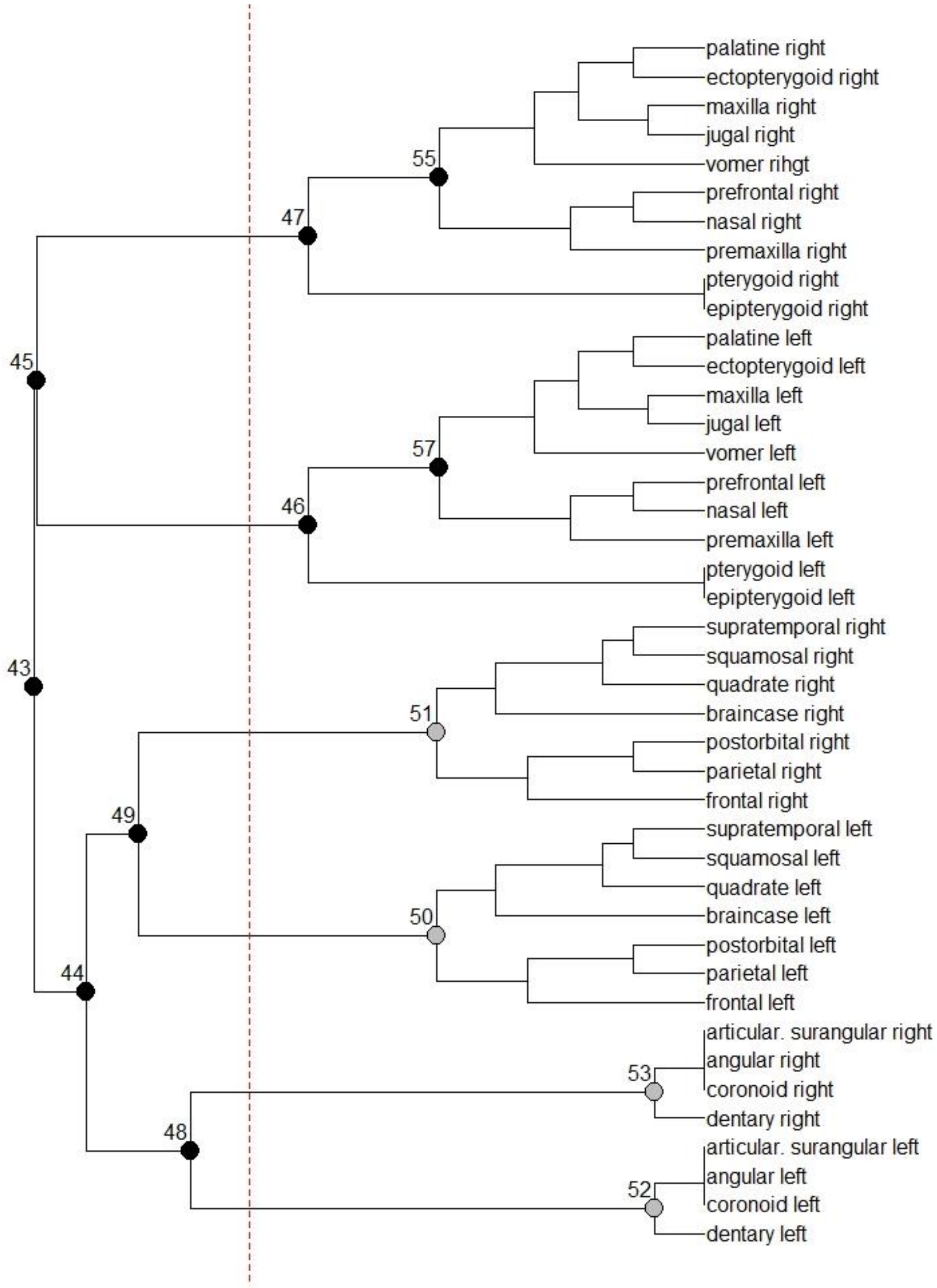

Supplementary Figure 40. The skull network dendrogram of *Polychrus marmoratus*.

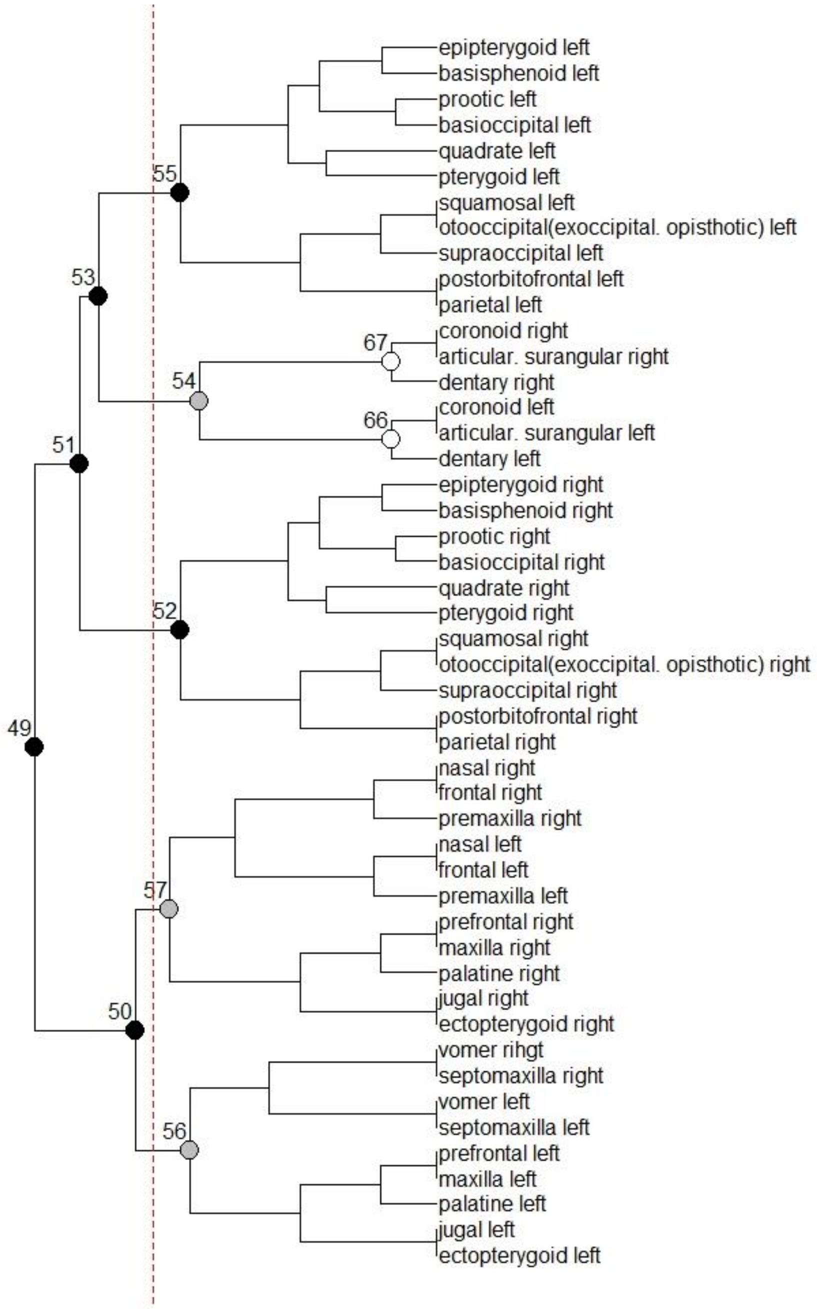

Supplementary Figure 41. The skull network dendrogram of *Pristurus carteri*.

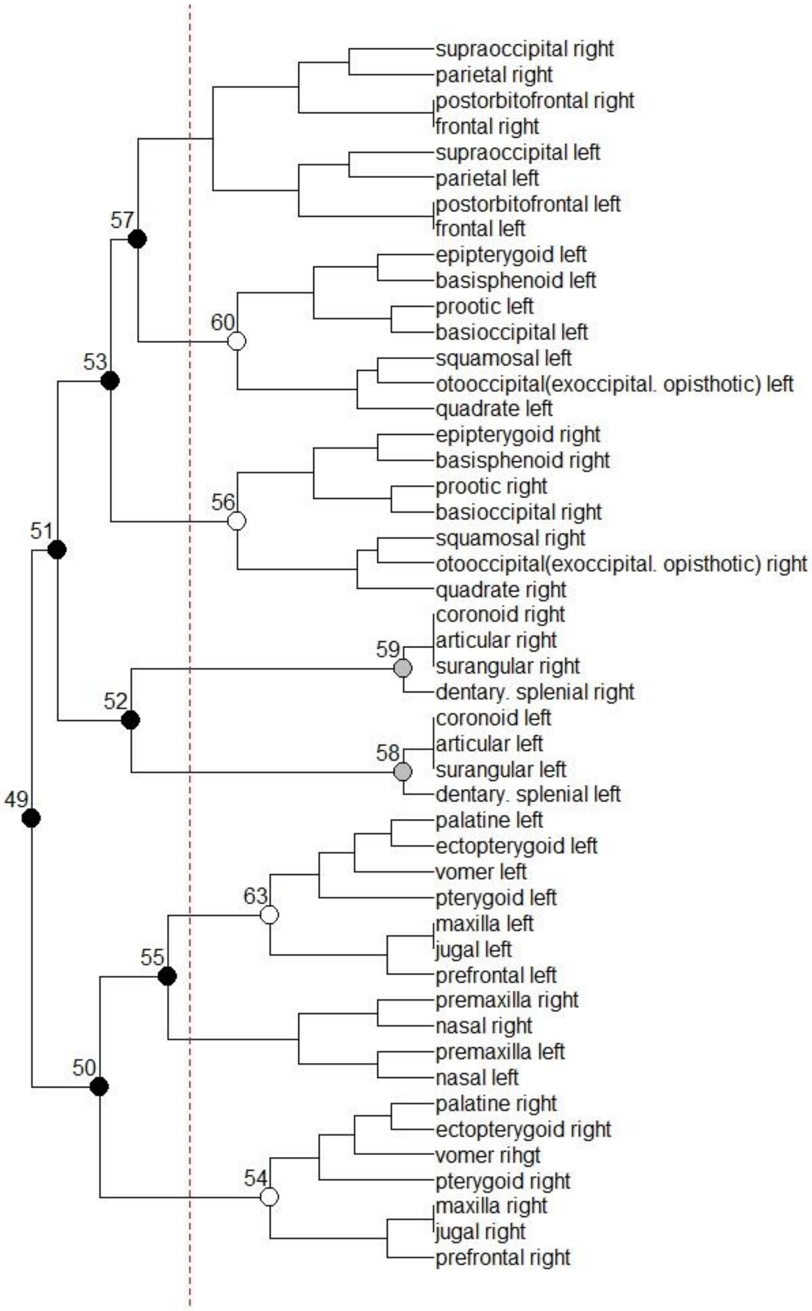

Supplementary Figure 42. The skull network dendrogram of *Ptyodactylus hasselquistii*.

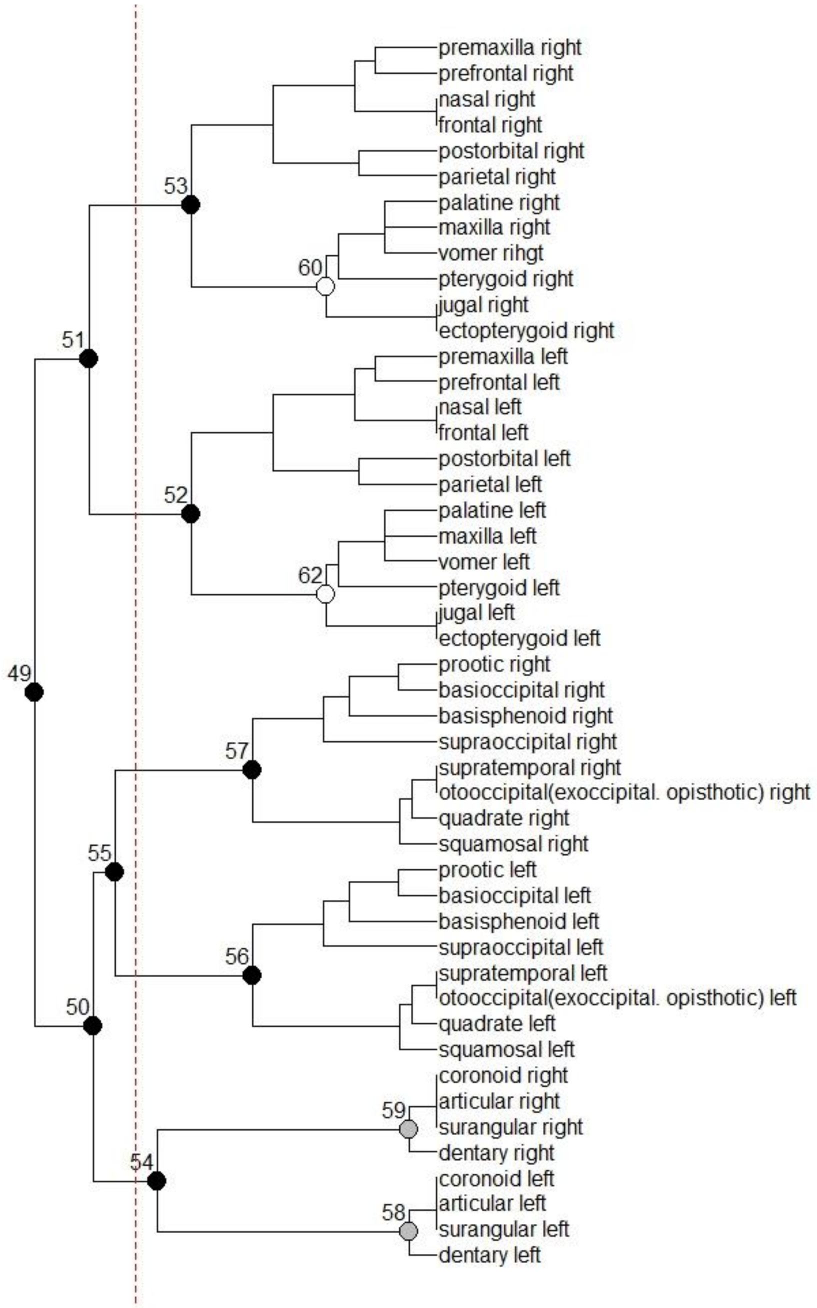

Supplementary Figure 43. The skull network dendrogram of *Rhampholeon brevicaudatus*.

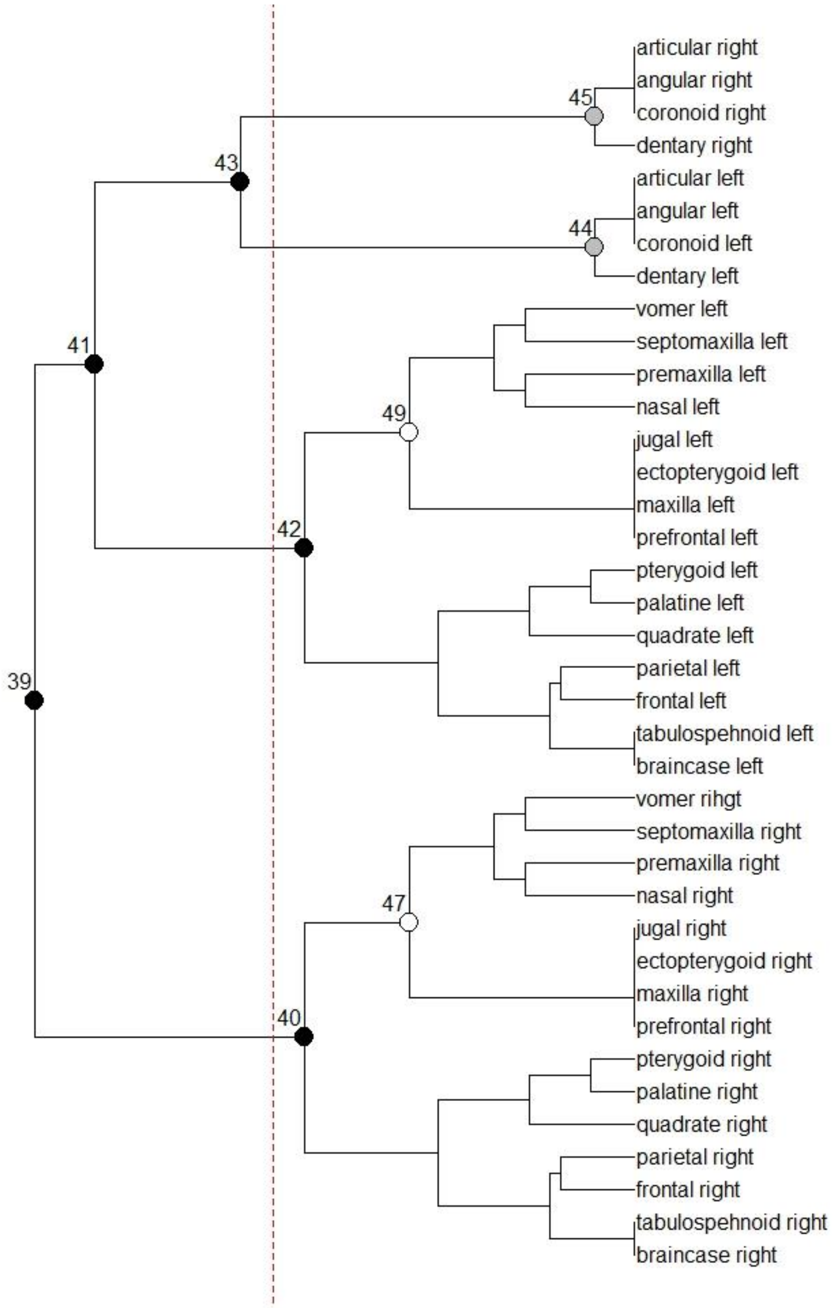

Supplementary Figure 44. The skull network dendrogram of *Rhineura floridana*.

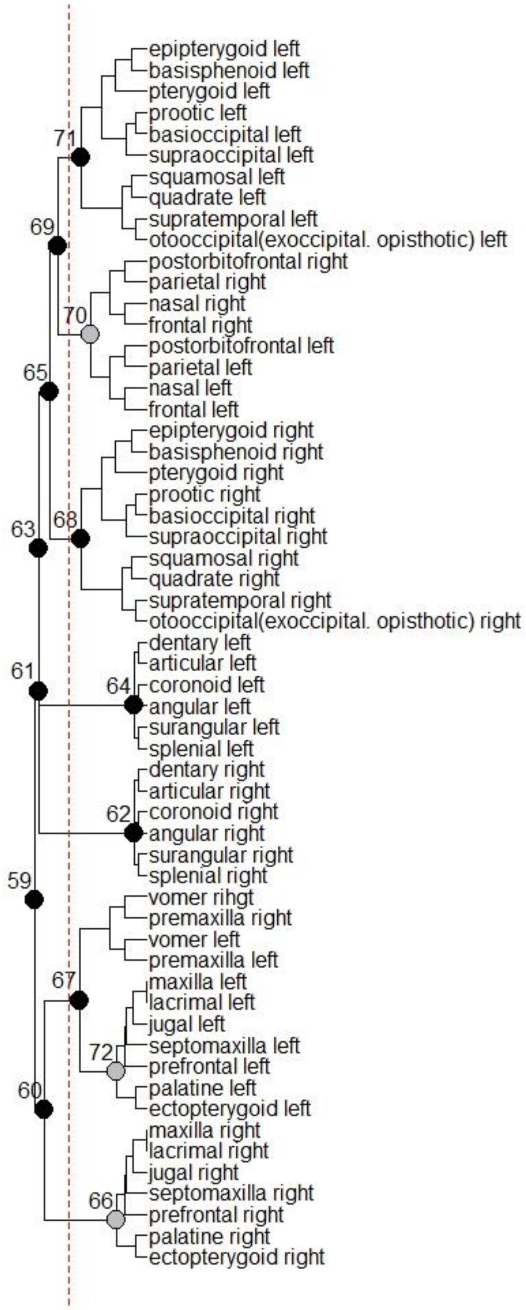

Supplementary Figure 45. The skull network dendrogram of *Shinisaurus crocodilurus*.

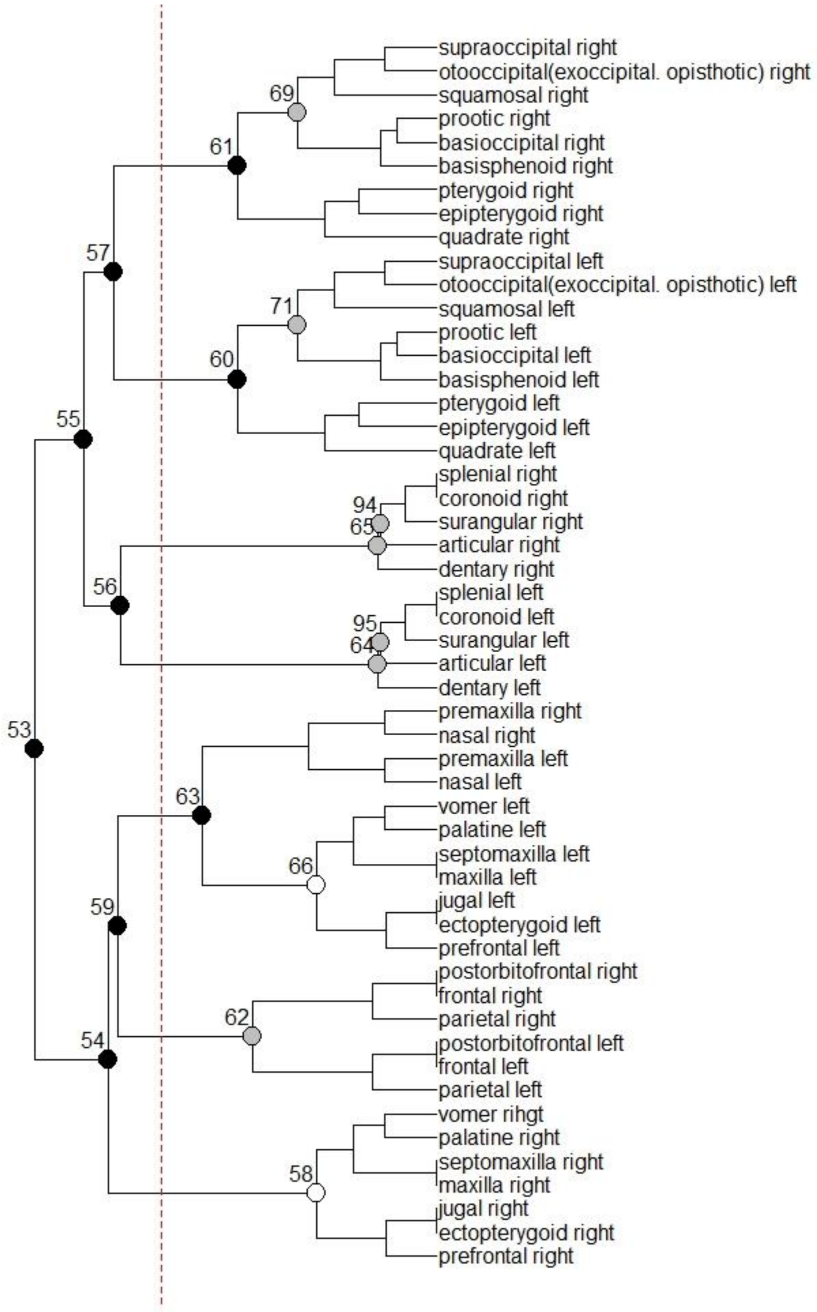

Supplementary Figure 46. The skull network dendrogram of *Strophurus ciliaris*.

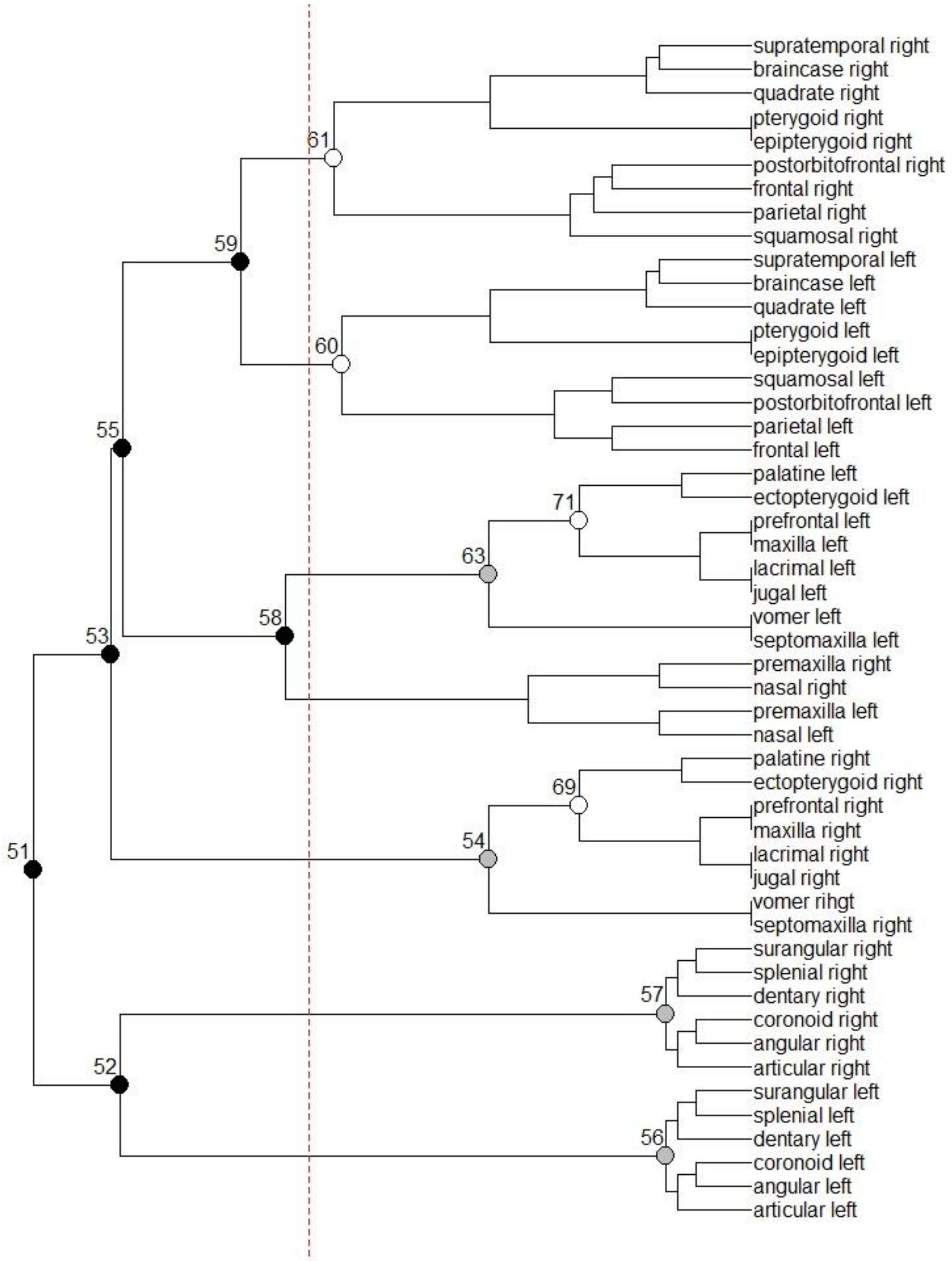

Supplementary Figure 47. The skull network dendrogram of *Takydromus sexlineatus*.

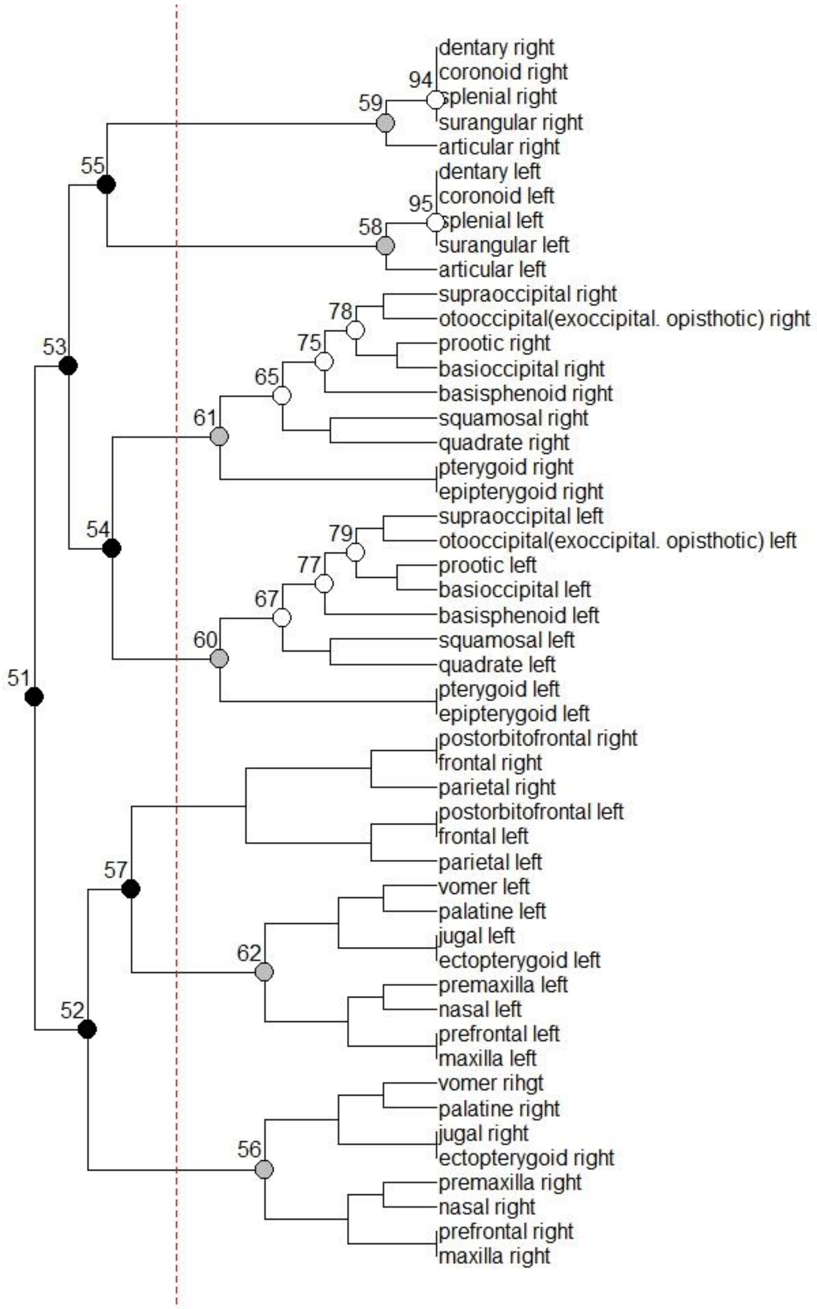

Supplementary Figure 48. The skull network dendrogram of *Tarentola mauritanica*.

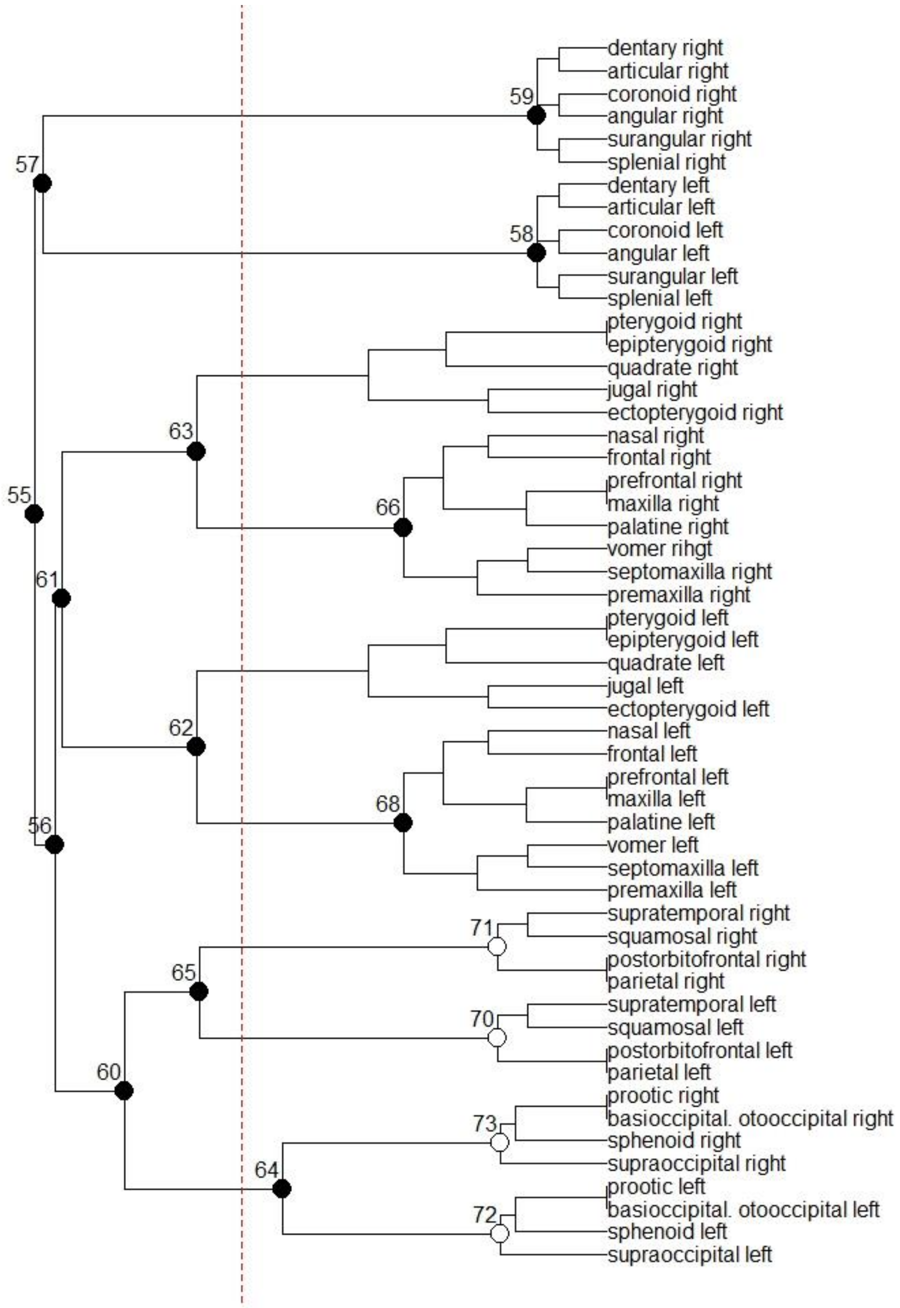

Supplementary Figure 49. The skull network dendrogram of *Trachylepis sulcata*.

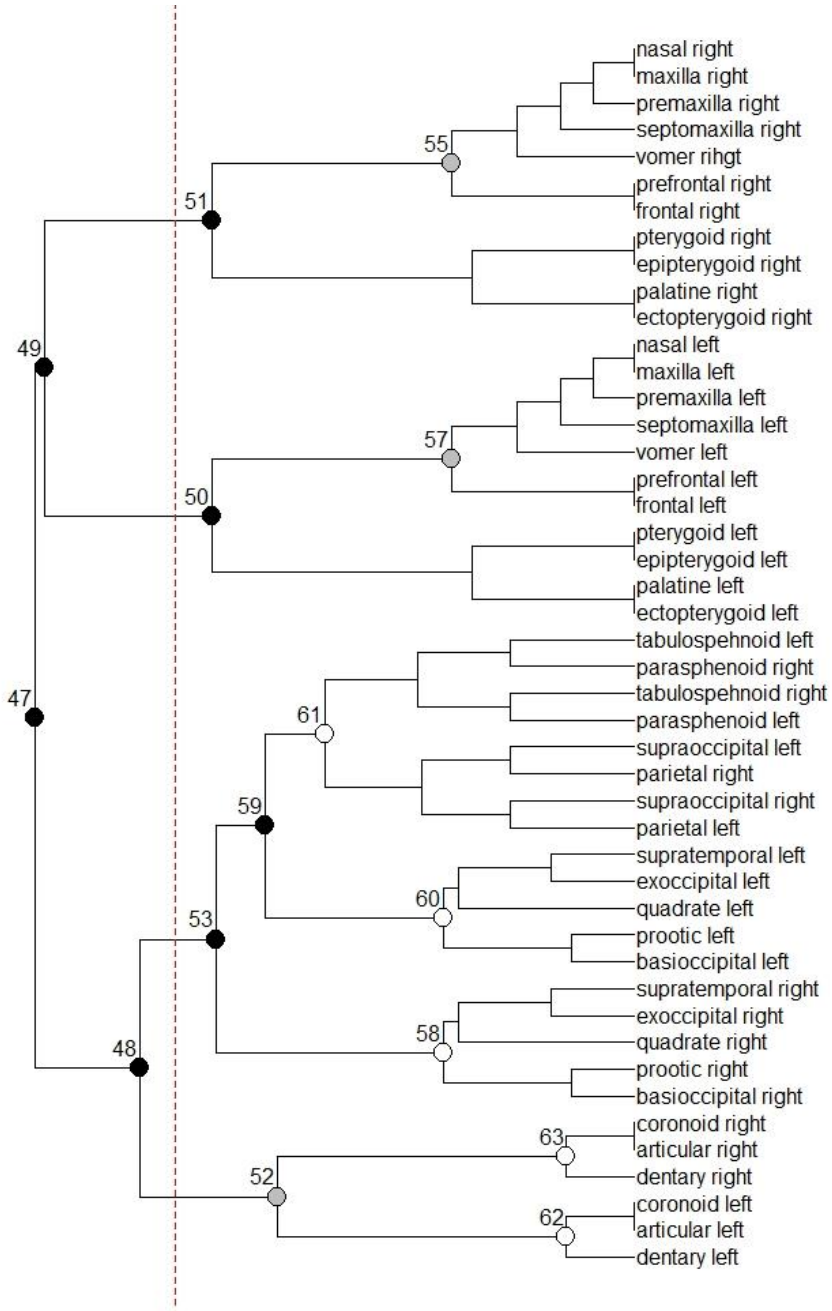

Supplementary Figure 50. The skull network dendrogram of *Trogonophis wiegmanni*.

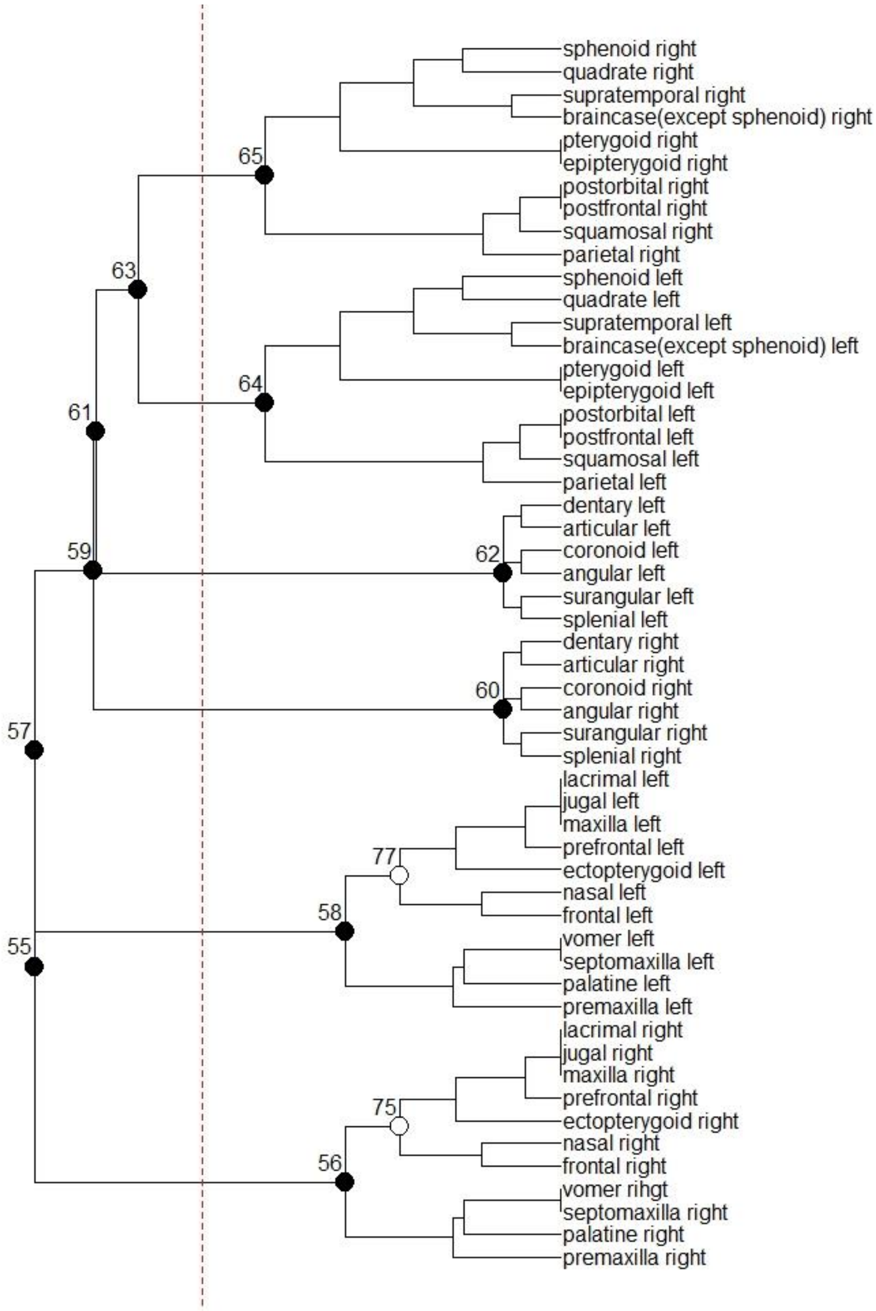

Supplementary Figure 51. The skull network dendrogram of *Tropidophorus cocincinensis*.

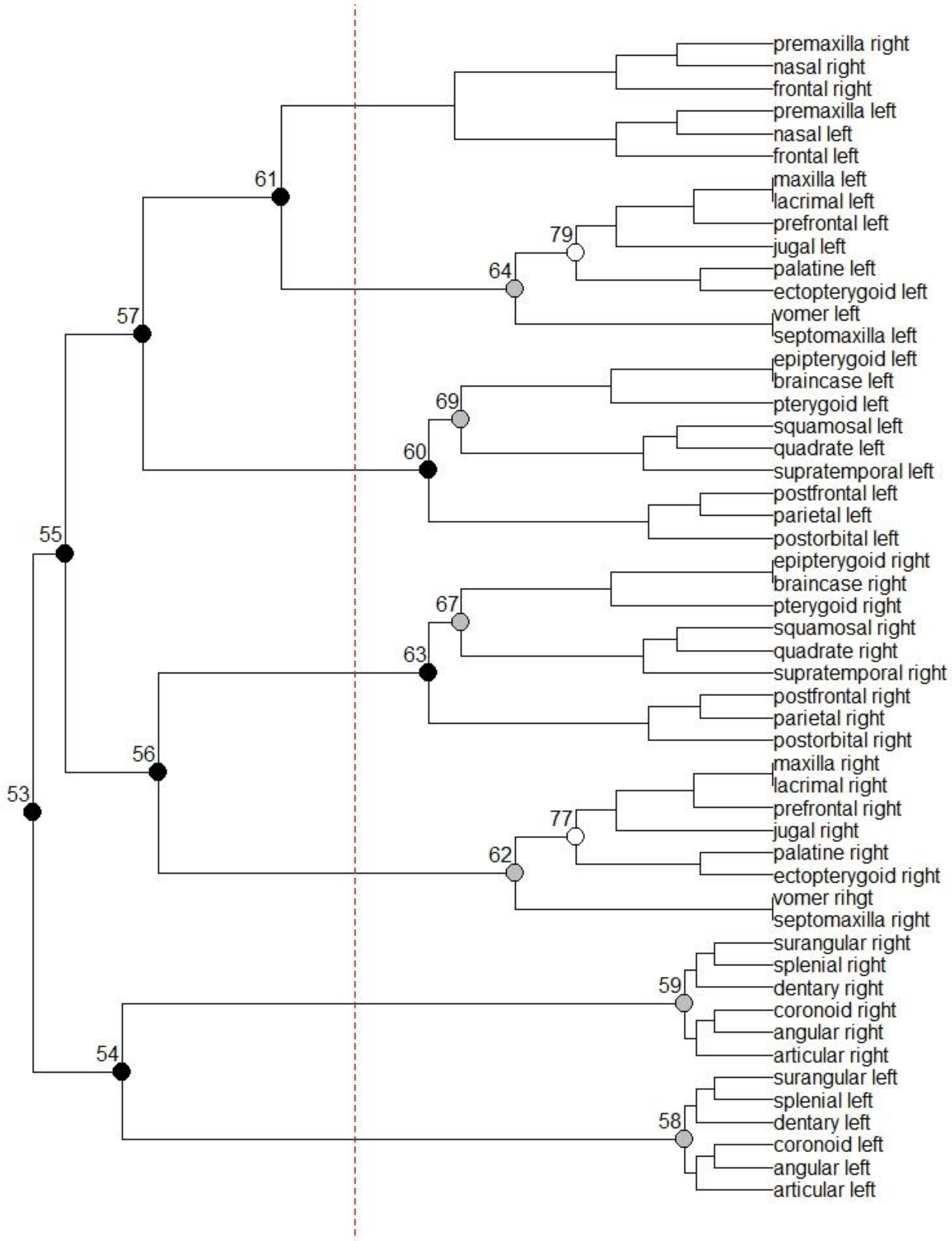

Supplementary Figure 52. The skull network dendrogram of *Tupinambis teguixin*.

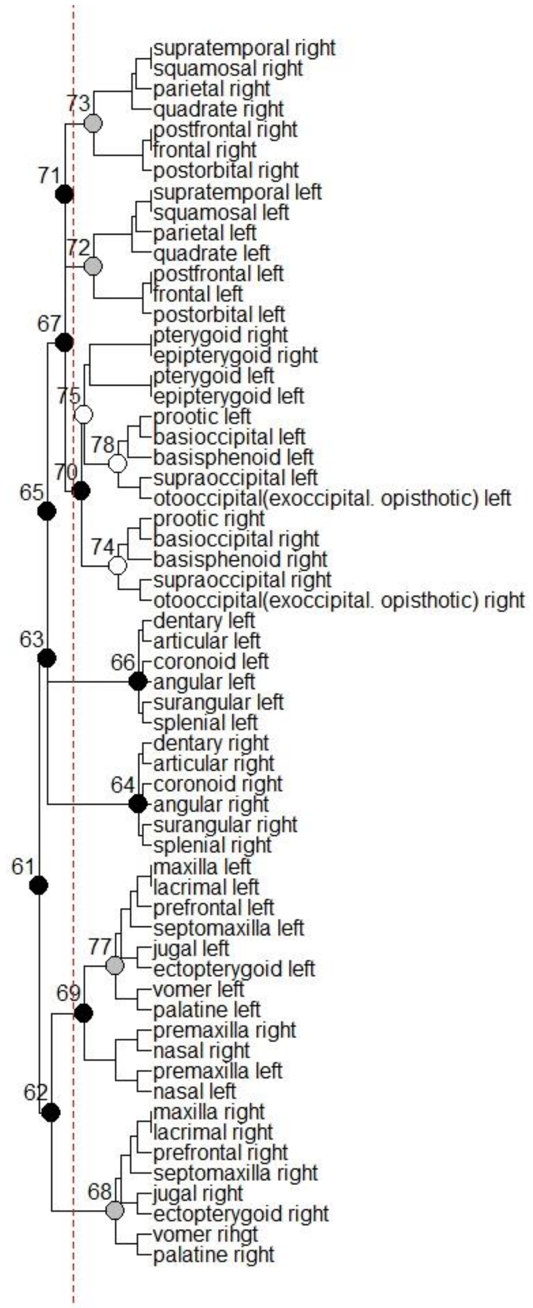

Supplementary Figure 53. The skull network dendrogram of *Urostrophus vautieri*.

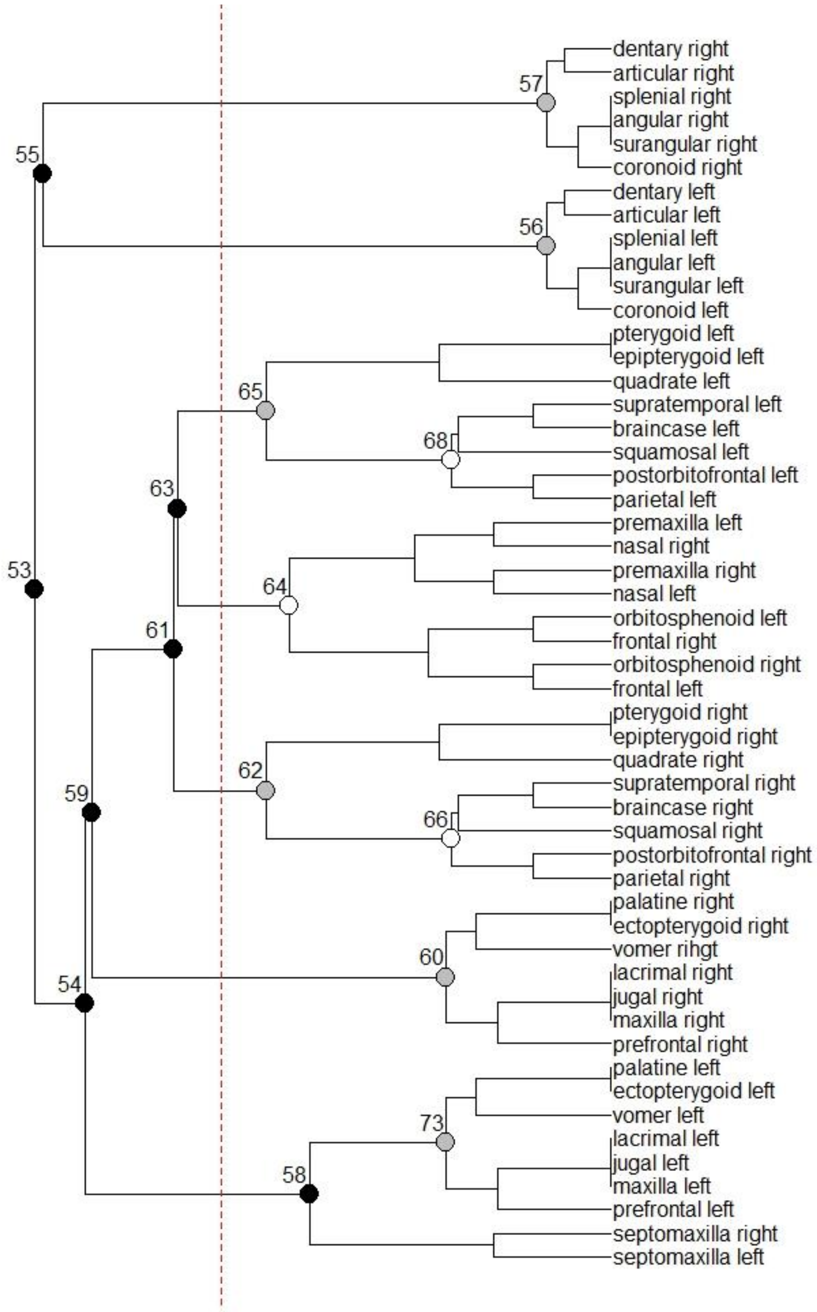

Supplementary Figure 54. The skull network dendrogram of *Varanus komodoensis*.

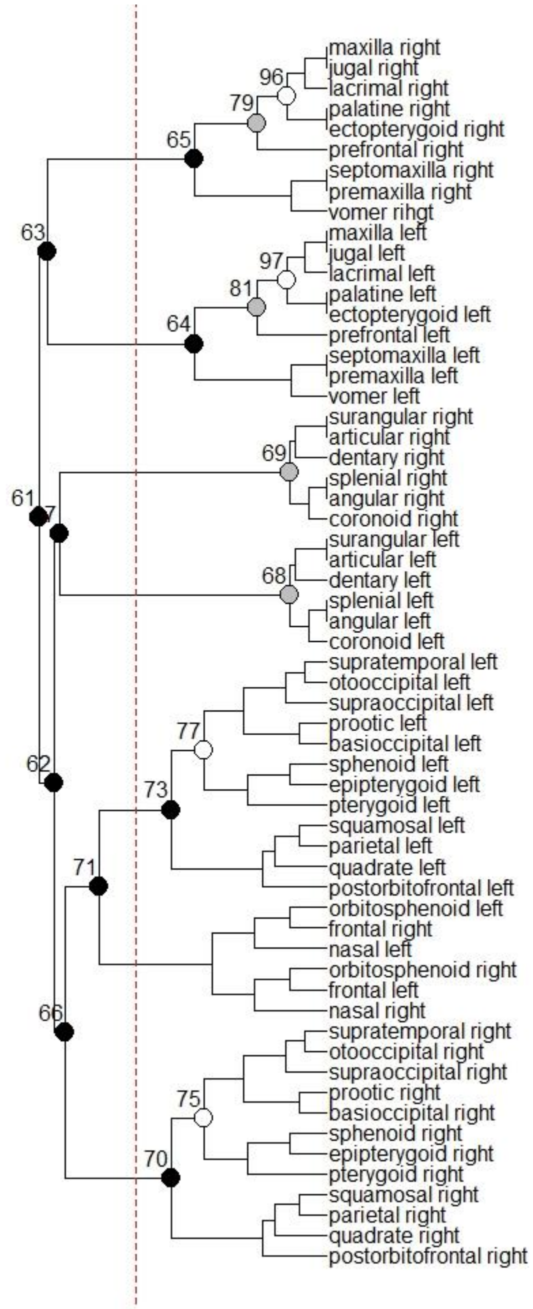

Supplementary Figure 55. The skull network dendrogram of *Varanus niloticus*.

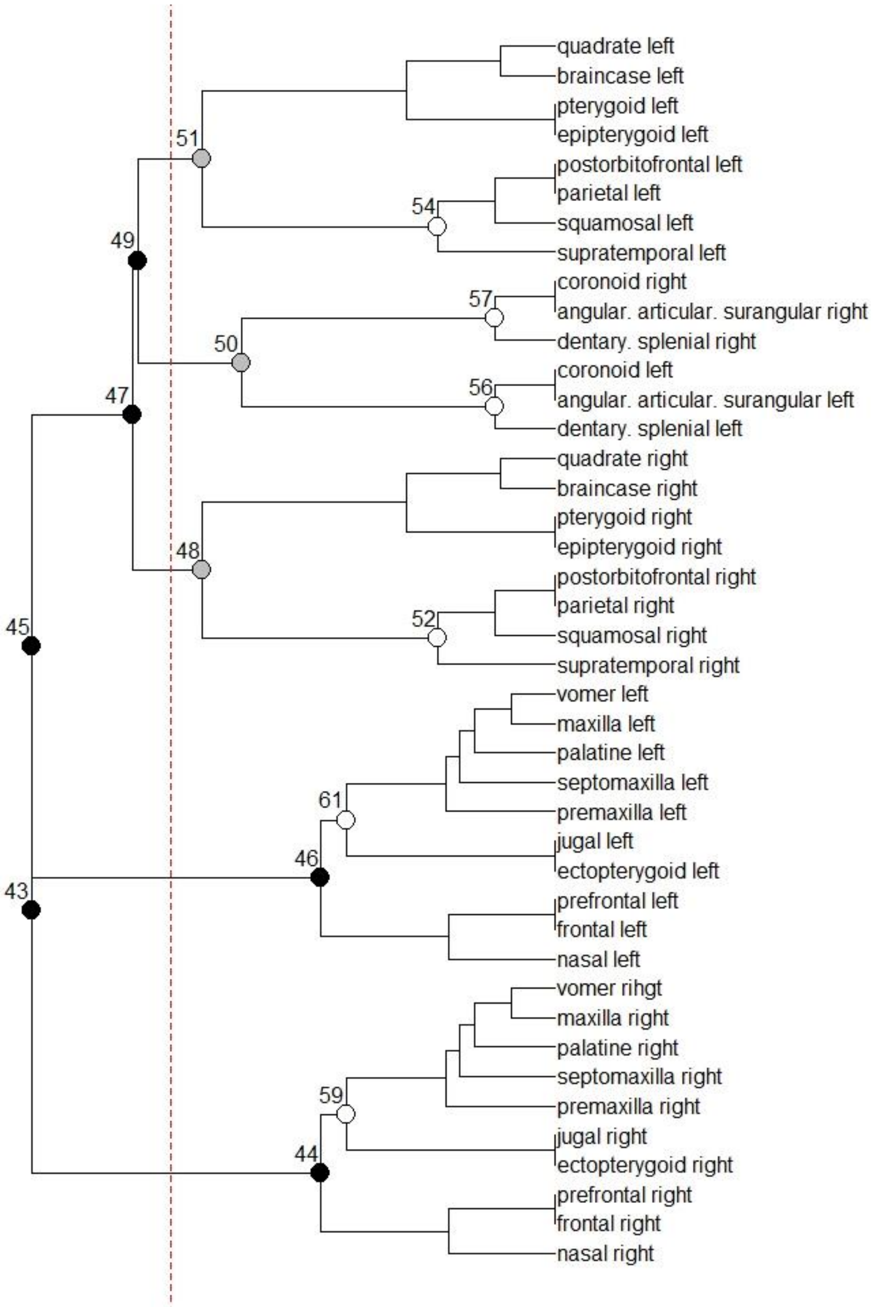

Supplementary Figure 56. The skull network dendrogram of *Xantusia extorris*.

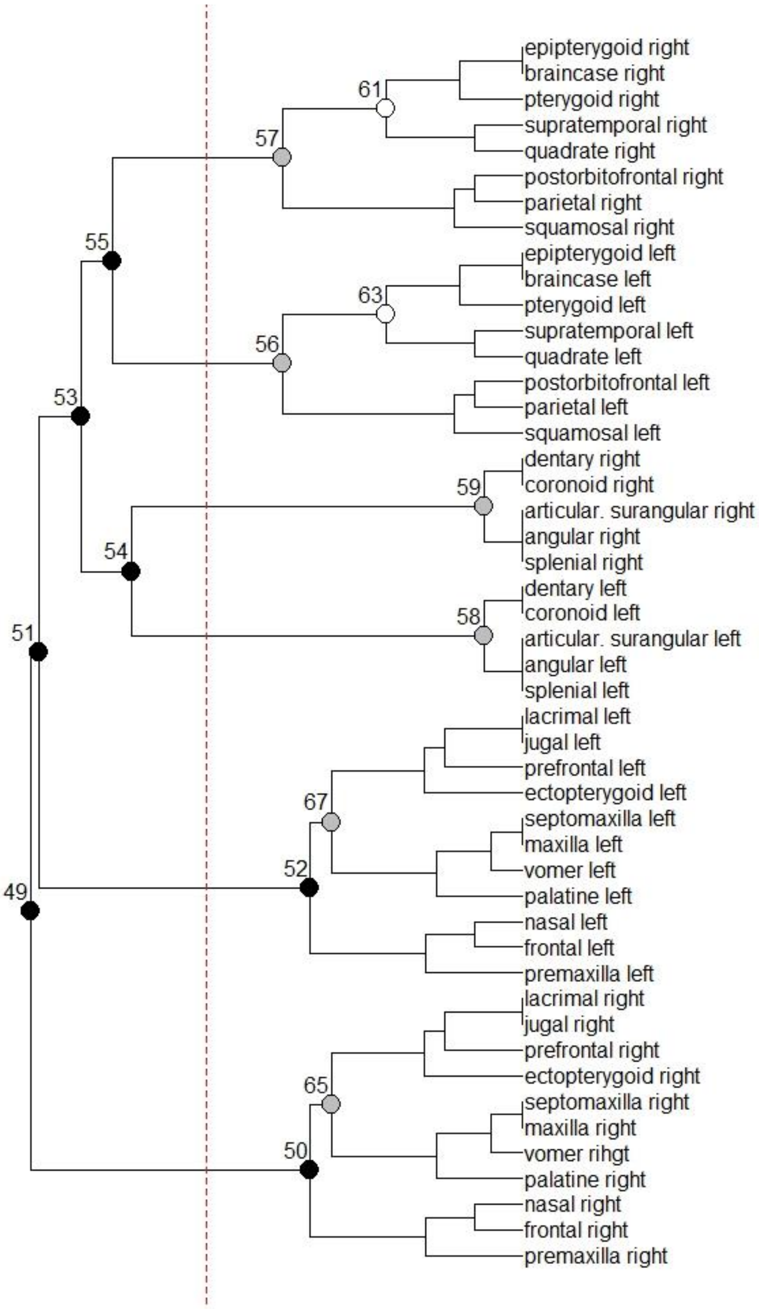

Supplementary Figure 57. The skull network dendrogram of *Xenosaurus grandis*.

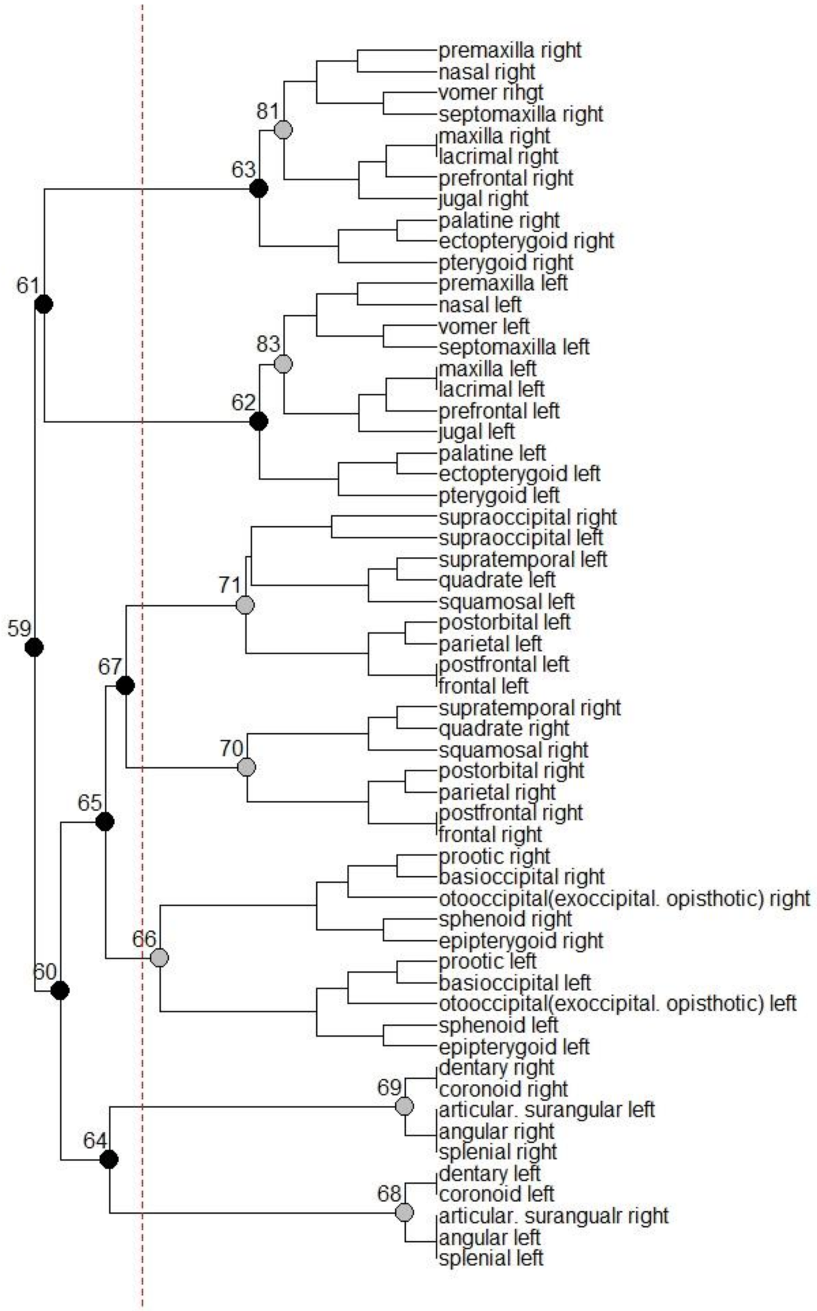

Supplementary Figure 58. The skull network dendrogram of *Zonosaurus ornatus*.
